# A Brazilian Cohort of Patients With Immuno-Mediated Chronic Inflammatory Diseases Infected by SARS-CoV-2 (ReumaCoV-Brasil Registry): Protocol for a Prospective, Observational Study

**DOI:** 10.2196/24357

**Published:** 2020-12-15

**Authors:** Claudia Marques, Adriana Maria Kakehasi, Ana Paula Monteiro Gomides, Eduardo Dos Santos Paiva, Edgard Torres dos Reis Neto, Gecilmara Cristina Salviato Pileggi, José Roberto Provenza, Licia Mota, Ricardo Machado Xavier, Gilda Aparecida Ferreira, Marcelo Medeiros Pinheiro

**Affiliations:** 1 Hospital das Clínicas Universidade Federal de Pernambuco Recife Brazil; 2 Hospital das Clínicas Universidade Federal de Minas Gerais Belo Horizonte Brazil; 3 Faculdade de Medicina Universidade de Brasilia Brasilia Brazil; 4 Hospital de Clínicas Universidade Federal do Paraná Curitiba Brazil; 5 Hospital São Paulo Universidade Federal de São Paulo São Paulo Brazil; 6 Faculdade de Ciências da Saúde de Barretos Barretos Brazil; 7 Pontifícia Universidade Católica de Campinas Campinas Brazil; 8 Hospital de Clínicas Universidade Federal do Rio Grande do Sul Porto Alegre Brazil

**Keywords:** COVID-19, SARS-CoV-2, prospective study, immune-mediated rheumatic diseases, registry, Brazil, inflammatory, chronic disease, cohort, immunology, infection rate, mortality, morbidity

## Abstract

**Background:**

Patients with immune-mediated rheumatic diseases (IMRD) are at increased risk of infections, including significant morbidity and high mortality. Considering the potential for unfavorable outcomes of SARS-CoV-2 infection in patients with IMRD, several questions were raised regarding the impact of COVID-19 at the start of the pandemic.

**Objective:**

This paper presents the protocol of a study that aims to prospectively evaluate patients with IMRD and a confirmed COVID-19 diagnosis (using criteria provided by the Brazilian Ministry of Health).

**Methods:**

The study comprised a prospective, observational cohort (patients with IMRD and COVID-19) and a comparison group (patients with only IMRD), with a follow-up time of 6 months to evaluate differences in health outcomes. The primary outcomes will be changes in IMRD disease activity after SARS-CoV-2 infection at 4 time points: (1) at baseline, (2) within 4-6 weeks after infection, (3) at 3 months after the second assessment (±15 days), and (4) at 6 months (±15 days). The secondary outcomes will be the progression rate to moderate or severe forms of COVID-19, need for intensive care unit admission and mechanical ventilation, death, and therapeutic changes related to IMRD. Two outcomes—pulmonary and thromboembolic events in patients with both IMRD and SARS-CoV-2 infection—are of particular interest and will be monitored with close attention (clinical, laboratory, and function tests as well as imaging).

**Results:**

Recruitment opened in May 2020, with 1300 participants recruited from 43 sites as of November 2020. Patient recruitment will conclude by the end of December 2020, with follow-up occurring until April 2021. Data analysis is scheduled to start after all inclusion data have been collected, with an aim to publish a peer-reviewed paper in December 2020.

**Conclusions:**

We believe this study will provide clinically relevant data on the general impact of COVID-19 on patients with IMRD.

**Trial Registration:**

Brazilian Registry of Clinical Trials RBR-33YTQC; http://www.ensaiosclinicos.gov.br/rg/RBR-33ytqc/

**International Registered Report Identifier (IRRID):**

DERR1-10.2196/24357

## Introduction

### Background

COVID-19 was declared a pandemic on March 11, 2020, by the World Health Organization [1], and on February 26, 2020, Brazil became the first Latin American country to have a confirmed case of COVID-19 [2]. More than 4 million cases and over 130,000 deaths have been confirmed in Brazil as of July 31, 2020 [3].

Patients with immune-mediated rheumatic diseases (IMRD) are at increased risk of infections, including significant morbidity and high mortality [4]. It is worth emphasizing this is a complex binomial with many factors involved, such as disease activity, age, comorbidities, and drugs (eg, glucocorticoids; conventional synthetic, specific target or biological disease–modifying antirheumatic drugs; and immunosuppressants) [5]. Considering a possibly unfavorable evolution of SARS-CoV-2 infection in patients with IMRD, a number of questions has been posed regarding the impact of COVID-19 at the start of the pandemic, including withdrawal or spacing of medications, hospitalization, need of mechanical ventilation, and mortality rate [6-8].

Some Italian, American, French, and Chinese databases have started to demonstrate that the risk of poor outcomes is quite similar to the general population and could be linked more closely with comorbidities and aging than IMRD itself [9-11]. However, there are controversial data, especially regarding mortality rates [12,13]. More recently, a systematic review and meta-analysis showed the prevalence of COVID-19 to be 0.011 (95% CI 0.005-0.0025), which was significantly higher than that of the comparison group [14].

Considering Brazil as a continental country and with relevant regional and socioeconomic differences, as well as discrepancies concerning basic sanitation and access to public and private health care systems, it is important to generate data related to disease activity, treatment management, and survival curves in patients with both IMRD and COVID-19.

### Aims

This paper presents the protocol for the ReumaCoV-Brasil Registry (Brazilian Registry of Patients with Immuno-mediated Chronic Inflammatory Diseases Infected by SARS-CoV-2). This trial was registered with the Brazilian Register of Clinical Trials (RBR-33YTQC) on June 1, 2020.

The primary objective is to prospectively evaluate patients with IMRD who had COVID-19, according to the Brazilian Ministry of Health criteria [15] (Multimedia Appendix 1), and to compare them to a control group of IMRD patients without a COVID-19 diagnosis.

**Figure 1:**
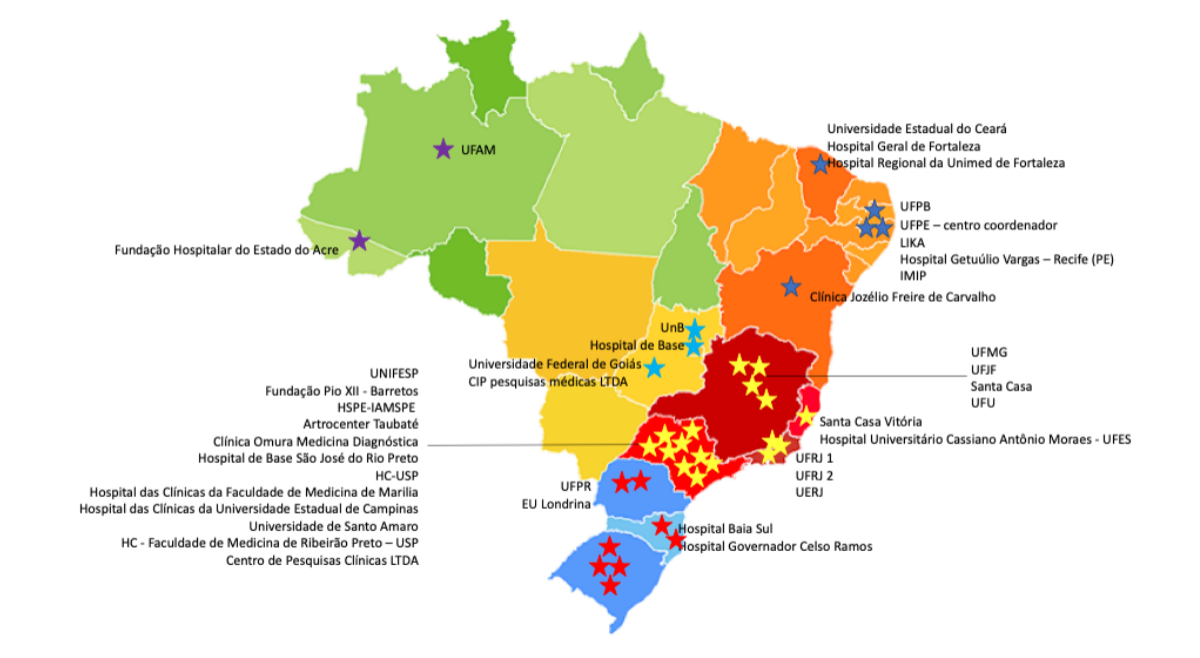
A nationwide task force, comprising 43 centers from 5 geographic regions in Brazil (see full organization names in Textbox 1).

The inclusion phase started on May 20, 2020, with the recruitment of patients from 43 centers in 5 regions of the country (Textbox 1 and Figure 1), and will run through November 30, 2020, considering that Brazil is still undergoing community viral transmission. An invitation to participate in this study was sent to all rheumatologists affiliated with the Brazilian Society of Rheumatology. Most invitees were employed at academic centers or private offices.

**Participating research sites in Brazil.**

- Hospital das Clínicas, Universidade Federal de Pernambuco
- Artrocenter Clínica Médica – Unidade Taubaté, São Paulo
- Centro de Pesquisas Clínicas, São Paulo
- Clínica Jozélio Freire de Carvalho, Bahia
- Clínica Médica do Hospital Baia Sul, Santa Catarina
- Clínica Omura Medicina Diagnóstica, São Paulo
- Faculdade de Ciências da Saúde de Barretos, São Paulo
- Fundação Hospitalar do Estado do Acre
- Fundação Universidade Estadual do Ceará
- Hospital da Secretária da Saúde do Distrito Federal – Instituto Hospital de Base do Distrito Federal
- Hospital das Clínicas da Faculdade de Medicina da USP, São Paulo
- Hospital das Clínicas da Faculdade de Medicina de Marilia, São Paulo
- Hospital das Clínicas da Universidade Estadual de Campinas, São Paulo
- Hospital das Clínicas da Universidade Federal de Goiás
- Hospital das Clínicas da Universidade Federal de Uberlândia, Minas Gerais
- Hospital das Clínicas, Faculdade de Medicina de Ribeirão Preto – USP, São Paulo
- Hospital das Clínicas, Universidade Federal de Minas Gerais
- Hospital de Base, Fundação Faculdade Regional de Medicina São José Do Rio Preto, São Paulo
- Hospital de Clínicas de Porto Alegre, Universidade Federal do Rio Grande do Sul
- Hospital de Clínicas, Universidade Federal do Paraná
- Hospital dos Servidor Público Estadual de São Paulo
- Hospital Geral de Fortaleza, Ceará
- Hospital Getúlio Vargas, Pernambuco
- Hospital Governador Celso Ramos, Santa Catarina
- Hospital Moinhos de Vento, Porto Alegre
- Hospital Nossa Senhora da Conceição, Rio Grande do Sul
- Hospital Regional da Unimed de Fortaleza, Ceará
- Hospital Santa Casa de Misericórdia de Vitória, Espírito Santo
- Hospital São Lucas da PUCRS, Pontifica Universidade Católica do Rio Grande do Sul
- Hospital São Paulo, Universidade Federal de São Paulo
- Hospital Universitário Clementino Fraga Filho (1), Universidade Federal do Rio de Janeiro
- Hospital Universitário Clementino Fraga Filho (2), Universidade Federal do Rio de Janeiro
- Hospital Universitário de Londrina, Universidade Estadual de Londrina, Paraná
- Hospital Universitário Lauro Wanderley, Universidade Federal da Paraíba
- Hospital Universitário, Universidade Federal de Juiz de Fora, Minas Gerais
- Instituto de Medicina Integral Professor Fernando Figueira, Pernambuco
- Laboratório de Imunopatologia Keizo Asamy, Pernambuco
- Santa Casa de Misericórdia de Belo Horizonte, Minas Gerais
- Universidade de Brasília, Distrito Federal
- Universidade de Santo Amaro, São Paulo
- Universidade Estadual do Rio de Janeiro
- Universidade Federal de Ciências da Saúde de Porto Alegre, Rio Grande do Sul
- Universidade Federal do Amazonas

Over the course of 6 months, participants will be questioned regarding disease activity, clinical manifestations of COVID-19 (complaints; evolution [eg, need for hospitalization, intensive care unit, or mechanical ventilation; or death]; time from infection to resolution; epidemiologic, demographic, and socioeconomic data), concomitant medications management, social distancing, flu vaccination, survival curves, and mortality rates.

The secondary objectives will include assessing the autoantibodies profile (rheumatoid factor, anti–cyclic citrullinated peptide [anti-CCP], anti–citrullinated protein antibody, antinuclear antibody, anticardiolipin immunoglobulin [Ig] G and IgM), as well as immunoglobulins (IgM, IgG, and IgA) and the serologic conversion rate for SARS-CoV2 (IgG and IgM).

## Methods

### Study Design

ReumaCoV-Brasil is a multicenter, observational, prospective registry implemented to monitor adult IMRD patients with a confirmed diagnosis of COVID-19, according to the Brazilian Ministry of Health criteria [13].

Using a nationwide sampling strategy, it is a 2-phase study: (1) cross-sectional evaluation (inclusion) with information about previous or current symptoms of COVID-19 and clinical characteristics at baseline, which can be performed via telephone call (preferred due to social distancing measures) or by a face to face visit, if possible; and (2) prospective follow-up concerning the IMRD characteristics with 2 face to face visits, every 3 months (3-month and 6-month assessments), after viral infection.

### Participants and Eligibility Criteria

Regardless COVID-19 diagnosis, eligible patients include those aged 18 years or over with a prior diagnosis of IMRD, according to the American College of Rheumatology or European League against Rheumatism criteria, including rheumatoid arthritis [16,17], systemic lupus erythematosus [18,19], Sjögren syndrome [20], systemic sclerosis [21], inflammatory myopathies [22], axial spondyloarthritis [23-25], enteropathic arthritis [26], psoriatic arthritis [27], sarcoidosis [28], antiphospholipid syndrome [29], Behçet disease [30], mixed connective tissue disease [31], Takayasu arteritis [32,33], giant cell arteritis [34], ANCA (antineutrophil cytoplasm antibodies)-associated vasculitides [35-38], and juvenile idiopathic arthritis in adults [39]. In addition, a third group will also be evaluated, consisting of non-IMRD patients with COVID-19 infection.

The exclusion criteria included the presence of other primary immunodeficiency diseases, past organ or bone marrow transplantation, neoplasms within the last 5 years, current chemotherapy, HIV diagnosis, and thymus diseases. The controls will be patients with IMRD but without a diagnosis of COVID-19, matched for sex, age, and IMRD type. The exclusion criteria stated above will apply to the control group as well.

Considering the moderate to severe forms of COVID-19 as the dependent variable and an estimated rate at 20%, according to the current literature, as well as the proportion of 1 case for 1 control and an error α=5% and β=20%, the sample calculation was approximately 576 IMRD patients with positive COVID-19 and 576 IMRD patients without COVID-19 [1,2,8].

### Clinical Data and Outcomes

When an IMRD case is identified, the patient will be invited to participate in the study and will be enrolled after reading and signing the informed consent form (Multimedia Appendix 2). The clinical form (Multimedia Appendix 3) will be filled at baseline and at 3- and 6-month follow-up using the REDCap platform. Each researcher (principal investigator and 4 additional subinvestigators for each center) will have a unique and personalized login and password to access this platform. The REDCap platform will be frequently monitored in order to ensure data quality.

**Figure 2:**
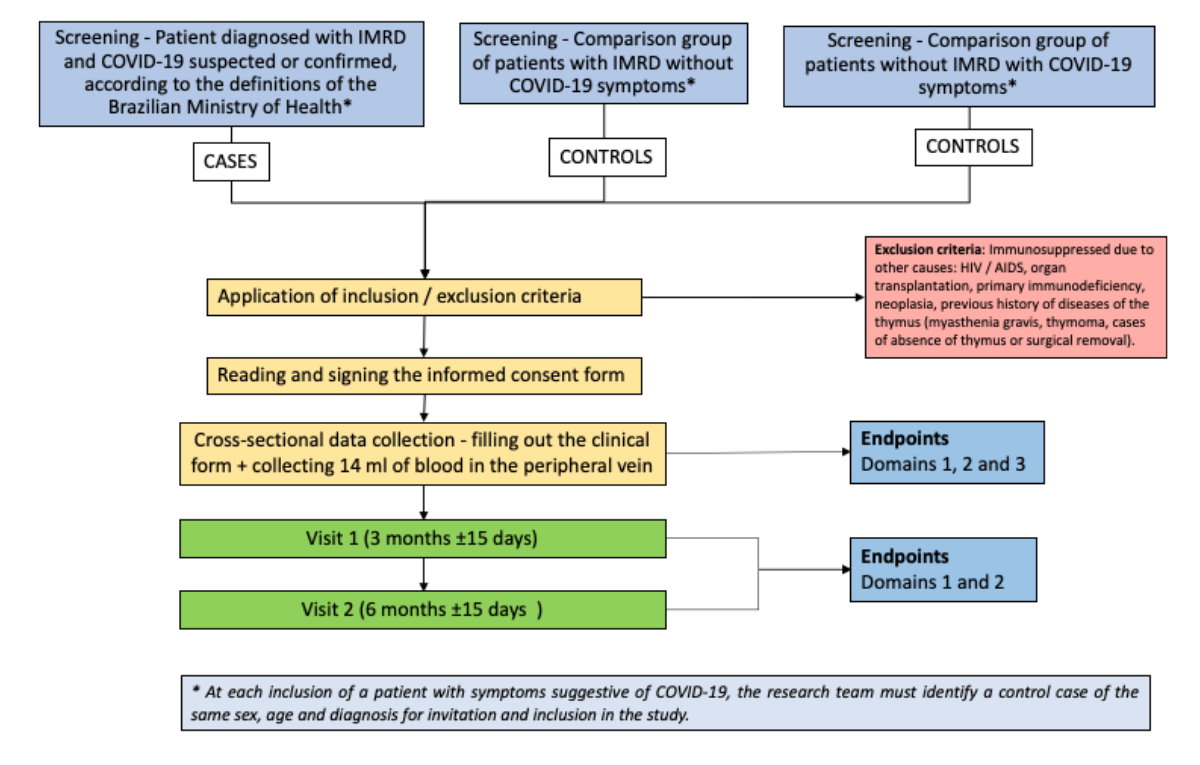
Study flowchart.

The primary outcomes will be changes in IMRD activity after SARS-CoV-2 infection at 4 time points: (1) baseline; (2) within 4-6 weeks after SARS-CoV-2 infection; (3) 3 months after inclusion in the study (±15 days); (4) 6 months after inclusion (±15 days) (Table 1 and Figure 2). If the patient is unavailable to perform a face to face visit at baseline because of social distancing, the physician may use clinical data from within the last 6 months (a period of time without any evidence for COVID-19).

**Table 1:** ReumaCoV-Brasil procedures^a^.

Procedure	Visit 1 (inclusion)		Visit 2 (face to face) – 3 months (±15 days) after visit 1	Visit 3 (face to face) – 6 months (±15 days) after visit 1
	Phone call – immediately after COVID-19 diagnosis	Face to face – within 4-6 weeks after inclusion		
Informal consent	✓			
Inclusion and exclusion criteria	✓	✓		
Signed informed consent		✓		
Clinical data	✓	✓	✓	✓
Disease activity assessment	✓	✓	✓	✓
Lab exams		✓		

^a^The same procedures will be adopted for the control group.

The disease activity assessment will be performed using a global patient assessment and physician assessment, using a numerical visual scale (0-10), as well as specific and validated disease activity measurements or prognostic factors related to systemic vasculitis (Textbox 2). In addition, the type of organ or tissue with systemic prior involvement (eg, the renal, lung, heart, or nervous system [central or peripheral]; hematological, gut, eye, vessels, skin, mucous, and other extra-articular manifestations) will be assessed. For musculoskeletal complaints, including muscle (fatigue, pain, weakness, and myopathy) and joint (arthritis, enthesitis, and dactylitis), endpoints will also be evaluated. The secondary outcomes assessed will be the progression to moderate or severe forms of COVID-19, need for intensive care unit admission and mechanical ventilation, and death.

**Specific and validated disease activity measurements and prognostic factors.**

1. Rheumatoid arthritis
2. Clinical Disease Activity Index [40]
3. Axial spondyloarthritis
4. Ankylosing Spondylitis Disease Activity Score-C-Reactive Protein [41]
5. Ankylosing Spondylitis Disease Activity Score-Erythrocyte Sedimentation Rate [41]
6. Bath Ankylosing Spondylitis Disease Activity Index [42]
7. Psoriatic arthritis
8. Psoriasis Area Severity Index and nail disease [43]
9. Disease Activity in Psoriatic Arthritis [44]
10. Minimal Disease Activity [45]
11. Systemic erythematous lupus
12. Modified Systemic Erythematous Lupus Disease Activity Index [46,47]
13. Sjögren syndrome
14. European League Against Rheumatism Sjögren's Syndrome Disease Activity Index [48]
15. Systemic sclerosis
16. Rodnan score [49]
17. Raynaud’s phenomenon and digital ulcers
18. Behçet disease (BD)
19. Behçet’s Disease Current Activity Form [50]
20. Inflammatory myopathies
21. Manual Muscle Testing in 8 muscles [51]
22. Health Assessment Questionnaire [52]
23. Systemic vasculitis
24. Birmingham Vasculitis Activity Score, version 3 [53]
25. Five-Factor Score [54]

Details about previous lab exams will also be recorded, such as erythrocyte sedimentation rate, C-reactive protein, rheumatoid factor, anti-CCP, antinuclear antibody, anti-ENA (extractable nuclear antigen), anti–double-stranded DNA, human leukocyte antigen B27, complement, anticardiolipin IgG and IgM, ANCA, and cryoglobulins.

The secondary outcomes will be related to COVID-19 itself, such as hospitalization, need of intensive care unit, mechanical ventilation, time from onset of symptoms or hospitalization to development of severe acute respiratory syndrome (SARS), outcome after SARS (improvement or death), laboratory changes (renal function, hemoglobin, leucocytes, lymphocytes, platelets, ferritin, albumin, D-dimer, fibrinogen), and pharmacological (hydroxychloroquine, azithromycin, glucocorticoids, heparin, antibiotics, intravenous immunoglobulin, tocilizumab), experimental (convalescent plasma, extracorporeal membrane oxygenation), and supportive treatments. Moreover, information about changes in IMRD treatment (withdrawal, dosage reduction or addition) and new autoimmune manifestations will be also recorded for all patients.

Two outcomes—pulmonary and thromboembolic events in patients with both IMRD and SARS-CoV-2 infection—are of particular interest and will be monitored with close attention and entail the collection of more data (clinical, lab, function tests, and imaging, when appropriate) because they have been reported as an important aspect of moderate and severe forms of COVID-19, and heparin and direct oral anticoagulants are used to manage them. Considering that some researchers have shown antiphospholipid autoantibodies in patients from the general population without IMRD, another secondary endpoint is to evaluate these aspects as well as to verify if patients with IMRD could have more thromboembolic events compared to the third group (patients without IMRD) [55,56].

The explanatory variables will be age, sex, geographic localization, race, education level, profession, employment situation, comorbidities, IMRD details (disease activity, time since diagnosis, level of damage), concomitant medications, and COVID-19 symptoms (eg, fever, cough, dysgeusia, anosmia, myalgia, fatigue, expectoration, shortage of breath, headache, dizziness, diarrhea, nausea, vomiting), time of infection, municipality, and vaccination against the flu.

If IMRD patients without symptoms of COVID-19 enrolled in the control group test positive for anti–SARS-CoV-2 antibodies (IgM and/or IgG) after scheduled lab exams at baseline, they will be considered as having COVID-19 and will be moved from the control group to the intervention group (asymptomatic group).

### Lab Data Collection

**Figure 3:**
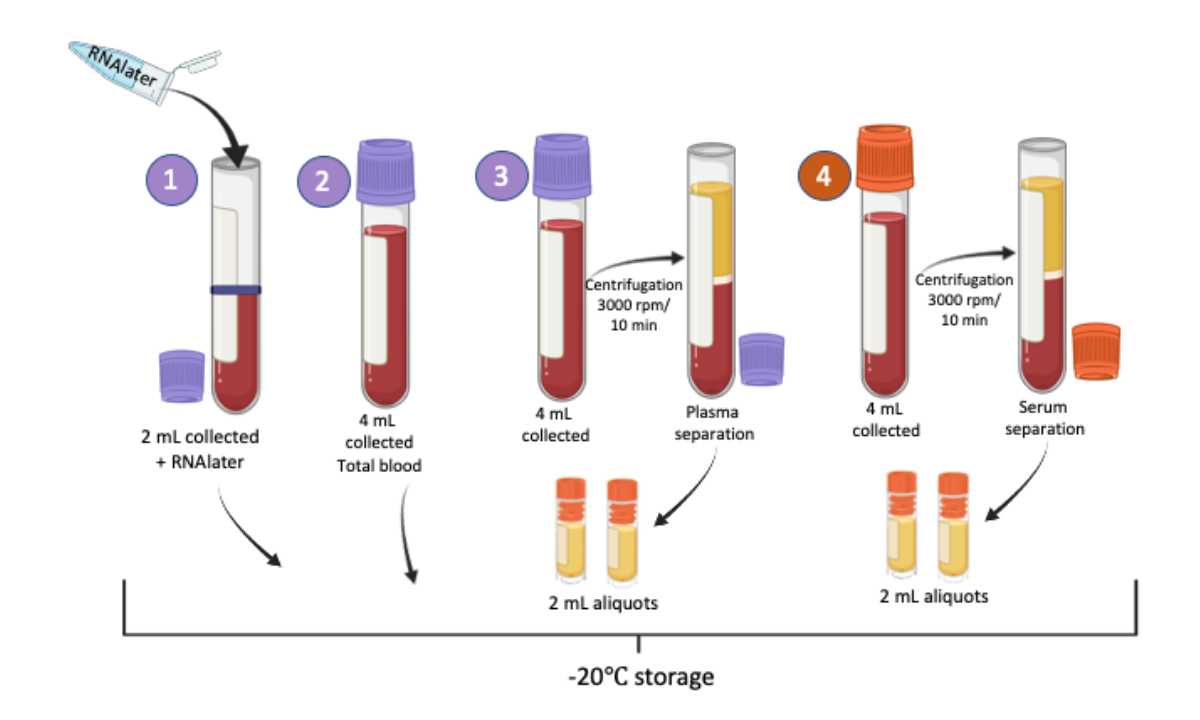
Blood collection and storage protocol: The blood from the peripheral vein will be collected in a total of 16 ml: 3 EDTA tubes, one with 2 ml and two with 4 ml and 1 dry tube with separating gel. To the first EDTA tube, where 2 ml of blood was collected, 2 ml of RNAlater will be added for later storage. The second EDTA tube will be stored with total blood. The third EDTA tube will be centrifuged for plasma separation, along with the dry tube, for serum separation. Serum and plasma will be divided into 2 ml aliquots and frozen at -20ºC, together with the tube where RNAlater was added, and the EDTA tube containing whole blood.

A total of 14 mL of blood will be collected for further lab testing, according to the protocol described in Figure 3. The blood will be centrifuged at 3000 rpm with the serum being separated and stored at –20 °C at each participating center and will be sent to the Hermes Pardini Laboratory posteriorly via one-way shipping. The anti–SARS-CoV-2 antibodies (IgM and IgG) will be evaluated by ELISA (enzyme-linked immunosorbent assay; Euroimmun), using plasma aliquots, according to the manufacturer’s recommendations. Rheumatoid factor, anti-CCP, antinuclear antibody, anticardiolipin (IgG and IgM), and immunoglobulins (IgM, IgG, and IgA) will be tested using serum aliquots and pre-established protocols.

The total blood tube and another tube with RNAlater added will be shipped to the Keizo Asami Immunopathology Laboratory, located at the Federal University of Pernambuco and maintained at –80 °C (Figure 3).

### Data Management and Monitoring

The data will be collected by appropriate and trained health care staff, particularly rheumatologists from public and private backgrounds. In addition, 4 study coordinators will provide regular assistance and training regarding data quality. Regular data inspection and quality control will be performed throughout the lifetime of the study. Research sites will retain identifiable study data securely. The data custodians will work together to establish a suitable trial data repository for the anonymized study data set following the conclusion of primary and secondary data analysis. Anonymization will ensure patients’ privacy and adherence to the previously mentioned guidelines.

### Endpoints

The study’s endpoints are as follows:

- Domain 1: changes in disease activity and modifications in lab exam results
- Domain 2: outcomes concerning moderate and severe forms such as hospitalization rate, need for intensive care unit and mechanical ventilation, total hospitalization time, and death; date of a major serious event (death, hospitalization, survival curve)
- Domain 3: humoral response regarding anti–SARS-CoV-2 antibodies and autoantibodies related to IMRD.

### Statistical Analysis

The data will be analyzed in a descriptive way using absolute and relative frequencies for categorical variables and quantitative measures (mean, median, quartiles, minimum and maximum values, and standard deviation) for numerical variables. The normality of the data will be verified using the Kolmogorov-Smirnov test.

The chi-square association test will be used to assess any differences among the endpoints, or the Fischer exact test for small samples. The linear associations among variables will be evaluated using the Pearson correlation or Spearman, when appropriate.

For the evaluation of the behavior of clinical variables between 2 or more points in time by group, analysis of variance (ANOVA) will be used, with repeated measures to be made. In case of nonnormality of the data, the averages of the groups at each time point will be compared using the Kruskal-Wallis nonparametric test. To compare the means between the groups, the Wilcoxon nonparametric test will be used.

The comparison between the means of numerical variables with normal distribution will be verified through the Student *t* test. In case of violation of the assumption of normality, the Mann-Whitney nonparametric test will be used.

Adjusted multiple linear regression models will be used to assess the simultaneous effects of sex, age, time of disease, disease activity, comorbidities, concomitant medications, and other confounding variables, according to the group and predefined outcomes. For dichotomous dependent variables, a logistic regression model will be preferred. Survival analysis models, including log rank and Kaplan-Meier tests, adjusted for confounding variables, will be developed to assess the main outcomes over time. The time defined as the end date will be the date of a major event, such as illness with confirmation or suspicion of infection, hospitalization, or death.

SPSS, version 20 (IBM Corp), will be used in all analyses, and a *P* value <.05 will be defined as significant.

## Results

### Ethics Approval and Regulatory Considerations

The results of this research will be presented in an aggregated form, guaranteeing confidentiality and ensuring that there are no risks to patients’ well-being and care. This protocol was approved by the Brazilian Committee of Ethics in Human Research on April 5, 2020 (CAAE 30186820.2.1001.8807; number: 3.933.204), and registered on the Brazilian Registry of Clinical Trials (RBR-33YTQC) on June 1, 2020. The project is in the data collection phase, which is expected to end in May 2021.

### Data Availability and Materials

The data are owned and held by the Brazilian Society of Rheumatology (Sociedade Brasileira de Reumatologia). Data can be obtained upon request.

The authors will ensure safe and proper conduct of the study, in agreement with the International Conference on Harmonization Guideline for Good Clinical Practice and the Declaration of Helsinki [57], and reserve the right to audit all study documents and standard operating procedures at the coordinating center and research sites.

## Discussion

The ReumaCoV-Brasil is a Brazilian registry of rheumatic patients with COVID-19, supported by the Brazilian Society of Rheumatology. This study was designed to provide more information on epidemiological data and specific outcomes in Brazilian patients with IMRD across the country. In addition, the immunophenotyping may help us to understand the different outcomes according to genetic background and host response [58,59]. With an unprecedent design and a real-life perspective, particularly focused on disease activity before and 6-month follow-up after COVID-19 diagnosis, ReumaCoV-Brasil will add new evidence regarding the complex interaction between SARS-CoV-2 and IMRD. In addition, our database will be linked to other national databases for enhancing the magnitude of captured data and to avoid missing data. However, some limitations may occur, such as potential biases, including notification of more severe cases and lack of confirmatory tests (SARS-CoV-2 RNA [ribonucleic acid] real-time polymerase chain reaction) in asymptomatic patients or with nonsevere forms. In addition, our data will allow comparisons with other international registries and provide enhanced knowledge about similarities and differences across countries. Another relevant point is related to case definitions that varied among countries and could contribute to the differences in the case fatality rates in affected regions. A clear definition of a COVID-19 case is crucial for management, tracking of clinical illness, and to inform the quarantine measures and social distancing during the COVID-19 outbreak [60]. For our registry, we choose suitable criteria to avoid problems concerning outcome definitions. We believe this study will provide clinically relevant data on the general impact of COVID-19 on patients with IMRD.

## Appendix

Multimedia Appendix 1 Definitions of symptomatic suspected and confirmed cases and asymptomatic cases of COVID-19, according to the Brazilian Ministry of Health criteria.

Multimedia Appendix 2 Informed consent form (in portuguese).

Multimedia Appendix 3 Clinical form - generate using the REDCAp (Research Electronic Data Capture) platform (available from: http://www.project-redcap.org).

## Abbreviations

ANCA: antineutrophil cytoplasm antibodies
ANOVA: analysis of variance
anti-CCP: anti–cyclic citrullinated peptide
ELISA: enzyme-linked immunosorbent assay
ENA: extractable nuclear antigen
Ig: immunoglobulin
IMRD: immune-mediated rheumatic diseases
RNA: ribonucleic acid
SARS: severe acute respiratory syndrome
