# Supplementary figures and images for: A Brazilian Cohort of Patients With Immuno-Mediated Chronic Inflammatory Diseases Infected by SARS-CoV-2 (ReumaCoV-Brasil Registry): Protocol for a Prospective, Observational Study
Source: JMIR Res Protoc. 2020 Dec 15;9(12):e24357. doi: 10.2196/24357 (PMC7744142; doi:10.2196/24357)

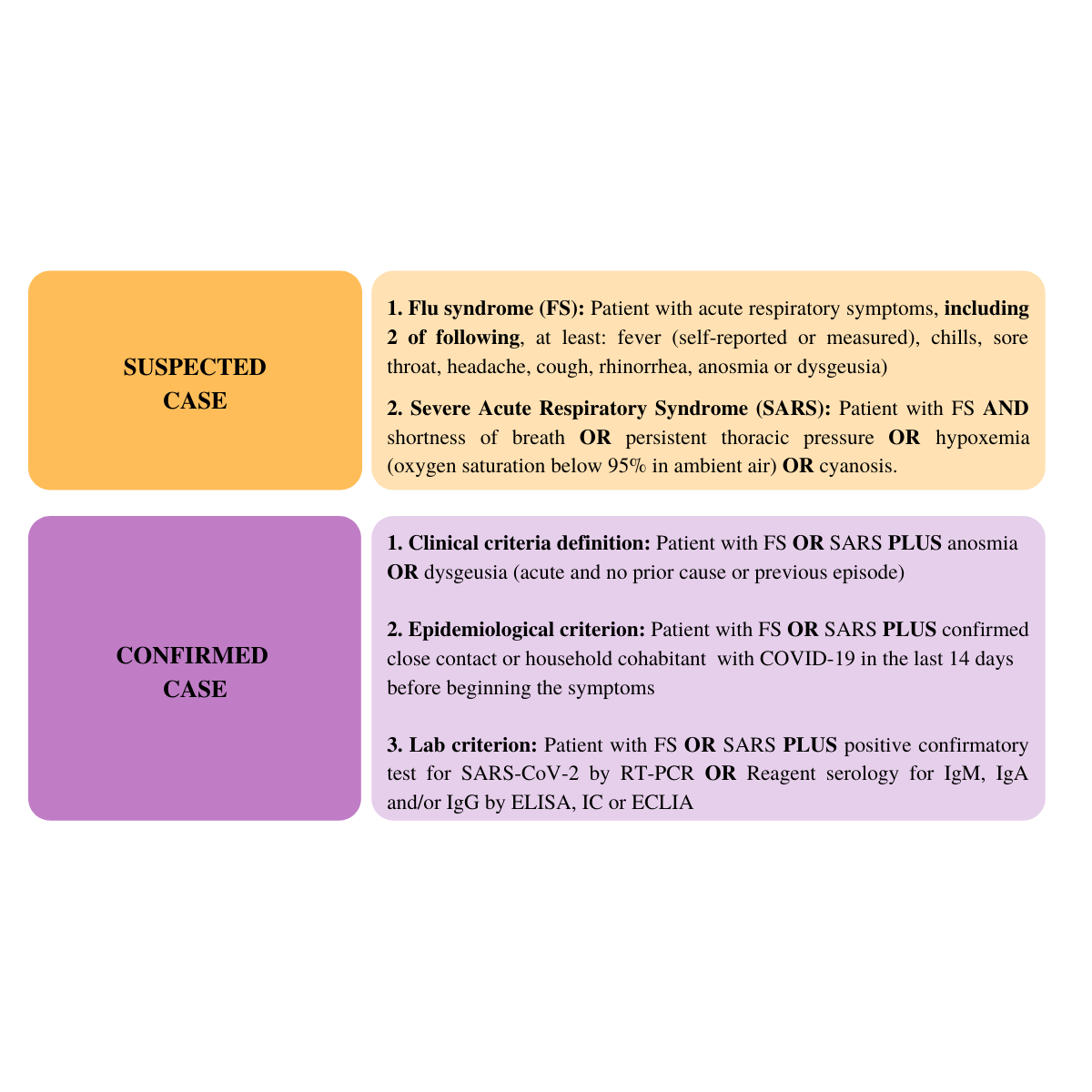

Supplement: Multimedia Appendix 1 [file resprot_v9i12e24357_app1.png]
