# Supplementary material for: A Brazilian Cohort of Patients With Immuno-Mediated Chronic Inflammatory Diseases Infected by SARS-CoV-2 (ReumaCoV-Brasil Registry): Protocol for a Prospective, Observational Study
Source: JMIR Res Protoc. 2020 Dec 15;9(12):e24357. doi: 10.2196/24357 (PMC7744142; doi:10.2196/24357)
Supplement: Multimedia Appendix 2 [file resprot_v9i12e24357_app2.pdf]

# TERMO DE CONSENTIMENTO LIVRE E ESCLARECIDO

Convidamos o (a) Sr. (a) para participar como voluntário (a) da pesquisa **ESTUDO BRASILEIRO DE PACIENTES COM DOENÇAS INFLAMATÓRIAS CRÔNICAS IMUNOMEDIADAS INFECTADOS PELO NOVO CORONA VÍRUS 2019 (SARS-CoV-2),** que está sob a responsabilidade do (a) pesquisador (a) CLAUDIA DINIZ LOPES MARQUES, endereço Avenida Professor Moraes Rego, s/n – Hospital das Clinicas – UFPE – Fone: (81) 21265757 e (81) 992945459. Também participam deste estudo os pesquisadores: Adriana Maria Kakehasi, Universidade Federal de Minas Gerais, Fone: (31) 99925-2662; Ana Paula Monteiro Gomides, Universidade Federal de Brasília, Fone: (61) 99339-9393; Eduardo Paiva, Universidade Federal do Paraná, Fone: (41) 991551998; Gecilmara Pillegi, Santa Casa de Misericordia de Barretos, Fone: (16) 98837-1707; Lícia Maria Henrique da Mota, Universidade de Brasília, Fone: (61) 99221-5598; Marcelo Pinheiro, Universidade Federal de São Paulo; Fone: (11) 99186-7693; Mariana Peixoto Souza, Santa Casa de Belo Horizonte, Fone: (31) 99325-9162; Ricardo Machado Xavier, Universidade Federal do Rio Grande do Sul, Fone: (51) 99698-5501; Ana Karla Guedes, Hospital Universitário Lauro Wanderly, UFPB; F: (83) 98680-2040; Rina Dalva Giorgi, Hospital dos Servidores de São Paulo, F: (11) 99290-7114; Viviane Angelina de Souza, Universidade Federal de Juiz de Fora, F: (32) 99991-9523 e Danyelly Bruneska, LIKA/UFPE, F: (81)98842-5594.

Todas as suas dúvidas podem ser esclarecidas com o responsável por esta pesquisa. Apenas quando todos os esclarecimentos forem dados e você concorde com a realização do estudo, pedimos que rubrique as folhas e assine ao final deste documento, que está em duas vias. Uma via lhe será entregue e a outra ficará com o pesquisador responsável.

Você estará livre para decidir participar ou recusar-se. Caso não aceite participar, não haverá nenhum problema, desistir é um direito seu, bem como será possível retirar o consentimento em qualquer fase da pesquisa, também sem nenhuma penalidade.

## INFORMAÇÕES SOBRE A PESQUISA:

- **Descrição da pesquisa**: Você foi convidado a fazer parte desta pesquisa por ser acompanhado neste hospital com diagnóstico de uma doença reumatológica imunomediada (provocadas por alterações do sistema imunológico), como artrite reumatoide, lúpus eritematoso sistêmica, síndrome de Sjogren, esclerose sistêmica, miopatias inflamatórias, artrite idiopática juvenil, doença mista do tecido conjuntivo, espondiloartrite, vasculites e por estar apresentando ou ter apresentado sintomas da gripe causada pelo novo coronavírus (SARS-CoV-2), em uma doença conhecida como COVID-19. Os sintomas são febre, tosse com expectoração (catarro), falta de ar, dor de cabeça, sensação de fraqueza no corpo, diarreia, náuseas ou vômitos. Você também poderá ser convidado a participar, caso apresente uma destas doenças, mas não tenha apresentado nenhum sintoma de gripe nas últimas semanas, para compor um grupo sem o vírus, e comparar com os resultados das pessoas que tem o vírus.

  O objetivo deste estudo é acompanhar a evolução de pacientes com as doenças reumáticas citadas acima, infectados pelo novo corona vírus 2019 (SARS-Cov-2) e verificar se há influência de características do paciente (idade, sexo e presença de outras doenças como hipertensão, diabetes, doença nos pulmões, etc), uso do remédios utilizados para tratar a sua doença e da própria doença reumática sobre a evolução da infecção, ou seja, queremos entender o que o vírus provoca em pessoas que tem a sua doença. Os medicamentos que você toma são importantes para o controle da sua doença, e ainda não se conhece o efeito dos remédios sobre o vírus., como também não se sabe o que o vírus pode provocar em pessoas com doenças reumáticas. Por este motivo é fundamental estudar como essas pessoas reagem com a infecção. Esse conhecimento pode melhorar a assistência médica e colaborar com as ações da saúde pública brasileira nesse cenário de pandemia, que

significa que a infecção está disseminada no mundo todo.

Para participar será necessário, após a leitura e assinatura deste documento, que você responda algumas perguntas sobre os sintomas da gripe (caso tenha apresentado), sobre a sua doença reumatológica e sobre os remédios que você toma. Em seguida, faremos a coleta de 20 ml de sangue (o equivalente a 2 colher de sopa) em uma veia do seu braço, para estudar melhor como a doença reage no seu organismo e para confirmar se você teve COVID-19. Além disso, serão analisados os dados obtidos no prontuário referentes à sua história médica, ao exame físico e aos dados de exames laboratoriais e radiológicos, avaliados pelos pesquisadores do seu hospital de referência. Os procedimentos do estudo – coletas de informações relativas à sua doença e tratamento, exames laboratoriais, exames radiológicos, coleta de sangue, preenchimento de escores de atividade de doença – não são diferentes daqueles que você faria como parte da avaliação e seguimento de rotina com o seu médico.

Pode ser que você já tenha fornecido seu consentimento verbal para realização da primeira entrevista por telefone, realizada pelo seu médico. Caso isso tenha acontecido, e você ainda deseje continuar participando desta pesquisa, ao assinar este documento você concorda que os dados coletados durante a entrevista por telefone também sejam utilizados na pesquisa. Caso contrário, você será excluído da pesquisa e seus dados não serão utilizados, não havendo nenhum tipo de penalidade para o seu acompanhamento neste ambulatório.

- **Período de participação no estudo:** após a primeira avaliação, que pode ser presencialmente ou ter sido realizada por telefone, você deverá retornar para nova consulta após 3 meses e 6 meses, onde serão realizados os mesmos procedimentos, com exceção da coleta de sangue. Só haverá mudança de tratamento caso você venha a apresentar piora da sua doença, mas isso não faz parte dos procedimentos da pesquisa. Essa modificação será feita da mesma forma como é feito nas consultas de rotina.
- **Riscos para o voluntário da pesquisa:** durante sua participação neste estudo você tem o risco de ser constrangido (envergonhado) por conta da entrevista ou pela demora durante a realização dos procedimentos da pesquisa. Para evitar que isso aconteça você será atendido em uma sala fechada, com ar-condicionado, com a presença apenas do pesquisador que irá realizar a entrevista, e seu atendimento e coleta de sangue serão priorizados. A coleta de sangue será realizada em uma veia do seu braço, com agulha e seringa esterilizadas, mas pode haver o risco de hematoma (mancha roxa) ou sangramento local. Para reduzir esse risco a coleta será realizada por uma pessoa com experiência. Caso você venha a apresentar essas alterações, você deverá fazer compressas de água quente em casa e se não melhorar, deve procurar o responsável por esta pesquisa no hospital ou através do telefone listado acima (pode fazer ligações a cobrar). Outro risco que você corre é que sua identidade seja revelada, pois será feita consulta no seu prontuário, e este pode ser extraviado (perdido). Para evitar isso, o prontuário será consultado apenas no hospital.
- **Benefícios para o voluntário da pesquisa:** Os benefícios atribuídos ao estudo são coletivos, pois através dos resultados obtidos será possível um melhor entendimento da doença e melhor condução dos casos de pacientes com DRIM infectados pelo SARS-CoV-2. Do ponto de vista individual os pacientes terão o benefício de um acompanhamento mais detalhado de sua doença diante da suspeita da COVID-19.
- **Sobre armazenamento e utilização de material biológico:** o sangue que será coletado no seu braço servirá para que seja confirmado que você foi infectado pelo novo coronavírus, para avaliar a presença de autoanticorpos e dosagem de imunoglobulinas. Em alguns centros onde seja possível o envio do sangue em até 24h, também será realizada um estudo de como se comportam os linfócitos (células de defesa). Após a realização do exame, o sangue que sobrar não será utilizado em nenhuma outra pesquisa.

Todas as informações desta pesquisa serão confidenciais e serão divulgadas apenas em eventos ou publicações científicas, não havendo identificação dos voluntários, a não ser entre os responsáveis pelo estudo, sendo assegurado o sigilo sobre a sua participação. Os dados coletados nesta pesquisa nas fichas

clínicas durante a entrevista, ficarão armazenados em pastas e no computador pessoal, sob a responsabilidade do pesquisador, no endereço acima informado, pelo período de mínimo 5 anos.

O Sr./Sra. poderá solicitar, se assim quiser, o relatório final da pesquisa que fez parte. Também, cópias de todos os resultados dos exames complementares realizados nesta pesquisa poderão ser solicitadas ao pesquisador.

Nada lhe será pago e nem será cobrado para participar desta pesquisa, pois a aceitação é voluntária, mas fica também garantida a indenização em casos de danos, comprovadamente decorrentes da participação na pesquisa, conforme decisão judicial ou extra-judicial. Se houver necessidade, as despesas para a sua participação serão assumidas pelos pesquisadores (ressarcimento de transporte e alimentação).

Em caso de dúvidas relacionadas aos aspectos éticos deste estudo, você poderá consultar o Comitê de Ética em Pesquisa Envolvendo Seres Humanos do HC/UFPE no endereço: **(Avenida Prof. Moraes Rego s/n – 3º Andar- Cidade Universitária, Recife-PE, Brasil CEP: 50670-420, Tel.: (81) 2126.3743 – e-mail: cephcufpe@gmail.com).**

______________________________________________
(assinatura do pesquisador)

**CONSENTIMENTO DA PARTICIPAÇÃO DA PESSOA COMO VOLUNTÁRIO (A)**

Eu, _____________________________________, CPF _________________, abaixo assinado, após a leitura (ou a escuta da leitura) deste documento e de ter tido a oportunidade de conversar e ter esclarecido as minhas dúvidas com o pesquisador responsável, concordo em participar do **ESTUDO BRASILEIRO DE PACIENTES COM DOENÇAS INFLAMATÓRIAS CRÔNICAS IMUNOMEDIADAS INFECTADOS PELO NOVO CORONA VÍRUS 2019 (SARS-CoV-2)** como voluntário (a). Fui devidamente informado (a) e esclarecido (a) pelo(a) pesquisador (a) sobre a pesquisa, os procedimentos nela envolvidos, assim como os possíveis riscos e benefícios decorrentes de minha participação. Foi-me garantido que posso retirar o meu consentimento a qualquer momento, sem que isto leve a qualquer penalidade (ou interrupção de meu acompanhamento/ assistência/tratamento).

Local e data __________________
Assinatura do participante: __________________________

Impressão digital (opcional)

**Presenciamos a solicitação de consentimento, esclarecimentos sobre a pesquisa e o aceite do voluntário em participar.** (02 testemunhas não ligadas à equipe de pesquisadores):

| | |
|---|---|
| Nome: | Nome: |
| Assinatura: | Assinatura: |
