# Supplementary material for: A Brazilian Cohort of Patients With Immuno-Mediated Chronic Inflammatory Diseases Infected by SARS-CoV-2 (ReumaCoV-Brasil Registry): Protocol for a Prospective, Observational Study
Source: JMIR Res Protoc. 2020 Dec 15;9(12):e24357. doi: 10.2196/24357 (PMC7744142; doi:10.2196/24357)
Supplement: Multimedia Appendix 3 [file resprot_v9i12e24357_app3.pdf]

2020-0161 - Ricardo Xavier - ESTUDO BRASILEIRO DE PACIENTES COM DOENCAS INFLAMATORIAS CRONICAS IMUNOMEDIADAS INFECTADOS PELO NOVO CORONA VÍRUS 2019 (Severe Acute Respiratory Syndrome Coronavirus 2 - SARS-CoV-2) REUMACOV BRASIL

Codebook

Codes for Missing Data	
**Code / Value**	**Label**
ND	Não disponível

Data Dictionary Codebook

11/21/2020 1:43pm

#	Variable / Field Name	Field Label<br>*Field Note*	Field Attributes (Field Type, Validation, Choices, Calculations, etc.)
Instrument: **Inclusao** (inclusao)			
1	record_id	Record ID	text
2	entrevistador_id	Section Header: *CRITÉRIOS DE ELEGIBILIDADE*<br>Identificação do entrevistador:	text<br>Field Annotation: @READONLY @USERNAME @NOMISSING
3	dt_insercao	Data da inserção dos dados	text (date_dmy)<br>Field Annotation: @HIDDEN @NOW-SERVER @READONLY @NOMISSING
4	dt_coleta	Data da coleta	text (date_dmy), Required<br>Field Annotation: @NOMISSING
5	caso_contr	Participante da pesquisa<br>*Casos: Pacientes com doença reumática e com COVID-19 (segundo Ministério da Saúde) - Suspeito com exame positivo (PCR, teste rápido ou sorologia) OU - Contato com caso de COVID-19 confirmado e sintomas sugestivos nos 14 dias após o contato, sem exame confirmatório. Controle: Pacientes com doença reumática e sem COVID-19*	radio, Required<br>1 Caso (com COVID-19)<br>2 Controle (sem COVID-19)<br>Custom alignment: LV<br>Field Annotation: @NOMISSING
6	entrevista<br>Show the field ONLY if:<br>[caso_contr]='1' or [caso_contr]='2'	Qual a forma de entrevista para a inclusão do participante de pesquisa?	radio<br>1 Contato telefônico<br>2 Presencialmente<br>Custom alignment: LV<br>Field Annotation: @NOMISSING
7	info_tel<br>Show the field ONLY if:<br>[entrevista]='1'	O participante de pesquisa deve fornecer o consentimento verbal para a continuação do questionário.<br><br>Entrevistador, você deve explicar quais serão os procedimentos do estudo:<br>1) Após o contato telefônico inicial o paciente deverá comparecer ao hospital, assim que o distanciamento social termine, para uma coleta de sangue onde será realizada a sorologia da SARS-CoV-2.<br>2) As visitas subsequentes após a coleta dos dados serão realizadas com 3 meses (Visita 1), 6 meses (Visita 2), no próprio ambulatório onde ocorre o acompanhamento dos pacientes. 3) Caso aceite participar, o paciente deverá assinar o TCLE na primeira visita presencial após a inclusão.	descriptive<br>Field Annotation: @NOMISSING
8	concorda_tel_sn<br>Show the field ONLY if:<br>[entrevista]='1'	O participante de pesquisa concorda em fornecer os dados via contato telefônico?	radio<br>1 Sim<br>0 Não<br>Custom alignment: LV<br>Field Annotation: @NOMISSING
9	concorda_dt<br>Show the field ONLY if:<br>[concorda_tel_sn]='1'	Data do aceite	text (date_dmy)<br>Field Annotation: @NOMISSING

10	info_entrev<br>Show the field ONLY if:<br>[entrevista]='2'	Para continuar o preenchimento deste formulário, é obrigatório que o participante de pesquisa tenha lido e assinado o Termo de Consentimento Livre e Esclarecido (impresso) confirmando seu aceite para ser incluído no estudo.	descriptive<br>Field Annotation: @NOMISSING
11	concorda_sn_tcle<br>Show the field ONLY if:<br>[entrevista]='2'	O participante de pesquisa assinou o TCLE?<br>*TCLE: Termo de Consentimento Livre e Esclarecido*	radio<br>1 Sim<br>0 Não<br>Custom alignment: LV<br>Field Annotation: @NOMISSING
12	concorda_sn_tcle_versao<br>Show the field ONLY if:<br>[concorda_sn_tcle]='1'	Qual a versão do TCLE ?<br>*Informação disponível no rodapé do TCLE.*	checkbox<br>1 concorda_sn_tcle_versao___1 Versão 1<br>2 concorda_sn_tcle_versao___2 Versão 2<br>Custom alignment: LV<br>Field Annotation: @NOMISSING
13	concorda_dt_tcle<br>Show the field ONLY if:<br>[concorda_sn_tcle_versao(1)]='1'	Data da assinatura do TCLE - versão 1	text (date_dmy)<br>Field Annotation: @NOMISSING
14	concorda_dt_tcle_2<br>Show the field ONLY if:<br>[concorda_sn_tcle_versao(2)]='1'	Data da assinatura do TCLE - versão 2	text (date_dmy)<br>Field Annotation: @NOMISSING
15	info_inclus<br>Show the field ONLY if:<br>[concorda_tel_sn]='1' or [concorda_sn_tcle]='1'	Critérios de inclusão	descriptive<br>Field Annotation: @NOMISSING
16	eleg_idade<br>Show the field ONLY if:<br>[concorda_tel_sn]='1' or [concorda_sn_tcle]='1'	Idade maior ou igual a 18 anos ?	radio<br>1 Sim<br>0 Não<br>Custom alignment: LV<br>Field Annotation: @NOMISSING
17	eleg_reumato<br>Show the field ONLY if:<br>[eleg_idade]='1'	Diagnóstico de doença reumática imunomediada?	radio<br>1 Sim<br>0 Não<br>Custom alignment: LV<br>Field Annotation: @NOMISSING
18	eleg_covid_s<br>Show the field ONLY if:<br>[caso_contr]='1' and [eleg_reumato]='1'	Suspeita (atual ou passada) de COVID-19 segundo protocolo do Ministério da Saúde do Brasil ?<br>*Orientação MS: sintomas iniciados após 25/02/2020*	radio<br>1 Sim<br>0 Não<br>Custom alignment: LV<br>Field Annotation: @NOMISSING
19	eleg_covid_n<br>Show the field ONLY if:<br>[caso_contr]='2' and [eleg_reumato]='1'	Não ter apresentado nenhum sintoma sugestivo (atual ou passada) de COVID-19 segundo protocolo do Ministério da Saúde do Brasil ?<br>*Orientação MS: sintomas iniciados após 25/02/2020*	radio<br>1 Sim<br>0 Não<br>Custom alignment: LV<br>Field Annotation: @NOMISSING
20	info_excl<br>Show the field ONLY if:<br>[eleg_covid_s]='1' or [eleg_covid_n]='1'	Critérios de exclusão	descriptive<br>Field Annotation: @NOMISSING

21	eleg_aids<br><br>Show the field ONLY if:<br>[eleg_covid_s]='1' or [eleg_covid_n]='1'	Apresenta diagnóstico confirmado de AIDS/HIV positivo ?	radio<br>1 Sim<br>0 Não<br><br>Custom alignment: LV<br>Field Annotation: @NOMISSING
22	eleg_transp<br><br>Show the field ONLY if:<br>[eleg_aids]='0'	Realizou transplante de órgãos ou medula?<br>*Considere transplante de órgãos ou medula realizado a qualquer momento.*	radio<br>1 Sim<br>0 Não<br><br>Custom alignment: LV<br>Field Annotation: @NOMISSING
23	eleg_imuno<br><br>Show the field ONLY if:<br>[eleg_transp]='0'	Apresenta diagnóstico confirmado de imunodeficiência primária ?	radio<br>1 Sim<br>0 Não<br><br>Custom alignment: LV<br>Field Annotation: @NOMISSING
24	eleg_neopla<br><br>Show the field ONLY if:<br>[eleg_imuno]='0'	Foi diagnosticado com neoplasia (exceto câncer de pele) últimos 5 anos ?	radio<br>1 Sim<br>0 Não<br><br>Custom alignment: LV<br>Field Annotation: @NOMISSING
25	eleg_timo<br><br>Show the field ONLY if:<br>[eleg_neopla]='0'	Apresenta história pregressa de doenças do timo?<br>*Casos de ausência de timo Miastenia gravis Remoção cirúrgica Timoma*	radio<br>1 Sim<br>0 Não<br><br>Custom alignment: LV<br>Field Annotation: @NOMISSING
26	eleg_info<br><br>Show the field ONLY if:<br>[concorda_tel_sn]='0' or [concorda_sn_tcle]='0' or [eleg_idade]='0' or [eleg_reumato]='0' or [eleg_covid_s]='0' or [eleg_covid_n]='0' or [eleg_aids]='1' or [eleg_transp]='1' or [eleg_imuno]='1' or [eleg_neopla]='1' or [eleg_timo]='1'	Participante não atende aos critérios de elegibilidade do estudo.	descriptive<br>Field Annotation: @NOMISSING
27	nome<br><br>Show the field ONLY if:<br>[eleg_timo]='0'	Section Header: *DADOS DEMOGRÁFICOS*<br>Nome	text, Identifier<br>Field Annotation: @NOMISSING
28	dt_nascim<br><br>Show the field ONLY if:<br>[eleg_timo]='0'	Data de nascimento	text (date_dmy), Required<br>Field Annotation: @NOMISSING
29	idade<br><br>Show the field ONLY if:<br>[eleg_timo]='0'	Idade	calc<br>Calculation: rounddown(datediff ([dt_nascim], [dt_coleta], "y", "dmy"))<br>Field Annotation: @NOMISSING
30	telefone<br><br>Show the field ONLY if:<br>[eleg_timo]='0' and [idade]!='' and [idade]>17	Telefone com DDD	text, Identifier<br>Field Annotation: @NOMISSING
31	municipio<br><br>Show the field ONLY if:<br>[eleg_timo]='0' and [idade]!='' and [idade]>'17'	Cidade:	text<br>Field Annotation: @NOMISSING

32	uf<br><br>Show the field ONLY if:<br>[eleg_timo]='0' and [idade]!='' and [idade]>17	UF<br>*Digite as primeiras letras para auto-completar.*	dropdown (autocomplete)<br>1 Acre (AC)<br>2 Alagoas (AL)<br>3 Amapá (AP)<br>4 Amazonas (AM)<br>5 Bahia (BA)<br>6 Ceará (CE)<br>7 Distrito Federal (DF)<br>8 Espírito Santo (ES)<br>9 Goiás (GO)<br>10 Maranhão (MA)<br>11 Mato Grosso (MT)<br>12 Mato Grosso do Sul (MS)<br>13 Minas Gerais (MG)<br>14 Pará (PA)<br>15 Paraíba (PB)<br>16 Paraná (PR)<br>17 Pernambuco (PE)<br>18 Piauí (PI)<br>19 Rio de Janeiro (RJ)<br>20 Rio Grande do Norte (RN)<br>21 Rio Grande do Sul (RS)<br>22 Rondônia (RO)<br>23 Roraima (RR)<br>24 Santa Catarina (SC)<br>25 São Paulo (SP)<br>26 Sergipe (SE)<br>27 Tocantins (TO)<br><br>Field Annotation: @NOMISSING
33	sexo<br><br>Show the field ONLY if:<br>[eleg_timo]='0' and [idade]!='' and [idade]>17	Sexo	radio, Required<br>1 Masculino<br>2 Feminino<br><br>Custom alignment: LV<br>Field Annotation: @NOMISSING
34	cor_pele<br><br>Show the field ONLY if:<br>[eleg_timo]='0' and [idade]!='' and [idade]>'17'	Cor ou raça<br>*Fonte: IBGE (2020)*	radio, Required<br>0 Branca<br>1 Preta<br>2 Parda<br>3 Indígena<br>4 Amarela<br>777 Outra<br><br>Custom alignment: LV<br>Field Annotation: @NOMISSING
35	cor_pele_out<br><br>Show the field ONLY if:<br>[cor_pele]='777'	Outra cor da pele	text<br>Field Annotation: @NOMISSING

36	escolaridade<br><br>Show the field ONLY if:<br>[eleg_timo]='0' and [idade]!='' and [idade]>'17'	Escolaridade	radio<br>1 Analfabeto<br>2 Fundamental Incompleto<br>3 Fundamental Completo<br>4 Médio Incompleto<br>5 Médio Completo<br>6 Superior Incompleto<br>7 Superior Completo<br>8 Pós graduação<br><br>Custom alignment: LV<br>Field Annotation: @NOMISSING
37	profissao<br><br>Show the field ONLY if:<br>[eleg_timo]='0' and [idade]!='' and [idade]>'17'	Profissão	radio<br>1 Atendimento ao público<br>2 Saúde<br>3 Segurança<br>4 Educação<br>5 Dona de Casa<br>777 Outra<br><br>Custom alignment: LV<br>Field Annotation: @NOMISSING
38	profissao_out<br><br>Show the field ONLY if:<br>[profissao]='777'	Outra profissão	text<br>Field Annotation: @NOMISSING
39	situa_trab<br><br>Show the field ONLY if:<br>[eleg_timo]='0' and [idade]!='' and [idade]>'17'	Situação de trabalho<br>*Considere como ativo o participante de pesquisa que está empregado ou trabalhador informal, mesmo que esteja em distanciamento social, sem trabalhar presencialmente*	radio<br>1 Ativo<br>0 Inativo<br><br>Custom alignment: LV<br>Field Annotation: @NOMISSING
40	situa_trab_invativo<br><br>Show the field ONLY if:<br>[situa_trab]='0' and [idade]!='' and [idade]>'17'	Situação de trabalho inativo	radio<br>1 Aposentado<br>2 Afastado pelo INSS<br>3 Desempregado<br>4 Estudante<br>777 Outro<br><br>Custom alignment: LV<br>Field Annotation: @NOMISSING
41	situa_trab_invativo_out<br><br>Show the field ONLY if:<br>[situa_trab_invativo]='777'	Qual outra situação de trabalho inativo?	text<br>Field Annotation: @NOMISSING
42	distanc<br><br>Show the field ONLY if:<br>[eleg_timo]='0' and [idade]!='' and [idade]>'17'	Todas as pessoas que moram na sua casa, incluindo você, permaneceram em distanciamento social durante a epidemia de COVID-19?<br>*Distanciamento social: afastamento de suas atividades profissionais e obrigações familiares e de lazer para reduzir a transmissão, por vontade própria, orientação da empresa onde trabalha ou decreto governamental para serviços não essenciais), saindo apenas para realizar coisas estritamente necessárias, como comprar comida ou ir a farmácia*	radio, Required<br>1 Sim<br>0 Não<br><br>Custom alignment: LV<br>Field Annotation: @NOMISSING

43	comorb<br><br>Show the field ONLY if:<br>[eleg_timo]='0' and [idade]!='' and [idade]>'17'	Section Header: *ANTECEDENTES PESSOAIS*<br><br>Comorbidades	checkbox, Required<br>1 — comorb___1 — Cardiopatia<br>2 — comorb___2 — Diabetes mellitus<br>3 — comorb___3 — Doença pulmonar (doença intersticial pulmonar, asma, DPOC)<br>4 — comorb___4 — Doença renal<br>5 — comorb___5 — Hipertensão arterial sistêmica<br>6 — comorb___6 — Obesidade<br>777 — comorb___777 — Outras<br>0 — comorb___0 — Nenhuma comorbidade<br><br>Custom alignment: LV<br>Field Annotation: @NOMISSING @NONEOFTHEABOVE='0'
44	outras_comorb<br><br>Show the field ONLY if:<br>[comorb(777)]='1'	Qual(is) outra(s) comorbidades<br>*Em caso de mais de uma, descrever uma por linha*	notes<br>Custom alignment: LV<br>Field Annotation: @NOMISSING
45	tabagismo<br><br>Show the field ONLY if:<br>[eleg_timo]='0' and [idade]!='' and [idade]>'17'	Tabagismo<br>*Considerar SIM se fuma mais que 1 cigarro por dia TODOS os dias*	radio<br>1 — Sim<br>0 — Não<br><br>Custom alignment: LV<br>Field Annotation: @NOMISSING
46	etilismo<br><br>Show the field ONLY if:<br>[eleg_timo]='0' and [idade]!='' and [idade]>'17'	Etilismo<br>*Considerar etilismo se: 15 doses por semana para os homens e 10 doses/semana para mulheres. Uma dose equivale a aproximadamente 285 ml de cerveja, 120 ml de vinho e 30 ml de destilado(whisky, vodka e pinga)*	radio<br>1 — Sim<br>0 — Não<br><br>Custom alignment: LV<br>Field Annotation: @NOMISSING
47	peso_referido<br><br>Show the field ONLY if:<br>[eleg_timo]='0' and [idade]!='' and [idade]>'17'	Peso referido (Kg)<br>*Kg Apenas números, separar decimais por ponto (.)*	text (number)
48	info_covid<br><br>Show the field ONLY if:<br>[caso_contr]='1' and [eleg_timo]='0'	Informações sobre os sintomas de COVID-19	descriptive

<table>
<tr><td>49</td><td>sintoma_s

Show the field ONLY if:
[caso_contr]='1' and [eleg_tim o]='0' and [idade]!='' and [idad e]>'17'</td><td>Sintomas apresentados</td><td>checkbox, Required<table>
<tr><td>0</td><td>sintoma_s___0</td><td>Assintomático</td></tr>
<tr><td>15</td><td>sintoma_s___15</td><td>Alterações de pele</td></tr>
<tr><td>1</td><td>sintoma_s___1</td><td>Artralgias</td></tr>
<tr><td>2</td><td>sintoma_s___2</td><td>Astenia</td></tr>
<tr><td>3</td><td>sintoma_s___3</td><td>Cefaleia</td></tr>
<tr><td>4</td><td>sintoma_s___4</td><td>Coriza</td></tr>
<tr><td>5</td><td>sintoma_s___5</td><td>Diarreia</td></tr>
<tr><td>6</td><td>sintoma_s___6</td><td>Dispneia</td></tr>
<tr><td>7</td><td>sintoma_s___7</td><td>Febre</td></tr>
<tr><td>8</td><td>sintoma_s___8</td><td>Mialgias</td></tr>
<tr><td>9</td><td>sintoma_s___9</td><td>Náuseas</td></tr>
<tr><td>10</td><td>sintoma_s___10</td><td>Perda de Olfato</td></tr>
<tr><td>11</td><td>sintoma_s___11</td><td>Perda de paladar</td></tr>
<tr><td>12</td><td>sintoma_s___12</td><td>Tonturas</td></tr>
<tr><td>13</td><td>sintoma_s___13</td><td>Tosse</td></tr>
<tr><td>14</td><td>sintoma_s___14</td><td>Vômitos</td></tr>
<tr><td>777</td><td>sintoma_s___777</td><td>Outros</td></tr>
</table>Custom alignment: LV
Field Annotation: @NOMISSING @NONEOFTHEABOVE='0'</td></tr>
<tr><td>50</td><td>sintoma_s_pele

Show the field ONLY if:
[sintoma_s(15)]='1'</td><td>Qual alteração de pele? Descreva:</td><td>notes
Field Annotation: @NOMISSING</td></tr>
<tr><td>51</td><td>sintoma_s_out

Show the field ONLY if:
[sintoma_s(777)]='1'</td><td>Qual outro sintoma não listado? Descreva:</td><td>notes
Field Annotation: @NOMISSING</td></tr>
<tr><td>52</td><td>sintoma_dt

Show the field ONLY if:
[caso_contr]='1' and [eleg_tim o]='0' and [sintoma_s(0)]<>'1' a nd [idade]!='' and [idade]>'17'</td><td>Data de início dos sintomas:
Padrão Internacional para incerteza de data:"Meio do meio" Exemplo: Insira 15 se existe dúvida quanto ao dia do mês; Insira Junho (15/6/2019) se existe dúvida quanto ao mês do ano.</td><td>text (date_dmy), Required
Field Annotation: @NOMISSING</td></tr>
<tr><td>53</td><td>sintoma_ainda

Show the field ONLY if:
[caso_contr]='1' and [eleg_tim o]='0' and [sintoma_s(0)]<>'1' a nd [idade]!='' and [idade]>'17'</td><td>Ainda apresentando sintomas de COVID-19 ?</td><td>radio<table>
<tr><td>1</td><td>Sim</td></tr>
<tr><td>0</td><td>Não</td></tr>
</table>Custom alignment: LV
Field Annotation: @NOMISSING</td></tr>
<tr><td>54</td><td>sintoma_durac

Show the field ONLY if:
[sintoma_ainda]='0'</td><td>Tempo de duração dos sintomas
Em dias. Apenas números.</td><td>text (integer)
Field Annotation: @NOMISSING</td></tr>
<tr><td>55</td><td>contato

Show the field ONLY if:
[caso_contr]='1' and [eleg_tim o]='0' and [idade]!='' and [idad e]>'17'</td><td>Teve contato com algum caso confirmado de infecção pelo COVID-19?</td><td>radio<table>
<tr><td>1</td><td>Sim</td></tr>
<tr><td>0</td><td>Não</td></tr>
<tr><td>99</td><td>Não sabe</td></tr>
</table>Custom alignment: LV
Field Annotation: @NOMISSING</td></tr>
</table>

56	contato_local<br>Show the field ONLY if: [contato]='1' and [idade]!='' and [idade]>'17'	Onde ocorreu esse contato?	radio<br>1 Em casa<br>2 No trabalho<br>3 Transporte público<br>4 Viagem para área endémica<br>777 Outro ambiente (detalhar abaixo)<br>Custom alignment: LV<br>Field Annotation: @NOMISSING
57	contato_local_out<br>Show the field ONLY if: [contato_local]='777'	Qual outro local (descrever)?	text<br>Field Annotation: @NOMISSING
58	contato_caso<br>Show the field ONLY if: [caso_contr]='2' and [eleg_timo]='0' and [idade]!='' and [idade]>'17'	Teve contato com algum caso confirmado de infecção pelo COVID-19?	radio<br>1 Sim<br>0 Não<br>99 Não sabe<br>Custom alignment: LV<br>Field Annotation: @NOMISSING
59	contato_local_caso<br>Show the field ONLY if: [contato_caso]='1' and [idade]!='' and [idade]>'17'	Onde ocorreu esse contato?	radio<br>1 Em casa<br>2 No trabalho<br>3 Transporte público<br>4 Viagem para área endémica<br>777 Outro ambiente (detalhar abaixo)<br>Custom alignment: LV<br>Field Annotation: @NOMISSING
60	contato_local_out_2<br>Show the field ONLY if: [contato_local_caso]='777'	Qual outro local (descrever)?	text<br>Field Annotation: @NOMISSING
61	diagnostico<br>Show the field ONLY if: [caso_contr]='1' and [eleg_timo]='0' and [idade]!='' and [idade]>'17'	O diagnóstico de COVID-19 foi confirmado por exame laboratorial?	radio<br>1 Sim<br>0 Não<br>Custom alignment: LV<br>Field Annotation: @NOMISSING
62	diagnostico_tipo<br>Show the field ONLY if: [diagnostico]='1'	Que tipo de exame foi feito para confirmar o diagnóstico de COVID-19?	checkbox, Required<br>1 diagnostico_tipo___1 RT-PCR (swab de nariz e garganta)<br>2 diagnostico_tipo___2 Sorologia para SARS-CoV-2<br>3 diagnostico_tipo___3 Teste rápido<br>0 diagnostico_tipo___0 Não sabe ou não lembra<br>Custom alignment: LV<br>Field Annotation: @NOMISSING @NONEOFTHEABOVE ='0'
63	diagnostico_dt_exame<br>Show the field ONLY if: [diagnostico_tipo(1)]='1'	Qual a data do exame RT-PCR	text (date_dmy)<br>Field Annotation: @NOMISSING
64	diagnostico_soro<br>Show the field ONLY if: [diagnostico_tipo(2)]='1'	Sorologia para SARS-CoV-2	checkbox<br>1 diagnostico_soro___1 IgM<br>2 diagnostico_soro___2 IgG<br>Custom alignment: LV<br>Field Annotation: @NOMISSING

65	diagnostico_dt_soro_igm<br><br>Show the field ONLY if:<br>[diagnostico_soro(1)]='1'	Qual a data do exame sorologia IgM	text (date_dmy)<br>Field Annotation: @NOMISSING
66	diagnostico_dt_soro_igm_2<br><br>Show the field ONLY if:<br>[diagnostico_soro(2)]='1'	Qual a data do exame sorologia IgG	text (date_dmy)<br>Field Annotation: @NOMISSING
67	diagnostico_dt_teste_rapido<br><br>Show the field ONLY if:<br>[diagnostico_tipo(3)]='1'	Qual a data do teste rápido	text (date_dmy)<br>Field Annotation: @NOMISSING
68	atend_telemedicina<br><br>Show the field ONLY if:<br>[caso_contr]='1' and [eleg_timo]='0' and [idade]!='' and [idade]>'17'	O participante de pesquisa recebeu atendimento por telemedicina?<br>*Atendimento por telemedicina: orientação por profissional de saúde - via contato telefônico ou internet - quanto aos sinais e sintomas e a necessidade, ou não, de procurar o serviço de saúde.*	radio<br>1 Sim<br>0 Não<br><br>Custom alignment: LV<br>Field Annotation: @NOMISSING
69	atend_ambulatorio<br><br>Show the field ONLY if:<br>[caso_contr]='1' and [eleg_timo]='0' and [idade]!='' and [idade]>'17'	O participante de pesquisa recebeu atendimento no ambulatório/consultorio?	radio<br>1 Sim<br>0 Não<br><br>Custom alignment: LV<br>Field Annotation: @NOMISSING
70	atend_hosp<br><br>Show the field ONLY if:<br>[caso_contr]='1' and [eleg_timo]='0' and [idade]!='' and [idade]>'17'	O participante de pesquisa necessitou de atendimento hospitalar?	radio<br>1 Sim<br>0 Não<br><br>Custom alignment: LV<br>Field Annotation: @NOMISSING
71	atend_hosp_tipo<br><br>Show the field ONLY if:<br>[atend_hosp]='1'	Especifique o local de internação	checkbox, Required<br>1 atend_hosp_tipo___1 Atendido na emergência e liberado para casa<br>2 atend_hosp_tipo___2 Internamento enfermaria<br>3 atend_hosp_tipo___3 Internamento UTI<br>4 atend_hosp_tipo___4 Hospital de campanha<br>5 atend_hosp_tipo___5 Observação de até 24h<br><br>Custom alignment: LV<br>Field Annotation: @NOMISSING
72	atend_hosp_dt_ent<br><br>Show the field ONLY if:<br>[atend_hosp_tipo(2)]='1' or [atend_hosp_tipo(3)]='1' or [atend_hosp_tipo(4)]='1' or [atend_hosp_tipo(5)]='1'	Data da internação	text (date_dmy)<br>Field Annotation: @NOMISSING
73	atend_hosp_ventila<br><br>Show the field ONLY if:<br>[atend_hosp_tipo(2)]='1' or [atend_hosp_tipo(3)]='1' or [atend_hosp_tipo(4)]='1' or [atend_hosp_tipo(5)]='1'	Precisou de ventilação mecânica?	radio, Required<br>1 Sim<br>0 Não<br><br>Custom alignment: LV<br>Field Annotation: @NOMISSING
74	atend_hosp_evol<br><br>Show the field ONLY if:<br>[atend_hosp_tipo(2)]='1' or [atend_hosp_tipo(3)]='1' or [atend_hosp_tipo(4)]='1' or [atend_hosp_tipo(5)]='1'	Evolução do internamento?	radio, Required<br>0 Ainda permanece internado<br>1 Alta vivo<br>2 Óbito<br><br>Custom alignment: LV<br>Field Annotation: @NOMISSING

<table>
<tr><td>75</td><td>atend_hosp_evol_dt

Show the field ONLY if:
[atend_hosp_evol]='1' or [atend_hosp_evol]='2'</td><td>Indique a data [atend_hosp_evol]</td><td>text (date_dmy), Required
Field Annotation: @NOMISSING</td></tr>
<tr><td>76</td><td>inclusao_tomo_referido

Show the field ONLY if:
[entrevista]='1' and [caso_contr]='1' and [eleg_timo]='0' and [idade]!='' and [idade]>'17'</td><td>Realizou tomografia computadorizada de tórax ?</td><td>radio, Required<table><tr><td>1</td><td>Sim</td></tr><tr><td>0</td><td>Não</td></tr><tr><td>999</td><td>Não sabe</td></tr></table>Custom alignment: LV</td></tr>
<tr><td>77</td><td>inclusao_tomo_dt_referido

Show the field ONLY if:
[inclusao_tomo_referido]='1'</td><td>Data da tomografia de tórax</td><td>text (date_dmy)</td></tr>
<tr><td>78</td><td>inclusao_tomo_laudo_referido

Show the field ONLY if:
[inclusao_tomo_referido]='1'</td><td>Laudo disponível?
IMPORTANTE: Orientar que o paciente leve a tomografia na Visita Presencial.</td><td>radio<table><tr><td>1</td><td>Sim</td></tr><tr><td>0</td><td>Não</td></tr></table>Custom alignment: LV</td></tr>
<tr><td>79</td><td>inclusao_tomo_s_referido

Show the field ONLY if:
[inclusao_tomo_laudo_referido]='1'</td><td>Laudo da tomografia de tórax</td><td>radio<table><tr><td>0</td><td>Normal</td></tr><tr><td>1</td><td>Alterada</td></tr></table>Custom alignment: LV</td></tr>
<tr><td>80</td><td>medicam_covid

Show the field ONLY if:
[caso_contr]='1' and [eleg_timo]='0' and [idade]!='' and [idade]>'17'</td><td>Medicações utilizadas para tratamento da COVID-19</td><td>checkbox, Required<table>
<tr><td>27</td><td>medicam_covid___27</td><td>Analgésicos</td></tr>
<tr><td>21</td><td>medicam_covid___21</td><td>Azitromicina</td></tr>
<tr><td>9</td><td>medicam_covid___9</td><td>Corticoide oral</td></tr>
<tr><td>32</td><td>medicam_covid___32</td><td>Heparina</td></tr>
<tr><td>28</td><td>medicam_covid___28</td><td>Hidroxicloroquina/Difosfato de Cloroquina</td></tr>
<tr><td>23</td><td>medicam_covid___23</td><td>Imunoglobulina EV</td></tr>
<tr><td>24</td><td>medicam_covid___24</td><td>Inibidor de JAK</td></tr>
<tr><td>29</td><td>medicam_covid___29</td><td>Oxigenação de membrana extra-corpórea (ECMO)</td></tr>
<tr><td>31</td><td>medicam_covid___31</td><td>Plasmaférese</td></tr>
<tr><td>25</td><td>medicam_covid___25</td><td>Pulso com dexametasona</td></tr>
<tr><td>26</td><td>medicam_covid___26</td><td>Pulso com metilprednisolona</td></tr>
<tr><td>30</td><td>medicam_covid___30</td><td>Soro/Plasma de convalescente</td></tr>
<tr><td>18</td><td>medicam_covid___18</td><td>Tocilizumabe</td></tr>
<tr><td>999</td><td>medicam_covid___999</td><td>Não sabe ou não respondeu</td></tr>
<tr><td>0</td><td>medicam_covid___0</td><td>Nenhuma medicação</td></tr>
<tr><td>777</td><td>medicam_covid___777</td><td>Outros (detalhar abaixo)</td></tr>
</table>Custom alignment: LV
Field Annotation: @NOMISSING @NONEOFTHEABOVE='0,999'</td></tr>
<tr><td>81</td><td>medicam_covid_out

Show the field ONLY if:
[medicam_covid(777)]='1'</td><td>Medicações utilizadas para tratamento da COVID-19</td><td>notes
Custom alignment: LV
Field Annotation: @NOMISSING</td></tr>
</table>

82	medicam_covid_corticoide<br><br>Show the field ONLY if:<br>[medicam_covid(9)]='1'	Selecione a dose do corticoide oral:	radio<br>1 Até 10 mg/dia<br>2 >=11 a 20 mg/dia<br>3 > 20 mg/dia<br><br>Custom alignment: LV<br>Field Annotation: @NOMISSING
83	medicam_covid_heparina<br><br>Show the field ONLY if:<br>[medicam_covid(32)]='1'	Selecione a dose da heparina:	radio<br>1 Dose plena<br>2 Dose profilática<br><br>Custom alignment: LV<br>Field Annotation: @NOMISSING
84	trat_experiment<br><br>Show the field ONLY if:<br>[caso_contr]='1' and [eleg_timo]='0' and [idade]!='' and [idade]>'17'	Durante a internação da infecção pelo novo coronavírus, você recebeu algum tratamento experimental ?	radio<br>1 Sim<br>0 Não<br>999 Não disponível<br><br>Custom alignment: LV<br>Field Annotation: @NOMISSING
85	trat_experiment_s<br><br>Show the field ONLY if:<br>[trat_experiment]='1'	Qual dos abaixo?	radio<br>1 Oxigenação por membrana extra-corpórea<br>2 Plasmaférese<br>3 Soro de convalescente<br><br>Custom alignment: LV<br>Field Annotation: @NOMISSING
86	info_doen_reuma<br><br>Show the field ONLY if:<br>[eleg_timo]='0'	DIAGNÓSTICO DA DOENÇA REUMÁTICA	descriptive
87	doen_reuma<br><br>Show the field ONLY if:<br>[eleg_timo]='0' and [idade]!='' and [idade]>'17'	Diagnóstico da doença reumática do [caso_contr]	radio, Required<br>1 Artrite enteropática<br>2 Artrite idiopática juvenil (início antes do 16 anos) na idade adulta<br>3 Artrite psoriásica<br>4 Artrite reumatoide<br>5 Doença de Behçet<br>6 Doença de Still do adulto<br>7 Doença mista do tecido conjuntivo<br>8 Esclerose sistêmica<br>9 Espondilite anquilosante/espondiloartrite axial<br>10 Lupus eritematoso sistêmico<br>11 Miopatia inflamatória (Dermatomiosite)<br>12 Sarcoidose<br>13 Síndrome de Sjögren<br>14 Síndrome de sobreposição<br>15 Síndrome do anticorpo antifosfolípide primária<br>16 Vasculites<br><br>Custom alignment: LV<br>Field Annotation: @NOMISSING
88	doen_reuma_tempo<br><br>Show the field ONLY if:<br>[doen_reuma]<>''	Tempo de diagnóstico da doença reumática [doen_reuma] será em:	radio, Required<br>1 Meses<br>2 Anos<br><br>Custom alignment: LV<br>Field Annotation: @NOMISSING

89	doen_reuma_tempo_mes<br>Show the field ONLY if:<br>[doen_reuma_tempo]='1'	Tempo de diagnóstico da doença reumática: [doen_reuma]<br>*Em meses. Apenas números.*	text (integer), Required<br>Field Annotation: @NOMISSING
90	doen_reuma_tempo_ano<br>Show the field ONLY if:<br>[doen_reuma_tempo]='2'	Tempo de diagnóstico da doença reumática: [doen_reuma]<br>*Em anos. (total de anos de diagnóstico da doença) Apenas números.*	text (integer), Required<br>Field Annotation: @NOMISSING
91	info_tratamento<br>Show the field ONLY if:<br>[eleg_timo]='0'	TRATAMENTO DA DOENÇA REUMÁTICA	descriptive<br>Field Annotation: @NOMISSING
92	medicam<br>Show the field ONLY if:<br>[eleg_timo]='0' and [idade]!='' and [idade]>'17'	Faz uso de algum medicamento da lista abaixo? (Considere os últimos 30 dias, exceções para os medicamentos que possuem intervalo de tomada superior a 30 dias)	checkbox, Required<br>1 | medicam___1 | Abatacepte<br>2 | medicam___2 | Anti-Il-17 (secuquinumabe, ixezequizumabe)<br>3 | medicam___3 | Anti-IL12/23 (ustequinumabe - considere uso regular)<br>4 | medicam___4 | Anti-TNF (infliximabe - considere uso regular , etanercepte, adalimumabe, golimumabe, certolizumabe)<br>5 | medicam___5 | Azatioprina<br>6 | medicam___6 | Belimumabe<br>7 | medicam___7 | Ciclofosfamida oral<br>8 | medicam___8 | Ciclosporina<br>9 | medicam___9 | Corticoide oral<br>10 | medicam___10 | Hidroxicloroquina/Difosfato de cloroquina<br>11 | medicam___11 | Inibidor de JAK (tofacitinibe, baricitinibe, upadacitinibe)<br>12 | medicam___12 | Leflunomida<br>13 | medicam___13 | Metotrexato<br>14 | medicam___14 | MMF (Microfenolato de Mofetil)<br>15 | medicam___15 | Pulsoterapia com ciclofosfamida<br>16 | medicam___16 | Pulsoterapia com metiprednisolona<br>17 | medicam___17 | Rituximabe (considere uso regular)<br>18 | medicam___18 | Sulfassalazina<br>19 | medicam___19 | Tocilizumabe<br>777 | medicam___777 | Outros (detalhar abaixo)<br>999 | medicam___999 | Não sabe ou não respondeu<br>0 | medicam___0 | Nenhuma medicação<br>Custom alignment: LV<br>Field Annotation: @NOMISSING @NONEOFTHEABOVE='999,0'
93	outro_med_assoc<br>Show the field ONLY if:<br>[medicam(777)] = '1'	Outros medicamentos:<br>*Em caso de mais de um, descrever um por linha*	notes<br>Field Annotation: @NOMISSING

94	dose_cort_oral<br><br>Show the field ONLY if:<br>[medicam(9)] = '1'	Dose corticoide oral	radio, Required<br>1 | até 10 mg/dia<br>2 | >= 11 a 20 mg/dia<br>3 | >= 21 mg/dia<br>99 | Não sabe a dose em uso<br><br>Custom alignment: LV<br>Field Annotation: @NONEOFTHEABOVE='99' @NOMISSING
95	dose_mtx<br><br>Show the field ONLY if:<br>[medicam(13)] = '1'	Dose Metotrexato	radio, Required<br>1 | < = 20 mg/semana<br>2 | >= 21 mg/semana<br>99 | Não sabe a dose em uso<br><br>Custom alignment: LV<br>Field Annotation: @NONEOFTHEABOVE='99' @NOMISSING
96	mudanca<br><br>Show the field ONLY if:<br>[caso_contr]='1' and [eleg_timo]='0' and [idade]!='' and [idade]>'17'	Houve mudança do tratamento após o início dos sintomas?	radio, Required<br>1 | Sim<br>0 | Não<br><br>Custom alignment: LV<br>Field Annotation: @NOMISSING
97	mudanca_s<br><br>Show the field ONLY if:<br>[mudanca]='1'	Indique qual a mudança:	checkbox<br>1 | mudanca_s___1 | Dose medicamento<br>2 | mudanca_s___2 | Suspensão do medicamento<br>3 | mudanca_s___3 | Esquema terapêutico<br>777 | mudanca_s___777 | Outra mudança (detalhar abaixo)<br><br>Custom alignment: LV<br>Field Annotation: @NOMISSING
98	mudanca_out<br><br>Show the field ONLY if:<br>[mudanca_s(777)] = '1'	Outra mudança no tratamento<br>*Em caso de mais de uma, descrever uma por linha*	notes<br>Custom alignment: LV<br>Field Annotation: @NOMISSING

99	mudanca_s_retirada<br><br>Show the field ONLY if:<br>[mudanca_s(2)] = '1' or [mudanca_s(3)] = '1'	Selecione os medicamentos que foram RETIRADOS do esquema terapêutico?	checkbox<br>(choices below)<br><br>Custom alignment: LV<br>Field Annotation: @NOMISSING @NONEOFTHEABOVE='999,0'
100	mudanca_s_retirada_out<br><br>Show the field ONLY if:<br>[mudanca_s_retirada(777)]='1'	Qual outro medicamento RETIRADO do esquema terapêutico?	text<br>Field Annotation: @NOMISSING

Choices for field 99 (checkbox):

1	mudanca_s_retirada___1	Abatacepte
2	mudanca_s_retirada___2	Anti-Il-17 (secuquinumal ixezequizumabe)
3	mudanca_s_retirada___3	Anti-IL12/23 (ustequinumabe)
4	mudanca_s_retirada___4	Anti-TNF (infliximabe, etanercepte, adalimuma golimumabe, certolizumabe)
5	mudanca_s_retirada___5	Azatioprina
6	mudanca_s_retirada___6	Belimumabe
7	mudanca_s_retirada___7	Ciclofosfamida oral
8	mudanca_s_retirada___8	Ciclosporina
9	mudanca_s_retirada___9	Corticoide oral
10	mudanca_s_retirada___10	Hidroxicloroquina/Difos de cloroquina
11	mudanca_s_retirada___11	Inibidor de JAK (tofacitin baricitinibe, upadacitinib
12	mudanca_s_retirada___12	Leflunomida
13	mudanca_s_retirada___13	Metotrexato
14	mudanca_s_retirada___14	MMF
15	mudanca_s_retirada___15	Pulsoterapia com ciclofosfamida
16	mudanca_s_retirada___16	Pulsoterapia com metiprednisolona
17	mudanca_s_retirada___17	Rituximabe
18	mudanca_s_retirada___18	Sulfassalazina
19	mudanca_s_retirada___19	Tocilizumabe
777	mudanca_s_retirada___777	Outros (detalhar abaixo
999	mudanca_s_retirada___999	Não sabe ou não respondeu
0	mudanca_s_retirada___0	Nenhuma medicação

<table>
<tr><td>101</td><td>mudanca_s_incluido

Show the field ONLY if:
[mudanca_s(3)] = '1'</td><td>Selecione os medicamentos que foram INCLUÍDOS no esquema terapêutico?</td><td>checkbox
<table>
<tr><td>1</td><td>mudanca_s_incluido___1</td><td>Abatacepte</td></tr>
<tr><td>2</td><td>mudanca_s_incluido___2</td><td>Anti-Il-17 (secuquinuma ixezequizumabe)</td></tr>
<tr><td>3</td><td>mudanca_s_incluido___3</td><td>Anti-IL12/23 (ustequinumabe)</td></tr>
<tr><td>4</td><td>mudanca_s_incluido___4</td><td>Anti-TNF (infliximabe, etanercepte, adalimuma golimumabe, certolizumabe)</td></tr>
<tr><td>5</td><td>mudanca_s_incluido___5</td><td>Azatioprina</td></tr>
<tr><td>6</td><td>mudanca_s_incluido___6</td><td>Belimumabe</td></tr>
<tr><td>7</td><td>mudanca_s_incluido___7</td><td>Ciclofosfamida oral</td></tr>
<tr><td>8</td><td>mudanca_s_incluido___8</td><td>Ciclosporina</td></tr>
<tr><td>9</td><td>mudanca_s_incluido___9</td><td>Corticoide oral</td></tr>
<tr><td>10</td><td>mudanca_s_incluido___10</td><td>Hidroxicloroquina/Difos de cloroquina</td></tr>
<tr><td>11</td><td>mudanca_s_incluido___11</td><td>Inibidor de JAK (tofacitin baricitinibe, upadacitinib</td></tr>
<tr><td>12</td><td>mudanca_s_incluido___12</td><td>Leflunomida</td></tr>
<tr><td>13</td><td>mudanca_s_incluido___13</td><td>Metotrexato</td></tr>
<tr><td>14</td><td>mudanca_s_incluido___14</td><td>MMF</td></tr>
<tr><td>15</td><td>mudanca_s_incluido___15</td><td>Pulsoterapia com ciclofosfamida</td></tr>
<tr><td>16</td><td>mudanca_s_incluido___16</td><td>Pulsoterapia com metiprednisolona</td></tr>
<tr><td>17</td><td>mudanca_s_incluido___17</td><td>Rituximabe</td></tr>
<tr><td>18</td><td>mudanca_s_incluido___18</td><td>Sulfassalazina</td></tr>
<tr><td>19</td><td>mudanca_s_incluido___19</td><td>Tocilizumabe</td></tr>
<tr><td>777</td><td>mudanca_s_incluido___777</td><td>Outros (detalhar abaixo</td></tr>
<tr><td>999</td><td>mudanca_s_incluido___999</td><td>Não sabe ou não respondeu</td></tr>
<tr><td>0</td><td>mudanca_s_incluido___0</td><td>Nenhuma medicação</td></tr>
</table>
Custom alignment: LV
Field Annotation: @NOMISSING
@NONEOFTHEABOVE='999,0'</td></tr>
<tr><td>102</td><td>mudanca_s_incluido_out

Show the field ONLY if:
[mudanca_s_incluido(777)]='1'</td><td>Qual outro medicamento INCLUÍDO no esquema terapêutico?</td><td>text
Field Annotation: @NOMISSING</td></tr>
<tr><td>103</td><td>inclusao_complete</td><td>Section Header: Form Status
Complete?</td><td>dropdown
<table>
<tr><td>0</td><td>Incomplete</td></tr>
<tr><td>1</td><td>Unverified</td></tr>
<tr><td>2</td><td>Complete</td></tr>
</table></td></tr>
<tr><td colspan="4">Instrument: Aval Reumatica (aval_reumatica)</td></tr>
<tr><td>104</td><td>info_tel_tcle

Show the field ONLY if:
[entrevista]='1'</td><td>A coleta inicial foi realizada por telefone.É obrigatório que o participante de pesquisa tenha lido e assinado o Termo de Consentimento Livre e Esclarecido (impresso) confirmando seu aceite para ser incluído no estudo.</td><td>descriptive</td></tr>
<tr><td>105</td><td>concorda_sn_tcle_tel

Show the field ONLY if:
[entrevista]='1'</td><td>O participante de pesquisa assinou o TCLE?</td><td>radio, Required
<table>
<tr><td>1</td><td>Sim</td></tr>
<tr><td>0</td><td>Não</td></tr>
</table>
Custom alignment: LV
Field Annotation: @NOMISSING</td></tr>
</table>

106	info_tcle_tel<br><br>Show the field ONLY if:<br>[entrevista]='1' and [caso_contr]='1'	Se o paciente COVID19 positivo faleceu, você pode inserir os dados clínicos e laboratoriais dele e, nesse caso em especial, não será necessária a aplicação do TCLE para o responsável.	descriptive<br>Field Annotation: @NOMISSING
107	concorda_sn_tcle_tel_versao<br><br>Show the field ONLY if:<br>[concorda_sn_tcle_tel]='1'	Qual a versão do TCLE ?<br>*Informação disponível no rodapé do TCLE.*	checkbox<br>1 | concorda_sn_tcle_tel_versao___1 | Versão 1<br>2 | concorda_sn_tcle_tel_versao___2 | Versão 2<br><br>Custom alignment: LV<br>Field Annotation: @NOMISSING
108	concorda_sn_tcle_tel_dt<br><br>Show the field ONLY if:<br>[concorda_sn_tcle_tel_versao(1)]='1'	Data da assinatura do TCLE - Versão 1	text (date_dmy)<br>Field Annotation: @NOMISSING
109	concorda_sn_tcle_tel_dt_2<br><br>Show the field ONLY if:<br>[concorda_sn_tcle_tel_versao(2)]='1'	Data da assinatura do TCLE - Versão 2	text (date_dmy)<br>Field Annotation: @NOMISSING
110	aval_reum_dt<br><br>Show the field ONLY if:<br>[eleg_timo]='0'	Section Header: *AVALIAÇÃO CLÍNICA GERAL*<br>Data da avaliação	text (date_dmy), Required
111	peso<br><br>Show the field ONLY if:<br>[eleg_timo]='0'	Peso<br>*Kg Apenas números, separar decimais por ponto (.)*	text (number)
112	altura<br><br>Show the field ONLY if:<br>[eleg_timo]='0'	Altura<br>*Centímetro Apenas números (valor inteiro).*	text (integer, Max: 300)
113	imc<br><br>Show the field ONLY if:<br>[eleg_timo]='0'	IMC	calc<br>Calculation: [peso]/(([altura]/100)*([altura]/100))
114	circ_abdominal<br><br>Show the field ONLY if:<br>[eleg_timo]='0'	Circ. Abdominal:<br>*Apenas números, separar decimais por ponto (.)*	text (number)
115	pas<br><br>Show the field ONLY if:<br>[eleg_timo]='0'	PAS<br>*Apenas números (valor inteiro).*	text (integer)
116	pad<br><br>Show the field ONLY if:<br>[eleg_timo]='0'	PAD<br>*Apenas números (valor inteiro).*	text (integer)
117	asculta<br><br>Show the field ONLY if:<br>[eleg_timo]='0'	Ausculta respiratória	radio<br>1 | Estertores finos<br>2 | MV abolido<br>3 | Normal<br>4 | Roncos<br>5 | Sibilos<br>888 | Desconhecido<br><br>Custom alignment: LV<br>Field Annotation: @NOMISSING
118	outras<br><br>Show the field ONLY if:<br>[eleg_timo]='0'	Outras alterações exame físico (descreva)<br>*Descreva as alterações consideradas relevantes no exame físico.*	notes<br>Field Annotation: @NOMISSING
119	inclusao_tomo<br><br>Show the field ONLY if:<br>[caso_contr]='1' and [eleg_timo]='0'	Tomografia computadorizada de tórax disponível?	radio<br>1 | Sim<br>0 | Não<br><br>Custom alignment: LV

120	inclusao_tomo_s<br>Show the field ONLY if:<br>[inclusao_tomo]='1'	Laudo da tomografia de tórax	radio<br>0 Normal<br>1 Alterada<br>Custom alignment: LV
121	inclusao_tomo_titulo<br>Show the field ONLY if:<br>[inclusao_tomo_s]='1'	Selecione a alteração da tomografia de tórax	radio<br>1 Vidro fosco < 50%<br>2 Vidro fosco >= 50%<br>3 Condensação<br>4 Piora do padrão habitual<br>Custom alignment: LV
122	inclusao_tomo_dt<br>Show the field ONLY if:<br>[inclusao_tomo]='1'	Data da tomografia de tórax	text (date_dmy)
123	info_doen_reuma2<br>Show the field ONLY if:<br>[eleg_timo]='0'	AVALIAÇÃO DA DOENÇA REUMÁTICA	descriptive
124	info_doen_reuma_nao<br>Show the field ONLY if:<br>[doen_reuma]=''	É necessário selecionar uma doença reumática.	descriptive
125	info_antes_caso1<br>Show the field ONLY if:<br>[caso_contr]='1' and [doen_reuma]='1'	ARTRITE ENTEROPÁTICA - Antes dos sintomas da covid-19 Avaliação mais recente e disponível em prontuário, de no máximo 6 meses atrás	descriptive<br>Field Annotation: @NOMISSING
126	info_antes_contr1<br>Show the field ONLY if:<br>[caso_contr]='2' and [doen_reuma]='1'	ARTRITE ENTEROPÁTICA - Avaliação prévia Avaliação mais recente e disponível em prontuário, de no máximo 6 meses atrás	descriptive<br>Field Annotation: @NOMISSING
127	antes_grau_reuma1<br>Show the field ONLY if:<br>[doen_reuma]='1'	Qual o grau de atividade da doença?<br>*Indique um valor de 0 a 10 0 = ausência de atividade e 10= atividade intensa.*	text (integer, Min: 0, Max: 10)
128	antes_asdas_vsh1<br>Show the field ONLY if:<br>[doen_reuma]='1'	ASDAS VSH<br>*Apenas números, separar decimal com ponto (.) ASDAS VSH: Ankylosing Spondylitis Disease Activity index calculado com VSH.*	text (number)
129	antes_asdas_pcr1<br>Show the field ONLY if:<br>[doen_reuma]='1'	ASDAS PCR<br>*Apenas números, separar decimal com ponto (.) ASDAS PCR: Ankylosing Spondylitis Disease Activity index calculado com PCR.*	text (number)
130	antes_man_clinic1<br>Show the field ONLY if:<br>[doen_reuma]='1'	Assinale as manifestações clínicas em atividade	checkbox<br>1 antes_man_clinic1___1 Articular<br>2 antes_man_clinic1___2 Dactilite<br>3 antes_man_clinic1___3 Entesite<br>4 antes_man_clinic1___4 Intestinal<br>5 antes_man_clinic1___5 Uveíte<br>0 antes_man_clinic1___0 Nenhuma<br>Custom alignment: LV<br>Field Annotation: @NOMISSING<br>@NONEOFTHEABOVE='0'
131	antes_hlab27_1<br>Show the field ONLY if:<br>[doen_reuma]='1'	HLA-B27 (qualquer data)	radio<br>1 Positivo<br>0 Negativo<br>999 Não disponível<br>Custom alignment: LV<br>Field Annotation: @NOMISSING

132	info_depois_caso1<br><br>Show the field ONLY if:<br>[caso_contr]='1' and [doen_reuma]='1'	Após os sintomas da covid-19 Avaliação no momento da inclusão	descriptive<br>Field Annotation: @NOMISSING
133	info_depois_contr1<br><br>Show the field ONLY if:<br>[caso_contr]='2' and [doen_reuma]='1'	Avaliação atual Avaliação do momento da inclusão	descriptive<br>Field Annotation: @NOMISSING
134	depois_piora_cov1<br><br>Show the field ONLY if:<br>[caso_contr]='1' and [doen_reuma]='1'	Houve piora da atividade da doença depois dos sintomas de COVID-19?	radio<br>1 Sim<br>0 Não<br>888 Desconhecido<br><br>Custom alignment: LV<br>Field Annotation: @NOMISSING
135	depois_grau_reuma1<br><br>Show the field ONLY if:<br>[doen_reuma]='1'	Qual o grau de atividade da doença no momento?<br>*Indique um valor de 0 a 10 0 = ausência de atividade e 10= atividade intensa.*	text (integer, Min: 0, Max: 10)
136	depois_asdas_vsh1<br><br>Show the field ONLY if:<br>[doen_reuma]='1'	ASDAS VSH<br>*Apenas números, separar decimal com ponto (.) ASDAS VSH: Ankylosing Spondylitis Disease Activity index calculado com VSH.*	text (number)
137	depois_asdas_pcr1<br><br>Show the field ONLY if:<br>[doen_reuma]='1'	ASDAS PCR<br>*Apenas números, separar decimal com ponto (.) ASDAS PCR: Ankylosing Spondylitis Disease Activity index calculado com PCR.*	text (number)
138	depois_info_ativ1<br><br>Show the field ONLY if:<br>[doen_reuma]='1'	Em relação às manifestações clínicas iniciais, indique o grau de atividade neste momento:	descriptive<br>Field Annotation: @NOMISSING
139	depois_intestinal1<br><br>Show the field ONLY if:<br>[doen_reuma]='1'	Intestinal	radio (Matrix)<br>0 Inalterado<br>1 Piorou<br>2 Melhorou<br><br>Field Annotation: @NOMISSING
140	depois_articular1<br><br>Show the field ONLY if:<br>[doen_reuma]='1'	Articular	radio (Matrix)<br>0 Inalterado<br>1 Piorou<br>2 Melhorou<br><br>Field Annotation: @NOMISSING
141	depois_entesite1<br><br>Show the field ONLY if:<br>[doen_reuma]='1'	Entesite	radio (Matrix)<br>0 Inalterado<br>1 Piorou<br>2 Melhorou<br><br>Field Annotation: @NOMISSING
142	depois_dactilite1<br><br>Show the field ONLY if:<br>[doen_reuma]='1'	Dactilite	radio (Matrix)<br>0 Inalterado<br>1 Piorou<br>2 Melhorou<br><br>Field Annotation: @NOMISSING
143	depois_uveit1<br><br>Show the field ONLY if:<br>[doen_reuma]='1'	Uveíte	radio (Matrix)<br>0 Inalterado<br>1 Piorou<br>2 Melhorou<br><br>Field Annotation: @NOMISSING

144	depois_piora_drim1<br>Show the field ONLY if: [caso_contr]='1' and [doen_reuma]='1'	Houve aparecimento ou piora de outra manifestação de DRIM?	radio<br>1 Sim<br>0 Não<br>Custom alignment: LV<br>Field Annotation: @NOMISSING
145	depois_piora_drim_s1<br>Show the field ONLY if: [depois_piora_drim1]='1'	Qual ?<br>*Aparecimento de outras manifestações imunomediadas não listadas acima ou não diretamente relacionada com a doença de base.*	text<br>Field Annotation: @NOMISSING
146	info_antes_caso2<br>Show the field ONLY if: [caso_contr]='1' and [doen_reuma]='2'	ARTRITE IDIOPÁTICA JUVENIL (INÍCIO ANTES DO 16 ANOS) NA IDADE ADULTA - Antes dos sintomas da covid-19 Avaliação mais recente e disponível em prontuário, de no máximo 6 meses atrás	descriptive<br>Field Annotation: @NOMISSING
147	info_antes_contr2<br>Show the field ONLY if: [caso_contr]='2' and [doen_reuma]='2'	ARTRITE IDIOPÁTICA JUVENIL (INÍCIO ANTES DO 16 ANOS) NA IDADE ADULTA - Avaliação prévia Avaliação mais recente e disponível em prontuário, de no máximo 6 meses atrás	descriptive<br>Field Annotation: @NOMISSING
148	antes_grau_reuma2<br>Show the field ONLY if: [doen_reuma]='2'	Qual o grau de atividade da doença?<br>*Indique um valor de 0 a 10 0 = ausência de atividade e 10= atividade intensa.*	text (integer, Min: 0, Max: 10)<br>Field Annotation: @NOMISSING
149	antes_man_clinic2<br>Show the field ONLY if: [doen_reuma]='2'	Assinale as manifestações clínicas em atividade	checkbox<br>1 antes_man_clinic2___1 Articular<br>2 antes_man_clinic2___2 Dactilite<br>3 antes_man_clinic2___3 Entesite<br>4 antes_man_clinic2___4 Intestinal<br>5 antes_man_clinic2___5 Uveíte<br>0 antes_man_clinic2___0 Nenhuma<br>Custom alignment: LV<br>Field Annotation: @NOMISSING @NONEOFTHEABOVE='0'
150	antes_art_dolo2<br>Show the field ONLY if: [doen_reuma]='2'	Número articulações dolorosas<br>*Apenas números. Valor entre 0 e 28.*	text (integer, Min: 0, Max: 28)
151	antes_art_ede2<br>Show the field ONLY if: [doen_reuma]='2'	Número articulações edemaciadas<br>*Apenas números. Valor entre 0 e 28.*	text (integer, Min: 0, Max: 28)
152	antes_aval_doe_pac2<br>Show the field ONLY if: [doen_reuma]='2'	Avaliação de atividade de doença pelo paciente<br>*Apenas números.*	text (integer)
153	antes_aval_doe_aval2<br>Show the field ONLY if: [doen_reuma]='2'	Avaliação de atividade de doença pelo avaliador<br>*Apenas números.*	text (integer)
154	antes_chaq2<br>Show the field ONLY if: [doen_reuma]='2'	CHAQ<br>*Apenas números. CHAQ: Child Health Assessment Questionnaire.*	text (integer)
155	antes_info_lab2<br>Show the field ONLY if: [doen_reuma]='2'	Laboratório	descriptive<br>Field Annotation: @NOMISSING
156	antes_lab_fator2<br>Show the field ONLY if: [doen_reuma]='2'	Fator reumatoide (qualquer data)<br>*Baixo título: até três vezes o valor de referência Altos títulos: mais de três vezes o valor de referência*	radio<br>1 Positivo<br>0 Negativo<br>999 Não disponível<br>Custom alignment: LV<br>Field Annotation: @NOMISSING

157	antes_lab_fan2<br>Show the field ONLY if:<br>[doen_reuma]='2'	FAN (qualquer data)	radio<br>1 Positivo<br>0 Negativo<br>999 Não disponível<br>Custom alignment: LV<br>Field Annotation: @NOMISSING
158	antes_lab_fan_titulo2<br>Show the field ONLY if:<br>[antes_lab_fan2]='1'	Título FAN	text<br>Field Annotation: @NOMISSING
159	antes_hlab27_2<br>Show the field ONLY if:<br>[doen_reuma]='2'	HLA-B27 (qualquer data)	radio<br>1 Positivo<br>0 Negativo<br>999 Não disponível<br>Custom alignment: LV<br>Field Annotation: @NOMISSING
160	antes_erosia2<br>Show the field ONLY if:<br>[doen_reuma]='2'	Doença erosiva:<br>*Radiografias simples ou US ou RM*	radio<br>1 Sim<br>0 Não<br>Custom alignment: LV<br>Field Annotation: @NOMISSING
161	info_depois_caso2<br>Show the field ONLY if:<br>[caso_contr]='1' and [doen_reuma]='2'	Após os sintomas da covid-19 Avaliação do momento da inclusão	descriptive<br>Field Annotation: @NOMISSING
162	info_depois_contr2<br>Show the field ONLY if:<br>[caso_contr]='2' and [doen_reuma]='2'	Avaliação atual Avaliação do momento da inclusão	descriptive<br>Field Annotation: @NOMISSING
163	depois_piora_cov2<br>Show the field ONLY if:<br>[caso_contr]='1' and [doen_reuma]='2'	Houve piora da atividade da doença depois dos sintomas de COVID-19?	radio<br>1 Sim<br>0 Não<br>888 Desconhecido<br>Custom alignment: LV<br>Field Annotation: @NOMISSING
164	depois_grau_reuma2<br>Show the field ONLY if:<br>[doen_reuma]='2'	Qual o grau de atividade da doença?<br>*Indique um valor de 0 a 10 0 = ausência de atividade e 10= atividade intensa.*	text (integer, Min: 0, Max: 10)
165	depois_info_ativ2<br>Show the field ONLY if:<br>[doen_reuma]='2'	Em relação às manifestações clínicas iniciais, indique o grau de atividade neste momento:	descriptive<br>Field Annotation: @NOMISSING
166	depois_intestinal2<br>Show the field ONLY if:<br>[doen_reuma]='2'	Intestinal	radio (Matrix)<br>0 Inalterado<br>1 Piorou<br>2 Melhorou<br>Field Annotation: @NOMISSING
167	depois_articular2<br>Show the field ONLY if:<br>[doen_reuma]='2'	Articular	radio (Matrix)<br>0 Inalterado<br>1 Piorou<br>2 Melhorou<br>Field Annotation: @NOMISSING

168	depois_entesite2<br><br>Show the field ONLY if:<br>[doen_reuma]='2'	Entesite	radio (Matrix)<br>0 Inalterado<br>1 Piorou<br>2 Melhorou<br><br>Field Annotation: @NOMISSING
169	depois_dactilite2<br><br>Show the field ONLY if:<br>[doen_reuma]='2'	Dactilite	radio (Matrix)<br>0 Inalterado<br>1 Piorou<br>2 Melhorou<br><br>Field Annotation: @NOMISSING
170	depois_uveit2<br><br>Show the field ONLY if:<br>[doen_reuma]='2'	Uveíte	radio (Matrix)<br>0 Inalterado<br>1 Piorou<br>2 Melhorou<br><br>Field Annotation: @NOMISSING
171	depois_piora_drim2<br><br>Show the field ONLY if:<br>[caso_contr]='1' and [doen_reuma]='2'	Houve aparecimento ou piora de outra manifestação de DRIM?	radio<br>1 Sim<br>0 Não<br><br>Custom alignment: LV<br>Field Annotation: @NOMISSING
172	depois_piora_drim_s2<br><br>Show the field ONLY if:<br>[depois_piora_drim2]='1'	Qual?<br>*Aparecimento de outras manifestações imunomediadas não listadas acima ou não diretamente relacionada com a doença de base.*	text<br>Field Annotation: @NOMISSING
173	info_antes_caso3<br><br>Show the field ONLY if:<br>[caso_contr]='1' and [doen_reuma]='3'	ARTRITE PSORIÁSICA (APS) - Antes dos sintomas da covid-19 Avaliação mais recente e disponível em prontuário, de no máximo 6 meses atrás	descriptive<br>Field Annotation: @NOMISSING
174	info_antes_contr3<br><br>Show the field ONLY if:<br>[caso_contr]='2' and [doen_reuma]='3'	ARTRITE PSORIÁSICA (APS) - Avaliação prévia Avaliação mais recente e disponível em prontuário, de no máximo 6 meses atrás	descriptive<br>Field Annotation: @NOMISSING
175	antes_grau_reuma3<br><br>Show the field ONLY if:<br>[doen_reuma]='3'	Qual o grau de atividade da doença?<br>*Indique um valor de 0 a 10 0 = ausência de atividade e 10= atividade intensa.*	text (integer, Min: 0, Max: 10)<br>Field Annotation: @NOMISSING
176	antes_ativos3<br><br>Show the field ONLY if:<br>[doen_reuma]='3'	Assinale as manifestações clínicas que estavam em atividade	checkbox<br>1 antes_ativos3___1 Artrite ativa<br>2 antes_ativos3___2 Dactilite<br>3 antes_ativos3___3 Doença Axial<br>4 antes_ativos3___4 Entesite<br>5 antes_ativos3___5 Pele ou unha<br>0 antes_ativos3___0 Nenhuma<br><br>Custom alignment: LV<br>Field Annotation: @NOMISSING @NONEOFTHEABOVE ='0'
177	antes_parametros3<br><br>Show the field ONLY if:<br>[doen_reuma]='3'	Parâmetros de atividade estão disponíveis?	radio<br>1 Sim<br>0 Não<br><br>Custom alignment: LV<br>Field Annotation: @NOMISSING
178	antes_dapsa3<br><br>Show the field ONLY if:<br>[antes_parametros3]='1'	DAPSA<br>*Apenas números. DAPSA: Disease Activity in PSoriatic Arthritis.*	text

179	antes_pasi3<br>Show the field ONLY if:<br>[antes_parametros3]='1'	PASI<br>*Apenas números. PASI: Psoriaisis Area Severity Index.*	text
180	antes_mda3<br>Show the field ONLY if:<br>[doen_reuma]='3'	Estava MDA (Mínima Atividade de Doença)<br>*5 dos 7 itens abaixo: -Articulações dolorosas ≤ 1 -Articulações edemaciadas ≤ 1 -Pontos de entesite ≤ 1 -PASI ≤1ou superfície de área corpórea ≤ 3 -Escala visual analógica de dor do paciente ≤ 15 - avaliação global da doença pelo paciente ≤ 20 - HAQ ≤ 0,5*	radio<br>1 Sim<br>0 Não<br>Custom alignment: LV<br>Field Annotation: @NOMISSING
181	antes_hlab27_3<br>Show the field ONLY if:<br>[doen_reuma]='3'	HLA-B27 (qualquer data)	radio<br>1 Positivo<br>0 Negativo<br>999 Não disponível<br>Custom alignment: LV<br>Field Annotation: @NOMISSING
182	info_depois_caso3<br>Show the field ONLY if:<br>[caso_contr]='1' and [doen_reuma]='3'	Após os sintomas da covid-19 Avaliação do momento da inclusão	descriptive<br>Field Annotation: @NOMISSING
183	info_depois_contr3<br>Show the field ONLY if:<br>[caso_contr]='2' and [doen_reuma]='3'	Avaliação atual Avaliação do momento da inclusão	descriptive<br>Field Annotation: @NOMISSING
184	depois_piora_cov3<br>Show the field ONLY if:<br>[caso_contr]='1' and [doen_reuma]='3'	Houve piora da atividade da doença depois dos sintomas de COVID-19?	radio<br>1 Sim<br>0 Não<br>888 Desconhecido<br>Custom alignment: LV<br>Field Annotation: @NOMISSING
185	depois_grau_reuma3<br>Show the field ONLY if:<br>[doen_reuma]='3'	Qual o grau de atividade da doença?<br>*Indique um valor de 0 a 10 0 = ausência de atividade e 10= atividade intensa.*	text (integer, Min: 0, Max: 10)
186	depois_ativos3<br>Show the field ONLY if:<br>[doen_reuma]='3'	Assinale as manifestações clínicas que estavam em atividade	checkbox<br>1 depois_ativos3___1 Artrite ativa<br>2 depois_ativos3___2 Dactilite<br>3 depois_ativos3___3 Doença Axial<br>4 depois_ativos3___4 Entesite<br>5 depois_ativos3___5 Pele ou unha<br>0 depois_ativos3___0 Nenhuma<br>Custom alignment: LV<br>Field Annotation: @NOMISSING @NONEOFTHEABOVE ='0'
187	depois_parametros3<br>Show the field ONLY if:<br>[doen_reuma]='3'	Parâmetros de atividade estão disponíveis?	radio<br>1 Sim<br>0 Não<br>Custom alignment: LV<br>Field Annotation: @NOMISSING
188	depois_dapsa3<br>Show the field ONLY if:<br>[depois_parametros3]='1'	DAPSA<br>*Apenas números. DAPSA: Disease Activity in PSoriatic Arthritis.*	text (integer)
189	depois_pasi3<br>Show the field ONLY if:<br>[depois_parametros3]='1'	PASI<br>*Apenas números. PASI: Psoriaisis Area Severity Index.*	text (integer)

190	depois_mda3<br><br>Show the field ONLY if:<br>[doen_reuma]='3'	Estava MDA (Mínima Atividade de Doença)<br>*5 dos 7 itens abaixo: -Articulações dolorosas ≤ 1 -Articulações edemaciadas ≤ 1 - Pontos de entesite ≤ 1 -PASI ≤1ou superfície de área corpórea ≤ 3 -Escala visual analógica de dor do paciente ≤ 15 - avaliação global da doença pelo paciente ≤ 20 - HAQ ≤ 0,5*	radio<br>1 Sim<br>0 Não<br><br>Custom alignment: LV<br>Field Annotation: @NOMISSING
191	depois_piora_atv3<br><br>Show the field ONLY if:<br>[doen_reuma]='3'	Houve piora da atividade da doença?	radio<br>1 Sim<br>0 Não<br>888 Desconhecido<br><br>Custom alignment: LV<br>Field Annotation: @NOMISSING
192	depois_piora_atv_s3<br><br>Show the field ONLY if:<br>[doen_reuma]='3'	Qual desfecho?	radio<br>1 Artrite ativa<br>2 Dactilite<br>3 Doença Axial<br>4 Entesite<br>5 Pele ou unha<br><br>Custom alignment: LV<br>Field Annotation: @NOMISSING
193	depois_piora_drim3<br><br>Show the field ONLY if:<br>[caso_contr]='1' and [doen_reuma]='3'	Houve aparecimento ou piora de outra manifestação de DRIM?	radio<br>1 Sim<br>0 Não<br><br>Custom alignment: LV<br>Field Annotation: @NOMISSING
194	depois_piora_drim_s3<br><br>Show the field ONLY if:<br>[depois_piora_drim3]='1'	Qual?<br>*Aparecimento de outras manifestações imunomediadas não listadas acima ou não diretamente relacionada com a doença de base.*	text
195	info_inicio_caso4<br><br>Show the field ONLY if:<br>([caso_contr]='1' or [caso_contr]='2') and [doen_reuma]='4'	ARTRITE REUMATOIDE	descriptive<br>Field Annotation: @NOMISSING
196	info_carac_caso4<br><br>Show the field ONLY if:<br>[caso_contr]='1' and ([doen_reuma]='4' or [predomina14]='4')	Características da doença	descriptive<br>Field Annotation: @NOMISSING
197	info_carac_contr4<br><br>Show the field ONLY if:<br>[caso_contr]='2' and ([doen_reuma]='4' or [predomina14]='4')	Características da doença	descriptive<br>Field Annotation: @NOMISSING
198	carac_fator_reum4<br><br>Show the field ONLY if:<br>[doen_reuma]='4' or [predomina14]='4'	Fator reumatoide (qualquer data)<br>*Baixo título: até três vezes o valor de referência Altos títulos: mais de três vezes o valor de referência*	radio<br>0 Negativo<br>1 Positivo, baixo títulos<br>2 Positivo, altos títulos<br>999 Não disponível<br><br>Custom alignment: LV<br>Field Annotation: @NOMISSING
199	carac_fator_reum_t4<br><br>Show the field ONLY if:<br>[carac_fator_reum4]='1'	Título	text (integer)

200	carac_anti_ccp4<br>Show the field ONLY if:<br>[doen_reuma]='4' or [predomina14]='4'	Anti CCP (qualquer data)<br>*Baixo título: até três vezes o valor de referência Altos títulos: mais de três vezes o valor de referência*	radio<br>0 Negativo<br>1 Positivo, baixo títulos<br>2 Positivo, altos títulos<br>999 Não disponível<br>Custom alignment: LV<br>Field Annotation: @NOMISSING
201	carac_anti_ccp_t4<br>Show the field ONLY if:<br>[carac_anti_ccp4]='1'	Título	text (integer)
202	carac_erosiva4<br>Show the field ONLY if:<br>[doen_reuma]='4' or [predomina14]='4'	Doença erosiva (qualquer data)<br>*Radiografias simples ou US ou RM*	radio<br>1 Sim<br>0 Não<br>999 Não disponível<br>Custom alignment: LV<br>Field Annotation: @NOMISSING
203	carac_extraart4<br>Show the field ONLY if:<br>[doen_reuma]='4' or [predomina14]='4'	Manifestação extra-articular	radio<br>1 Sim<br>0 Não<br>999 Não disponível<br>Custom alignment: LV<br>Field Annotation: @NOMISSING
204	carac_sjogren4<br>Show the field ONLY if:<br>[doen_reuma]='4' or [predomina14]='4'	Síndrome Sjögren secundária	radio<br>1 Sim<br>0 Não<br>999 Não disponível<br>Custom alignment: LV<br>Field Annotation: @NOMISSING
205	carac_pulmonar4<br>Show the field ONLY if:<br>[doen_reuma]='4' or [predomina14]='4'	Doença pulmonar intersticial	radio<br>1 Sim<br>0 Não<br>999 Não disponível<br>Custom alignment: LV<br>Field Annotation: @NOMISSING
206	info_antes_caso4<br>Show the field ONLY if:<br>[caso_contr]='1' and ([doen_reuma]='4' or [predomina14]='4')	Antes dos sintomas da covid-19 Avaliação mais recente e disponível em prontuário, de no máximo 6 meses atrás	descriptive<br>Field Annotation: @NOMISSING
207	info_antes_contr4<br>Show the field ONLY if:<br>[caso_contr]='2' and ([doen_reuma]='4' or [predomina14]='4')	Avaliação prévia Avaliação mais recente e disponível em prontuário, de no máximo 6 meses atrás	descriptive<br>Field Annotation: @NOMISSING
208	antes_grau_reuma4<br>Show the field ONLY if:<br>[doen_reuma]='4' or [predomina14]='4'	Qual o grau de atividade da doença?<br>*Indique um valor de 0 a 10 0 = ausência de atividade e 10= atividade intensa.*	text (integer, Min: 0, Max: 10)
209	antes_cdai_4<br>Show the field ONLY if:<br>[doen_reuma]='4' or [predomina14]='4'	Qual o CDAI mais recente disponível em prontuário<br>*Apenas números. CDAI: Clinical Disease Activity Index Valor entre 0 e 76.*	text (integer, Min: 0, Max: 76)
210	antes_art_dolo4<br>Show the field ONLY if:<br>[doen_reuma]='4' or [predomina14]='4'	Número articulações dolorosas<br>*Apenas números. Valor entre 0 e 28.*	text (integer, Min: 0, Max: 28)

211	antes_art_ede4<br>Show the field ONLY if:<br>[doen_reuma]='4' or [predomina14]='4'	Número articulações edemaciadas<br>*Apenas números. Valor entre 0 e 28.*	text (integer, Min: 0, Max: 28)
212	antes_aval_doe_pac4<br>Show the field ONLY if:<br>[doen_reuma]='4' or [predomina14]='4'	Avaliação de atividade de doença pelo paciente<br>*Apenas números.*	text (integer)
213	antes_aval_doe_aval4<br>Show the field ONLY if:<br>[doen_reuma]='4' or [predomina14]='4'	Avaliação de atividade de doença pelo avaliador<br>*Apenas números.*	text (integer)
214	info_depois_caso4<br>Show the field ONLY if:<br>[caso_contr]='1' and ([doen_reuma]='4' or [predomina14]='4')	Após os sintomas da covid-19 Avaliação do momento da inclusão	descriptive<br>Field Annotation: @NOMISSING
215	info_depois_contr4<br>Show the field ONLY if:<br>[caso_contr]='2' and ([doen_reuma]='4' or [predomina14]='4')	Avaliação atual Avaliação do momento da inclusão	descriptive<br>Field Annotation: @NOMISSING
216	depois_piora_cov4<br>Show the field ONLY if:<br>[caso_contr]='1' and ([doen_reuma]='4' or [predomina14]='4')	Houve piora da atividade da doença depois dos sintomas de COVID-19?	radio<br>1 Sim<br>0 Não<br>888 Desconhecido<br>Custom alignment: LV<br>Field Annotation: @NOMISSING
217	depois_grau_reuma4<br>Show the field ONLY if:<br>[doen_reuma]='4' or [predomina14]='4'	Qual o grau de atividade da doença?<br>*Indique um valor de 0 a 10 0 = ausência de atividade e 10= atividade intensa.*	text (integer, Min: 0, Max: 10)
218	depois_cdai_4<br>Show the field ONLY if:<br>[doen_reuma]='4' or [predomina14]='4'	Qual o CDAI atual?<br>*Apenas números. CDAI: Clinical Disease Activity Index Valor entre 0 e 76.*	text (integer, Min: 0, Max: 76)
219	depois_art_dolo4<br>Show the field ONLY if:<br>[doen_reuma]='4' or [predomina14]='4'	Número articulações dolorosas<br>*Apenas números. Valor entre 0 e 28.*	text (integer, Min: 0, Max: 28)
220	depois_art_ede4<br>Show the field ONLY if:<br>[doen_reuma]='4' or [predomina14]='4'	Número articulações edemaciadas<br>*Apenas números. Valor entre 0 e 28.*	text (integer, Min: 0, Max: 28)
221	depois_aval_doe_pac4<br>Show the field ONLY if:<br>[doen_reuma]='4' or [predomina14]='4'	Avaliação de atividade de doença pelo paciente<br>*Apenas números.*	text (integer)
222	depois_aval_doe_aval4<br>Show the field ONLY if:<br>[doen_reuma]='4' or [predomina14]='4'	Avaliação de atividade de doença pelo avaliador<br>*Apenas números.*	text (integer)
223	depois_piora_drim4<br>Show the field ONLY if:<br>[caso_contr]='1' and ([doen_reuma]='4' or [predomina14]='4')	Houve aparecimento ou piora de outra manifestação de DRIM?	radio<br>1 Sim<br>0 Não<br>Custom alignment: LV<br>Field Annotation: @NOMISSING
224	depois_piora_drim_s4<br>Show the field ONLY if:<br>[depois_piora_drim4]='1'	Qual?<br>*Aparecimento de outras manifestações imunomediadas não listadas acima ou não diretamente relacionada com a doença de base.*	text<br>Field Annotation: @NOMISSING

225	info_antes_caso5<br><br>Show the field ONLY if:<br>[caso_contr]='1' and [doen_reuma]='5'	DOENÇA DE BEHÇET - Antes dos sintomas da covid-19 Avaliação mais recente e disponível em prontuário, de no máximo 6 meses atrás	descriptive<br>Field Annotation: @NOMISSING
226	info_antes_contr5<br><br>Show the field ONLY if:<br>[caso_contr]='2' and [doen_reuma]='5'	DOENÇA DE BEHÇET- Avaliação prévia Avaliação mais recente e disponível em prontuário, de no máximo 6 meses atrás	descriptive<br>Field Annotation: @NOMISSING
227	antes_grau_reuma5<br><br>Show the field ONLY if:<br>[antes_ativos5(1)]='1'	Qual o grau de atividade da doença?<br>*Indique um valor de 0 a 10 0 = ausência de atividade e 10= atividade intensa.*	text (integer, Min: 0, Max: 10)
228	antes_ativos5<br><br>Show the field ONLY if:<br>[doen_reuma]='5'	Assinale as manifestações clínicas que estavam em atividade	checkbox<br>1 antes_ativos5___1 Fenômenos tromboembólicos<br>2 antes_ativos5___2 Lesões de pele (eritema nodoso/foliculite)<br>3 antes_ativos5___3 Manifestações neurológicas<br>4 antes_ativos5___4 Ocular<br>5 antes_ativos5___5 Sistema nervoso central<br>6 antes_ativos5___6 Sistema nervoso periférico<br>7 antes_ativos5___7 Úlceras genitais<br>8 antes_ativos5___8 Úlceras orais<br>0 antes_ativos5___0 Nenhuma<br><br>Custom alignment: LV<br>Field Annotation: @NOMISSING @NONEOFTHEABOVE ='0'
229	antes_sitio5<br><br>Show the field ONLY if:<br>[antes_ativos5(1)]='1'	Qual sítio?	radio<br>1 AVC<br>2 Oclusão Arterial Aguda<br>3 Retina<br>4 Tromboembolismo Pulmonar<br>5 Tromboflebite superficial<br>6 Trombose Venosa Profunda<br>777 Outros<br><br>Custom alignment: LV<br>Field Annotation: @NOMISSING
230	antes_sitio_out5<br><br>Show the field ONLY if:<br>[antes_sitio5]='777'	Qual outro sítio?	text
231	antes_brbdcaf5<br><br>Show the field ONLY if:<br>[doen_reuma]='5'	BR-BDCAFs ou BRBDCAF disponível?<br>*BR-BDCAFs: Brazilian Behçet's Disease Current Activity Form simplificado BRBDCAF: Brazilian Behçet's Disease Current Activity Form*	radio<br>1 Sim<br>0 Não<br><br>Custom alignment: LV<br>Field Annotation: @NOMISSING
232	info_depois_caso5<br><br>Show the field ONLY if:<br>[caso_contr]='1' and [doen_reuma]='5'	Após os sintomas da covid-19 Avaliação do momento da inclusão	descriptive<br>Field Annotation: @NOMISSING
233	info_depois_contr5<br><br>Show the field ONLY if:<br>[caso_contr]='2' and [doen_reuma]='5'	Avaliação atual Avaliação do momento da inclusão	descriptive<br>Field Annotation: @NOMISSING

234	depois_piora_cov5<br><br>Show the field ONLY if:<br>[caso_contr]='1' and [doen_reuma]='5'	Houve piora da atividade da doença depois dos sintomas de COVID-19?	radio<br>1 Sim<br>0 Não<br>888 Desconhecido<br><br>Custom alignment: LV<br>Field Annotation: @NOMISSING
235	depois_grau_reuma5<br><br>Show the field ONLY if:<br>[depois_piora_cov5]='5'	Qual o grau de atividade da doença?<br>*Indique um valor de 0 a 10 0 = ausência de atividade e 10= atividade intensa.*	text (integer, Min: 0, Max: 10)
236	depois_info_ativ5<br><br>Show the field ONLY if:<br>[doen_reuma]='5'	Em relação às manifestações clínicas iniciais, indique o grau de atividade neste momento:	descriptive<br>Field Annotation: @NOMISSING
237	depois_lesoes_pele5<br><br>Show the field ONLY if:<br>[doen_reuma]='5'	Lesões de pele (eritema nodoso/foliculite	radio (Matrix)<br>0 Inalterado<br>2 Piorou<br>1 Melhorou<br><br>Field Annotation: @NOMISSING
238	depois_manifest5<br><br>Show the field ONLY if:<br>[doen_reuma]='5'	Manifestações neurológicas	radio (Matrix)<br>0 Inalterado<br>2 Piorou<br>1 Melhorou<br><br>Field Annotation: @NOMISSING
239	depois_ocular5<br><br>Show the field ONLY if:<br>[doen_reuma]='5'	Ocular	radio (Matrix)<br>0 Inalterado<br>2 Piorou<br>1 Melhorou<br><br>Field Annotation: @NOMISSING
240	depois_nerv_central5<br><br>Show the field ONLY if:<br>[doen_reuma]='5'	Sistema nervoso central	radio (Matrix)<br>0 Inalterado<br>2 Piorou<br>1 Melhorou<br><br>Field Annotation: @NOMISSING
241	depois_nerv_perif5<br><br>Show the field ONLY if:<br>[doen_reuma]='5'	Sistema nervoso periférico	radio (Matrix)<br>0 Inalterado<br>2 Piorou<br>1 Melhorou<br><br>Field Annotation: @NOMISSING
242	depois_ulc_genit5<br><br>Show the field ONLY if:<br>[doen_reuma]='5'	Úlceras genitais	radio (Matrix)<br>0 Inalterado<br>2 Piorou<br>1 Melhorou<br><br>Field Annotation: @NOMISSING
243	depois_ulc_oral5<br><br>Show the field ONLY if:<br>[doen_reuma]='5'	Úlceras orais	radio (Matrix)<br>0 Inalterado<br>2 Piorou<br>1 Melhorou<br><br>Field Annotation: @NOMISSING

244	depois_novos_fenom5<br>Show the field ONLY if:<br>[doen_reuma]='5'	Novos fenômenos tromboembólicos	radio<br>1 Sim<br>0 Não<br>Custom alignment: LV<br>Field Annotation: @NOMISSING
245	depois_sitio5<br>Show the field ONLY if:<br>[depois_novos_fenom5]='1'	Qual sítio do novo fenômeno tromboembólicos?	radio<br>1 AVC<br>2 Oclusão Arterial Aguda<br>3 Retina<br>4 Tromboembolismo Pulmonar<br>5 Tromboflebite superficial<br>6 Trombose Venosa Profunda<br>777 Outros<br>Custom alignment: LV<br>Field Annotation: @NOMISSING
246	depois_sitio_out5<br>Show the field ONLY if:<br>[depois_sitio5]='777'	Qual OUTRO sítio do novo fenômeno tromboembólicos?	text<br>Field Annotation: @NOMISSING
247	depois_piora_drim5<br>Show the field ONLY if:<br>[caso_contr]='1' and [doen_reuma]='5'	Houve aparecimento ou piora de outra manifestação de DRIM?	radio<br>1 Sim<br>0 Não<br>Custom alignment: LV<br>Field Annotation: @NOMISSING
248	depois_piora_drim_s5<br>Show the field ONLY if:<br>[depois_piora_drim5]='1'	Qual?<br>*Aparecimento de outras manifestações imunomediadas não listadas acima ou não diretamente relacionada com a doença de base.*	text<br>Field Annotation: @NOMISSING
249	info_antes_caso6<br>Show the field ONLY if:<br>[caso_contr]='1' and [doen_reuma]='6'	DOENÇA DE STILL DO ADULTO - Antes dos sintomas da covid-19 Avaliação mais recente e disponível em prontuário, de no máximo 6 meses atrás	descriptive<br>Field Annotation: @NOMISSING
250	info_antes_contr6<br>Show the field ONLY if:<br>[caso_contr]='2' and [doen_reuma]='6'	DOENÇA DE STILL DO ADULTO - Avaliação prévia Avaliação mais recente e disponível em prontuário, de no máximo 6 meses atrás	descriptive<br>Field Annotation: @NOMISSING
251	antes_grau_reuma6<br>Show the field ONLY if:<br>[doen_reuma]='6'	Qual o grau de atividade da doença?<br>*Indique um valor de 0 a 10 0 = ausência de atividade e 10= atividade intensa.*	text (integer, Min: 0, Max: 10)
252	antes_art_dolo6<br>Show the field ONLY if:<br>[doen_reuma]='6'	Número articulações dolorosas<br>*Apenas números. Valor entre 0 e 28.*	text (integer, Min: 0, Max: 28)
253	antes_art_ede6<br>Show the field ONLY if:<br>[doen_reuma]='6'	Número articulações edemaciadas<br>*Apenas números. Valor entre 0 e 28.*	text (integer, Min: 0, Max: 28)
254	antes_aval_doe_pac6<br>Show the field ONLY if:<br>[doen_reuma]='6'	Avaliação de atividade de doença pelo paciente<br>*Apenas números.*	text (integer)
255	antes_aval_doe_aval6<br>Show the field ONLY if:<br>[doen_reuma]='6'	Avaliação de atividade de doença pelo avaliador<br>*Apenas números.*	text (integer)

256	antes_ativos6<br><br>Show the field ONLY if:<br>[doen_reuma]='6'	Assinale as manifestações clínicas que estavam em atividade	checkbox<br>1 antes_ativos6___1 Adenomegalia<br>2 antes_ativos6___2 Esplenomegalia<br>3 antes_ativos6___3 Picos de febre<br>4 antes_ativos6___4 Rash<br>5 antes_ativos6___5 Serosites<br>0 antes_ativos6___0 Nenhuma<br><br>Custom alignment: LV<br>Field Annotation: @NOMISSING @NONEOFTHEABOVE ='0'
257	antes_lab6<br><br>Show the field ONLY if:<br>[doen_reuma]='6'	Assinale as alterações laboratoriais presentes<br>*Picos de febre considerar acima de 39C pelo menos 2 ao dia; PAI provas de atividade inflamatórias, VHS ou PCR*	checkbox<br>1 antes_lab6___1 Anemia<br>2 antes_lab6___2 Ferritina aumentada<br>3 antes_lab6___3 Leucocitose<br>4 antes_lab6___4 PAI elevadas<br>5 antes_lab6___5 Plaquetose<br>0 antes_lab6___0 Nenhuma<br><br>Custom alignment: LV<br>Field Annotation: @NOMISSING @NONEOFTHEABOVE ='0'
258	antes_lab_valor6<br><br>Show the field ONLY if:<br>[antes_lab6(2)]='1'	Valor Ferritina	text
259	info_depois_caso6<br><br>Show the field ONLY if:<br>[caso_contr]='1' and [doen_reuma]='6'	Após os sintomas da covid-19 Avaliação do momento da inclusão	descriptive<br>Field Annotation: @NOMISSING
260	info_depois_contr6<br><br>Show the field ONLY if:<br>[caso_contr]='2' and [doen_reuma]='6'	Avaliação atual Avaliação do momento da inclusão	descriptive<br>Field Annotation: @NOMISSING
261	depois_piora_cov6<br><br>Show the field ONLY if:<br>[caso_contr]='1' and [doen_reuma]='6'	Houve piora da atividade da doença depois dos sintomas de COVID-19?	radio<br>1 Sim<br>0 Não<br>888 Desconhecido<br><br>Custom alignment: LV<br>Field Annotation: @NOMISSING
262	depois_grau_reuma6<br><br>Show the field ONLY if:<br>[doen_reuma]='6'	Qual o grau de atividade da doença?<br>*Indique um valor de 0 a 10 0 = ausência de atividade e 10= atividade intensa.*	text (integer, Min: 0, Max: 10)
263	depois_adenome6<br><br>Show the field ONLY if:<br>[doen_reuma]='6'	Adenomegalia	radio (Matrix)<br>0 Inalterado<br>2 Piorou<br>1 Melhorou<br><br>Field Annotation: @NOMISSING
264	depois_articular6<br><br>Show the field ONLY if:<br>[doen_reuma]='6'	Articular	radio (Matrix)<br>0 Inalterado<br>2 Piorou<br>1 Melhorou<br><br>Field Annotation: @NOMISSING

265	depois_espeno6<br>Show the field ONLY if:<br>[doen_reuma]='6'	Espenomegalia	radio (Matrix)<br>0 Inalterado<br>2 Piorou<br>1 Melhorou<br>Field Annotation: @NOMISSING
266	depois_febre6<br>Show the field ONLY if:<br>[doen_reuma]='6'	Febre	radio (Matrix)<br>0 Inalterado<br>2 Piorou<br>1 Melhorou<br>Field Annotation: @NOMISSING
267	depois_laborat6<br>Show the field ONLY if:<br>[doen_reuma]='6'	Laboratorial	radio (Matrix)<br>0 Inalterado<br>2 Piorou<br>1 Melhorou<br>Field Annotation: @NOMISSING
268	depois_rash6<br>Show the field ONLY if:<br>[doen_reuma]='6'	Rash	radio (Matrix)<br>0 Inalterado<br>2 Piorou<br>1 Melhorou<br>Field Annotation: @NOMISSING
269	depois_serosit6<br>Show the field ONLY if:<br>[doen_reuma]='6'	Serosites	radio (Matrix)<br>0 Inalterado<br>2 Piorou<br>1 Melhorou<br>Field Annotation: @NOMISSING
270	depois_piora_drim6<br>Show the field ONLY if:<br>[caso_contr]='1' and [doen_reuma]='6'	Houve aparecimento ou piora de outra manifestação de DRIM?	radio<br>1 Sim<br>0 Não<br>Custom alignment: LV<br>Field Annotation: @NOMISSING
271	depois_piora_drim_s6<br>Show the field ONLY if:<br>[depois_piora_drim6]='1'	Qual?<br>*Aparecimento de outras manifestações imunomediadas não listadas acima ou não diretamente relacionada com a doença de base.*	text<br>Field Annotation: @NOMISSING
272	info_antes_caso7<br>Show the field ONLY if:<br>[caso_contr]='1' and [doen_reuma]='7'	DOENÇA MISTA DO TECIDO CONJUNTIVO - Antes dos sintomas da covid-19 Avaliação mais recente e disponível em prontuário, de no máximo 6 meses atrás	descriptive<br>Field Annotation: @NOMISSING
273	info_antes_contr7<br>Show the field ONLY if:<br>[caso_contr]='2' and [doen_reuma]='7'	DOENÇA MISTA DO TECIDO CONJUNTIVO - Avaliação prévia Avaliação mais recente e disponível em prontuário, de no máximo 6 meses atrás	descriptive<br>Field Annotation: @NOMISSING
274	antes_grau_reuma7<br>Show the field ONLY if:<br>[doen_reuma]='7'	Qual o grau de atividade da doença?<br>*Indique um valor de 0 a 10 0 = ausência de atividade e 10= atividade intensa.*	text (integer, Min: 0, Max: 10)

275	antes_atividade7<br><br>Show the field ONLY if:<br>[doen_reuma]='7'	Indique as manifestações clínicas em atividade na ultima avaliação:	checkbox<br>1 | antes_atividade7___1 | Artrite<br>2 | antes_atividade7___2 | Dismotilidade esofágica<br>3 | antes_atividade7___3 | Doença intersticial pulmonar<br>4 | antes_atividade7___4 | Espessamento de pele<br>5 | antes_atividade7___5 | Fenômeno de Raynaud<br>6 | antes_atividade7___6 | Glomerulonefrite<br>7 | antes_atividade7___7 | Hipertensão arterial pulmonar<br>8 | antes_atividade7___8 | Lesões de pele (rash malar ou outras lesões inflamatórias)<br>9 | antes_atividade7___9 | Neuropatia periférica<br>10 | antes_atividade7___10 | Vasculite cutânea<br>0 | antes_atividade7___0 | Nenhuma<br><br>Custom alignment: LV<br>Field Annotation: @NOMISSING @NONEOFTHEABOVE='0'
276	antes_lab_fan7<br><br>Show the field ONLY if:<br>[doen_reuma]='7'	FAN (qualquer data)	radio<br>1 | Positivo<br>0 | Negativo<br>999 | Não disponível<br><br>Custom alignment: LV<br>Field Annotation: @NOMISSING
277	antes_lab_fan_t<br><br>Show the field ONLY if:<br>[antes_lab_fan7]='1'	Título FAN	text
278	antes_lab_padrao7<br><br>Show the field ONLY if:<br>[antes_lab_fan7]='1'	Padrão	text
279	antes_anti_rnp7<br><br>Show the field ONLY if:<br>[doen_reuma]='7'	Anti-RNP	radio<br>0 | Negativo<br>1 | Positivo<br><br>Custom alignment: LV<br>Field Annotation: @NOMISSING
280	antes_anti_rnp_titulo7<br><br>Show the field ONLY if:<br>[antes_anti_rnp7]='1'	Título	text
281	antes_fator_reum4<br><br>Show the field ONLY if:<br>[doen_reuma]='7'	Fator reumatoide (qualquer data)<br>*Baixo título: até três vezes o valor de referência Altos títulos: mais de três vezes o valor de referência*	radio<br>0 | Negativo<br>1 | Positivo, baixo títulos<br>2 | Positivo, altos títulos<br>999 | Não disponível<br><br>Custom alignment: LV<br>Field Annotation: @NOMISSING
282	antes_fator_reum_t7<br><br>Show the field ONLY if:<br>[carac_fator_reum4]='1'	Título	text (integer)

283	antes_anti_ccp7<br><br>Show the field ONLY if:<br>[doen_reuma]='7'	Anti CCP (qualquer data)<br>*Baixo título: até três vezes o valor de referência Altos títulos: mais de três vezes o valor de referência*	radio<br>0 Negativo<br>1 Positivo, baixo títulos<br>2 Positivo, altos títulos<br>999 Não disponível<br><br>Custom alignment: LV<br>Field Annotation: @NOMISSING
284	antes_anti_ccp_t7<br><br>Show the field ONLY if:<br>[antes_anti_ccp7]='1'	Título	text (integer)
285	info_depois_caso7<br><br>Show the field ONLY if:<br>[caso_contr]='1' and [doen_reuma]='7'	Após os sintomas da covid-19 Avaliação do momento da inclusão	descriptive<br>Field Annotation: @NOMISSING
286	info_depois_contr7<br><br>Show the field ONLY if:<br>[caso_contr]='2' and [doen_reuma]='7'	Avaliação atual Avaliação do momento da inclusão	descriptive<br>Field Annotation: @NOMISSING
287	depois_piora_cov7<br><br>Show the field ONLY if:<br>[caso_contr]='1' and [doen_reuma]='7'	Houve piora da atividade da doença depois dos sintomas de COVID-19?	radio<br>1 Sim<br>0 Não<br>888 Desconhecido<br><br>Custom alignment: LV<br>Field Annotation: @NOMISSING
288	depois_grau_reuma7<br><br>Show the field ONLY if:<br>[doen_reuma]='7'	Qual o grau de atividade da doença?<br>*Indique um valor de 0 a 10 0 = ausência de atividade e 10= atividade intensa.*	text (integer, Min: 0, Max: 10)
289	depois_atividade7<br><br>Show the field ONLY if:<br>[doen_reuma]='7'	Em relação as manifestações clínicas, assinale o estado atual:	checkbox<br>1 depois_atividade7___1 Artrite<br>2 depois_atividade7___2 Dismotilidade esofágica<br>3 depois_atividade7___3 Doença intersticial pulmonar<br>4 depois_atividade7___4 Espessamento de pele<br>5 depois_atividade7___5 Fenômeno de Raynaud<br>6 depois_atividade7___6 Glomerulonefrite<br>7 depois_atividade7___7 Hipertensão arterial pulmonar<br>8 depois_atividade7___8 Lesões de pele (rash malar ou outras lesões inflamatórias)<br>9 depois_atividade7___9 Neuropatia periférica<br>10 depois_atividade7___10 Vasculite cutânea<br>0 depois_atividade7___0 Nenhuma<br><br>Custom alignment: LV<br>Field Annotation: @NOMISSING @NONEOFTHEABOVE ='0'
290	depois_piora_drim7<br><br>Show the field ONLY if:<br>[caso_contr]='1' and [doen_reuma]='7'	Houve aparecimento ou piora de outra manifestação de DRIM?	radio<br>1 Sim<br>0 Não<br><br>Custom alignment: LV<br>Field Annotation: @NOMISSING
291	depois_piora_drim_s7<br><br>Show the field ONLY if:<br>[depois_piora_drim7]='1'	Qual?<br>*Aparecimento de outras manifestações imunomediadas não listadas acima ou não diretamente relacionada com a doença de base.*	text<br>Field Annotation: @NOMISSING

292	info_inicio_caso8<br>Show the field ONLY if:<br>([caso_contr]='1' or [caso_contr]='2') and [doen_reuma]='8'	ESCLEROSE SISTÊMICA	descriptive<br>Field Annotation: @NOMISSING
293	info_antes_caso8<br>Show the field ONLY if:<br>[caso_contr]='1' and ([doen_reuma]='8' or [predomina14]='8')	Avaliação mais recente e disponível em prontuário, de no máximo 6 meses atrás	descriptive<br>Field Annotation: @NOMISSING
294	info_antes_contr8<br>Show the field ONLY if:<br>[caso_contr]='2' and ([doen_reuma]='8' or [predomina14]='8')	Avaliação mais recente e disponível em prontuário, de no máximo 6 meses atrás	descriptive<br>Field Annotation: @NOMISSING
295	antes_grau_reuma8<br>Show the field ONLY if:<br>[doen_reuma]='8' or [predomina14]='8'	Qual o grau de atividade da doença?<br>*Indique um valor de 0 a 10 0 = ausência de atividade e 10= atividade intensa.*	text (integer, Min: 0, Max: 10)
296	antes_atividade8<br>Show the field ONLY if:<br>[doen_reuma]='8' or [predomina14]='8'	Assinale as manifestações clínicas em atividade:	checkbox<br>8 | antes_atividade8___8 | Articular<br>1 | antes_atividade8___1 | Doença intersticial pulmonar<br>2 | antes_atividade8___2 | Espessamento da pele<br>3 | antes_atividade8___3 | Fenômeno de Raynaud<br>4 | antes_atividade8___4 | Gastrointestinal<br>5 | antes_atividade8___5 | Hipertensão arterial pumonar<br>9 | antes_atividade8___9 | Muscular<br>6 | antes_atividade8___6 | Renal (crise renal esclerodérmica)<br>7 | antes_atividade8___7 | Úlceras digitais<br>0 | antes_atividade8___0 | Nenhuma<br>Custom alignment: LV<br>Field Annotation: @NOMISSING @NONEOFTHEABOVE='0'
297	antes_rodnan8<br>Show the field ONLY if:<br>[doen_reuma]='8' or [predomina14]='8'	Escore de Rodnan dos últimos 6 meses disponível?	radio<br>1 | Sim<br>0 | Não<br>Custom alignment: LV<br>Field Annotation: @NOMISSING
298	antes_rodnan_s8<br>Show the field ONLY if:<br>[antes_rodnan8]='1'	Valor Escore de Rodnan	text
299	antes_distancia8<br>Show the field ONLY if:<br>[doen_reuma]='8' or [predomina14]='8'	Teste de caminhada em 6 minutos mais recente disponível em prontuário (dos últimos 6 meses):istância percorrida:	text
300	antes_pulmonar8<br>Show the field ONLY if:<br>[doen_reuma]='8' or [predomina14]='8'	Anote o resultado da prova de função pulmonar mais recente disponível em prontuário (dos últimos 6 meses):	descriptive
301	antes_cvf8<br>Show the field ONLY if:<br>[doen_reuma]='8' or [predomina14]='8'	CVF<br>*Apenas números. CVF: Capacidade Vital Forçada*	text (integer)
302	antes_dlco8<br>Show the field ONLY if:<br>[doen_reuma]='8' or [predomina14]='8'	DLCO<br>*Apenas números. DLCO: Capacidade de Difusão do Monóxido de Carbono*	text (integer)

303	antes_lab_fan8<br>Show the field ONLY if:<br>[doen_reuma]='8' or [predomina14]='8'	FAN (qualquer data)	radio<br>1 Positivo<br>0 Negativo<br>999 Não disponível<br>Field Annotation: @NOMISSING
304	antes_lab_fan_t8<br>Show the field ONLY if:<br>[antes_lab_fan8]='1'	Título FAN	text
305	antes_lab_padrao8<br>Show the field ONLY if:<br>[antes_lab_fan8]='1'	Padrão	text
306	info_depois_caso8<br>Show the field ONLY if:<br>[caso_contr]='1' and ([doen_reuma]='8' or [predomina14]='8')	Após os sintomas da covid-19 Avaliação do momento da inclusão	descriptive<br>Field Annotation: @NOMISSING
307	info_depois_contr8<br>Show the field ONLY if:<br>[caso_contr]='2' and ([doen_reuma]='8' or [predomina14]='8')	Avaliação atual Avaliação do momento da inclusão	descriptive<br>Field Annotation: @NOMISSING
308	depois_piora_cov8<br>Show the field ONLY if:<br>[caso_contr]='1' and ([doen_reuma]='8' or [predomina14]='8')	Houve piora da atividade da doença depois dos sintomas de COVID-19?	radio<br>1 Sim<br>0 Não<br>888 Desconhecido<br>Custom alignment: LV<br>Field Annotation: @NOMISSING
309	depois_grau_reuma8<br>Show the field ONLY if:<br>[doen_reuma]='8' or [predomina14]='8'	Qual o grau de atividade da doença?<br>*Indique um valor de 0 a 10 0 = ausência de atividade e 10= atividade intensa.*	text (integer, Min: 0, Max: 10)
310	depois_artrite<br>Show the field ONLY if:<br>[doen_reuma]='8' or [predomina14]='8'	Artrite	radio (Matrix)<br>0 Inalterado<br>2 Piorou<br>1 Melhorou<br>Field Annotation: @NOMISSING
311	depois_intersticial8<br>Show the field ONLY if:<br>[doen_reuma]='8' or [predomina14]='8'	Doença intersticial pulmonar	radio (Matrix)<br>0 Inalterado<br>2 Piorou<br>1 Melhorou<br>Field Annotation: @NOMISSING
312	depois_espessam8<br>Show the field ONLY if:<br>[doen_reuma]='8' or [predomina14]='8'	Espessamento da pele	radio (Matrix)<br>0 Inalterado<br>2 Piorou<br>1 Melhorou<br>Field Annotation: @NOMISSING
313	depois_raynaud8<br>Show the field ONLY if:<br>[doen_reuma]='8' or [predomina14]='8'	Fenômeno de Raynaud	radio (Matrix)<br>0 Inalterado<br>2 Piorou<br>1 Melhorou<br>Field Annotation: @NOMISSING

314	depois_gastro8<br>Show the field ONLY if:<br>[doen_reuma]='8' or [predomina14]='8'	Gastrointestinal	radio (Matrix)<br>0 Inalterado<br>2 Piorou<br>1 Melhorou<br>Field Annotation: @NOMISSING
315	depois_hipert8<br>Show the field ONLY if:<br>[doen_reuma]='8' or [predomina14]='8'	Hipertensão arterial pumonar	radio (Matrix)<br>0 Inalterado<br>2 Piorou<br>1 Melhorou<br>Field Annotation: @NOMISSING
316	depois_muscular<br>Show the field ONLY if:<br>[doen_reuma]='8' or [predomina14]='8'	Muscular	radio (Matrix)<br>0 Inalterado<br>2 Piorou<br>1 Melhorou<br>Field Annotation: @NOMISSING
317	depois_renal8<br>Show the field ONLY if:<br>[doen_reuma]='8' or [predomina14]='8'	Renal (crise renal esclerodérmica)	radio (Matrix)<br>0 Inalterado<br>2 Piorou<br>1 Melhorou<br>Field Annotation: @NOMISSING
318	depois_ulcera8<br>Show the field ONLY if:<br>[doen_reuma]='8' or [predomina14]='8'	Úlceras digitais	radio (Matrix)<br>0 Inalterado<br>2 Piorou<br>1 Melhorou<br>Field Annotation: @NOMISSING
319	depois_rodnan8<br>Show the field ONLY if:<br>[doen_reuma]='8' or [predomina14]='8'	Escore de Rodnan dos últimos 6 meses disponível?	radio<br>1 Sim<br>0 Não<br>Custom alignment: LV<br>Field Annotation: @NOMISSING
320	depois_rodnan_s8<br>Show the field ONLY if:<br>[depois_rodnan8]='1'	Valor Escore de Rodnan<br>*Apenas números. CVF: Capacidade Vital Forçada*	text (integer)
321	depois_distancia8<br>Show the field ONLY if:<br>[doen_reuma]='8' or [predomina14]='8'	Teste de caminhada em 6 minutos mais recente disponível em prontuário (dos últimos 6 meses):Distância percorrida	text
322	depois_pulmonar8<br>Show the field ONLY if:<br>[doen_reuma]='8' or [predomina14]='8'	Anote o resultado da prova de função pulmonar mais recente disponível em prontuário (dos últimos 6 meses):	descriptive
323	depois_cvf8<br>Show the field ONLY if:<br>[doen_reuma]='8' or [predomina14]='8'	CVF<br>*Apenas números. CVF: Capacidade Vital Forçada*	text (integer)
324	depois_dlco8<br>Show the field ONLY if:<br>[doen_reuma]='8' or [predomina14]='8'	DLCO<br>*Apenas números. DLCO: Capacidade de Difusão do Monóxido de Carbono*	text (integer)

325	depois_tomograf8<br>Show the field ONLY if:<br>[doen_reuma]='8' or [predomina14]='8'	Tomografia computadorizada de tórax disponível?	radio<br>1 Sim<br>0 Não<br><br>Custom alignment: LV<br>Field Annotation: @NOMISSING
326	depois_tomograf_s8<br>Show the field ONLY if:<br>[depois_tomograf8]='1'	Tomografia computadorizada de tórax	radio<br>0 Inalterado<br>2 Piorou<br>1 Melhorou<br><br>Custom alignment: LV<br>Field Annotation: @NOMISSING
327	depois_piora_drim8<br>Show the field ONLY if:<br>[caso_contr]='1' and ([doen_reuma]='8' or [predomina14]='8')	Houve aparecimento ou piora de outra manifestação de DRIM?	radio<br>1 Sim<br>0 Não<br><br>Custom alignment: LV<br>Field Annotation: @NOMISSING
328	depois_piora_drim_s8<br>Show the field ONLY if:<br>[depois_piora_drim8]='1'	Qual?<br>*Aparecimento de outras manifestações imunomediadas não listadas acima ou não diretamente relacionada com a doença de base.*	text<br>Field Annotation: @NOMISSING
329	info_antes_caso9<br>Show the field ONLY if:<br>[caso_contr]='1' and [doen_reuma]='9'	ESPONDILOARTRITE AXIAL (espondilite anquilosante e não radiográfica): EpA axial - Antes dos sintomas da covid-19 Avaliação mais recente e disponível em prontuário, de no máximo 6 meses atrás	descriptive<br>Field Annotation: @NOMISSING
330	info_antes_contr9<br>Show the field ONLY if:<br>[caso_contr]='2' and [doen_reuma]='9'	ESPONDILOARTRITE AXIAL (espondilite anquilosante e não radiográfica): EpA axial - Avaliação prévia Avaliação mais recente e disponível em prontuário, de no máximo 6 meses atrás	descriptive<br>Field Annotation: @NOMISSING
331	antes_grau_reuma9<br>Show the field ONLY if:<br>[doen_reuma]='9'	Qual o grau de atividade da doença?<br>*Indique um valor de 0 a 10 0 = ausência de atividade e 10= atividade intensa.*	text (integer, Min: 0, Max: 10)
332	antes_basdai9<br>Show the field ONLY if:<br>[doen_reuma]='9'	BASDAI<br>*Apenas números. Separar decimais com ponto (.) BASDAI: Bath Ankyloging Spondylitis Disease Activity Score*	text (number)
333	antes_asdas_vhs9<br>Show the field ONLY if:<br>[doen_reuma]='9'	ASDAS-VHS<br>*Apenas números, separar decimal com ponto (.) ASDAS VHS: Ankylosing Spondylitis Disease Activity Score calculado com VSH*	text (number)
334	antes_asdas_pcr9<br>Show the field ONLY if:<br>[doen_reuma]='9'	ASDAS-PCR<br>*Apenas números, separar decimal com ponto (.) ASDAS PCR: Ankylosing Spondylitis Disease Activity Score calculado com PCR*	text (number)
335	antes_atividades9<br>Show the field ONLY if:<br>[doen_reuma]='9'	Assinale as manifestações clínicas em atividade	checkbox<br>1 antes_atividades9___1 Artrite periférica<br>2 antes_atividades9___2 Axial<br>3 antes_atividades9___3 Entesite<br>4 antes_atividades9___4 Uveíte<br>0 antes_atividades9___0 Nenhuma<br><br>Custom alignment: LV<br>Field Annotation: @NOMISSING<br>@NONEOFTHEABOVE='0'
336	antes_hlab27_9<br>Show the field ONLY if:<br>[doen_reuma]='9'	HLA-B27 (qualquer data)	radio<br>1 Positivo<br>0 Negativo<br>999 Não disponível<br><br>Custom alignment: LV

337	info_depois_caso9<br>Show the field ONLY if: [caso_contr]='1' and [doen_reuma]='9'	Após os sintomas da covid-19 Avaliação do momento da inclusão	descriptive<br>Field Annotation: @NOMISSING
338	info_depois_contr9<br>Show the field ONLY if: [caso_contr]='2' and [doen_reuma]='9'	Avaliação atual Avaliação do momento da inclusão	descriptive<br>Field Annotation: @NOMISSING
339	depois_piora_cov9<br>Show the field ONLY if: [caso_contr]='1' and [doen_reuma]='9'	Houve piora da atividade da doença depois dos sintomas de COVID-19?	radio<br>1 Sim<br>0 Não<br>888 Desconhecido<br>Custom alignment: LV<br>Field Annotation: @NOMISSING
340	depois_piora_s_cov19<br>Show the field ONLY if: [depois_piora_cov9]='1'	Qual ?	radio<br>1 Articulares axiais<br>2 Extra-articulares<br>3 Periféricas<br>Custom alignment: LV<br>Field Annotation: @NOMISSING
341	depois_grau_reuma9<br>Show the field ONLY if: [doen_reuma]='9'	Qual o grau de atividade da doença?<br>*Indique um valor de 0 a 10 0 = ausência de atividade e 10= atividade intensa.*	text (integer, Min: 0, Max: 10)
342	depois_basdai9<br>Show the field ONLY if: [doen_reuma]='9'	BASDAI<br>*Apenas números. Separar decimais com ponto (.) BASDAI: Bath Ankyloging Spondylitis Disease Activity Score*	text (number)
343	depois_asdas_vhs9<br>Show the field ONLY if: [doen_reuma]='9'	ASDAS-VHS<br>*Apenas números, separar decimal com ponto (.) ASDAS VHS: Ankylosing Spondylitis Disease Activity Score calculado com VSH*	text (number)
344	depois_asdas_pcr9<br>Show the field ONLY if: [doen_reuma]='9'	ASDAS-PCR<br>*Apenas números, separar decimal com ponto (.) ASDAS PCR: Ankylosing Spondylitis Disease Activity Score calculado com PCR*	text (number)
345	depois_piora_drim9<br>Show the field ONLY if: [caso_contr]='1' and [doen_reuma]='9'	Houve aparecimento ou piora de outra manifestação de DRIM?	radio<br>1 Sim<br>0 Não<br>Custom alignment: LV<br>Field Annotation: @NOMISSING
346	depois_piora_drim_s9<br>Show the field ONLY if: [depois_piora_drim9]='1'	Qual?<br>*Aparecimento de outras manifestações imunomediadas não listadas acima ou não diretamente relacionada com a doença de base.*	text<br>Field Annotation: @NOMISSING
347	info_inicio_caso10<br>Show the field ONLY if: ([caso_contr]='1' or [caso_contr]='2') and [doen_reuma]='10'	LUPUS ERITEMATOSO SISTÊMICO (LES)	descriptive<br>Field Annotation: @NOMISSING
348	info_antes_caso10<br>Show the field ONLY if: [caso_contr]='1' and ([doen_reuma]='10' or [predomina14]='10')	Antes dos sintomas da covid-19 Avaliação mais recente e disponível em prontuário, de no máximo 6 meses atrás	descriptive<br>Field Annotation: @NOMISSING
349	info_antes_contr10<br>Show the field ONLY if: [caso_contr]='2' and ([doen_reuma]='10' or [predomina14]='10')	Avaliação prévia Avaliação mais recente e disponível em prontuário, de no máximo 6 meses atrás	descriptive<br>Field Annotation: @NOMISSING

350	antes_grau_reuma10<br><br>Show the field ONLY if:<br>[doen_reuma]='10' or [predomina14]='10'	Qual o grau de atividade da doença?<br>*Indique um valor de 0 a 10 0 = ausência de atividade e 10= atividade intensa.*	text (integer, Min: 0, Max: 10)
351	antes_atividade10<br><br>Show the field ONLY if:<br>[doen_reuma]='10' or [predomina14]='10'	Assinale os envolvimentos de atividade da doença	checkbox<br>1 | antes_atividade10___1 | Articular<br>2 | antes_atividade10___2 | Cardíaco<br>3 | antes_atividade10___3 | Cutânea<br>4 | antes_atividade10___4 | Hematológica<br>5 | antes_atividade10___5 | Pulmonar<br>6 | antes_atividade10___6 | Renal<br>7 | antes_atividade10___7 | SNC<br>8 | antes_atividade10___8 | SNP<br>9 | antes_atividade10___9 | Vasculite<br>0 | antes_atividade10___0 | Nenhuma<br>777 | antes_atividade10___777 | Outros<br><br>Custom alignment: LV<br>Field Annotation: @NOMISSING @NONEOFTHEABOVE ='0'
352	antes_atividade_out10<br><br>Show the field ONLY if:<br>[antes_atividade10(777)]='1'	Qual outro envolvimentos de atividade da doença ?	text<br>Field Annotation: @NOMISSING
353	antes_sledai10<br><br>Show the field ONLY if:<br>[doen_reuma]='10' or [predomina14]='10'	SLEDAI-2K modificado mais recente disponível em prontuários	text (integer)
354	antes_saf10<br><br>Show the field ONLY if:<br>[doen_reuma]='10' or [predomina14]='10'	Tem SAF associada?<br>*SAF: Sindrome do Anticorpo antifosfolipede*	radio<br>1 | Sim<br>0 | Não<br><br>Custom alignment: LV<br>Field Annotation: @NOMISSING
355	antes_saf_trombo10<br><br>Show the field ONLY if:<br>[doen_reuma]='10' or [predomina14]='10'	Já teve evento trombótico prévio (arterial, venoso, obstétrico)?	radio<br>1 | Sim<br>0 | Não<br><br>Custom alignment: LV<br>Field Annotation: @NOMISSING
356	antes_saf_anticoag10<br><br>Show the field ONLY if:<br>[doen_reuma]='10' or [predomina14]='10'	Faz uso de anticoagulante oral ou HBPM cronicamente?	radio<br>1 | Sim<br>0 | Não<br><br>Custom alignment: LV<br>Field Annotation: @NOMISSING
357	antes_info_lab10<br><br>Show the field ONLY if:<br>[doen_reuma]='10' or [predomina14]='10'	LABORATÓRIO (último registro de prontuário)	descriptive<br>Field Annotation: @NOMISSING
358	antes_lab_fan10<br><br>Show the field ONLY if:<br>[doen_reuma]='10' or [predomina14]='10'	FAN	radio<br>0 | Negativo<br>1 | Positivo<br>999 | Não disponível<br><br>Custom alignment: LV<br>Field Annotation: @NOMISSING

359	antes_lab_fan_t10<br>Show the field ONLY if:<br>[antes_lab_fan10]='1'	Título	text
360	antes_lab_dna10<br>Show the field ONLY if:<br>[doen_reuma]='10' or [predomina14]='10'	Anti-DNA	radio<br>0 Negativo<br>1 Positivo<br>999 Não disponível<br>Custom alignment: LV<br>Field Annotation: @NOMISSING
361	antes_lab_cardio10<br>Show the field ONLY if:<br>[doen_reuma]='10' or [predomina14]='10'	Anti cardiolipina disponível?	radio<br>1 Sim<br>0 Não<br>Custom alignment: LV<br>Field Annotation: @NOMISSING
362	antes_lab_cardio_igg10<br>Show the field ONLY if:<br>[antes_lab_cardio10]='1'	IgG	text (integer)
363	antes_lab_cardio_igm10<br>Show the field ONLY if:<br>[antes_lab_cardio10]='1'	IgM	text (integer)
364	antes_lab_coag10<br>Show the field ONLY if:<br>[doen_reuma]='10' or [predomina14]='10'	Anti coagulante lúpico disponível?	radio<br>0 Negativo<br>1 Positivo<br>999 Não disponível<br>Custom alignment: LV<br>Field Annotation: @NOMISSING
365	antes_lab_glico10<br>Show the field ONLY if:<br>[antes_lab_cardio10]='1'	Anti-B2 glicoproteina disponível?	radio<br>1 Sim<br>0 Não<br>Custom alignment: LV<br>Field Annotation: @NOMISSING
366	antes_lab_glico_igg10<br>Show the field ONLY if:<br>[antes_lab_glico10]='1'	IgG	text (integer)
367	antes_lab_glico_igm10<br>Show the field ONLY if:<br>[antes_lab_glico10]='1'	IgM	text (integer)
368	antes_lab_coombs10<br>Show the field ONLY if:<br>[doen_reuma]='10' or [predomina14]='10'	Coombs direto	radio<br>0 Negativo<br>1 Positivo<br>999 Não disponível<br>Custom alignment: LV<br>Field Annotation: @NOMISSING
369	antes_lab_nucleo10<br>Show the field ONLY if:<br>[doen_reuma]='10' or [predomina14]='10'	Anti-nucleossomo disponível?	radio<br>1 Sim<br>0 Não<br>Custom alignment: LV<br>Field Annotation: @NOMISSING
370	antes_lab_nucleo_s10<br>Show the field ONLY if:<br>[antes_lab_nucleo10]='1'	Anti-nucleossomo<br>*Se negativo colocar ZERO*	text (integer)<br>Field Annotation: @NOMISSING

371	antes_lab_c3_10<br>Show the field ONLY if:<br>[doen_reuma]='10' or [predomina14]='10'	C3	text (integer)
372	antes_lab_c4_10<br>Show the field ONLY if:<br>[doen_reuma]='10' or [predomina14]='10'	C4	text (integer)
373	antes_lab_compl_10<br>Show the field ONLY if:<br>[doen_reuma]='10' or [predomina14]='10'	Complemento total	text (integer)
374	info_depois_caso10<br>Show the field ONLY if:<br>[caso_contr]='1' and ([doen_reuma]='10' or [predomina14]='10')	Após os sintomas da covid-19 Avaliação do momento da inclusão	descriptive<br>Field Annotation: @NOMISSING
375	info_depois_contr10<br>Show the field ONLY if:<br>[caso_contr]='2' and ([doen_reuma]='10' or [predomina14]='10')	Avaliação atual Avaliação do momento da inclusão	descriptive<br>Field Annotation: @NOMISSING
376	depois_piora_cov10<br>Show the field ONLY if:<br>[caso_contr]='1' and ([doen_reuma]='10' or [predomina14]='10')	Houve piora da atividade da doença depois dos sintomas de COVID-19?	radio<br>1 Sim<br>0 Não<br>888 Desconhecido<br>Custom alignment: LV<br>Field Annotation: @NOMISSING
377	depois_grau_reuma10<br>Show the field ONLY if:<br>[doen_reuma]='10' or [predomina14]='10'	Qual o grau de atividade da doença?<br>*Indique um valor de 0 a 10 0 = ausência de atividade e 10= atividade intensa.*	text (integer, Min: 0, Max: 10)
378	depois_sledai10<br>Show the field ONLY if:<br>[doen_reuma]='10' or [predomina14]='10'	SLEDAI-2K modificado mais recente disponível em prontuários	text (integer)
379	depois_mud_lab10<br>Show the field ONLY if:<br>[doen_reuma]='10' or [predomina14]='10'	Houve alguma mudança dos parâmetros laboratoriais específicos na visita atual?	radio<br>1 Sim<br>0 Não<br>999 Exames atuais não disponíveis<br>Custom alignment: LV<br>Field Annotation: @NOMISSING
380	depois_mud_lab_s10<br>Show the field ONLY if:<br>[depois_mud_lab10]='1'	Qual mudança?	checkbox<br>1 depois_mud_lab_s10___1 Anti-DNA<br>2 depois_mud_lab_s10___2 Anti-cardiolipina<br>3 depois_mud_lab_s10___3 Crioglobulinas<br>4 depois_mud_lab_s10___4 Complemento<br>Custom alignment: LV<br>Field Annotation: @NOMISSING

381	depois_dna10<br>Show the field ONLY if:<br>[depois_mud_lab_s10(1)]='1'	Anti-DNA	radio<br>1 Aumento título<br>2 Redução título<br>0 Inalterado<br>999 Não disponível<br>Custom alignment: LV<br>Field Annotation: @NOMISSING
382	depois_cardio10<br>Show the field ONLY if:<br>[depois_mud_lab_s10(2)]='1'	Anti-cardiolipina	radio<br>1 Aumento<br>2 Redução<br>0 Inalterado<br>999 Não disponível<br>Custom alignment: LV<br>Field Annotation: @NOMISSING
383	depois_criog10<br>Show the field ONLY if:<br>[depois_mud_lab_s10(3)]='1'	Crioglobulinas	radio<br>1 Aumento<br>2 Redução<br>0 Inalterado<br>999 Não disponível<br>Custom alignment: LV<br>Field Annotation: @NOMISSING
384	depois_compl10<br>Show the field ONLY if:<br>[depois_mud_lab_s10(4)]='1'	Complemento	radio<br>1 Consumo<br>0 Inalterado<br>999 Não disponível<br>Custom alignment: LV<br>Field Annotation: @NOMISSING
385	depois_trombo10<br>Show the field ONLY if:<br>[doen_reuma]='10' or [predomina14]='10'	Houve alguma nova manifestação trombótica?	radio<br>1 Sim<br>0 Não<br>Custom alignment: LV<br>Field Annotation: @NOMISSING
386	depois_trombo_s10<br>Show the field ONLY if:<br>[depois_trombo10]='1'	Qual?	radio<br>1 Arterial<br>2 Obstétrica<br>3 Venosa<br>Custom alignment: LV<br>Field Annotation: @NOMISSING
387	depois_piora_drim10<br>Show the field ONLY if:<br>[caso_contr]='1' and ([doen_reuma]='10' or [predomina14]='10')	Houve aparecimento ou piora de outra manifestação de DRIM?	radio<br>1 Sim<br>0 Não<br>Custom alignment: LV<br>Field Annotation: @NOMISSING
388	depois_piora_drim_s10<br>Show the field ONLY if:<br>[depois_piora_drim10]='1'	Qual?<br>*Aparecimento de outras manifestações imunomediadas não listadas acima ou não diretamente relacionada com a doença de base.*	text<br>Field Annotation: @NOMISSING
389	info_inicio_caso11<br>Show the field ONLY if:<br>([caso_contr]='1' or [caso_contr]='2') and [doen_reuma]='11'	MIOPATIA INFLAMATÓRIA (DERMATOMIOSITE)	descriptive<br>Field Annotation: @NOMISSING

390	info_antes_caso11<br>Show the field ONLY if: [caso_contr]='1' and ([doen_reuma]='11' or [predomina14]='11')	Antes dos sintomas da covid-19 Avaliação mais recente e disponível em prontuário, de no máximo 6 meses atrás	descriptive<br>Field Annotation: @NOMISSING
391	info_antes_contr11<br>Show the field ONLY if: [caso_contr]='2' and ([doen_reuma]='11' or [predomina14]='11')	Avaliação prévia Avaliação mais recente e disponível em prontuário, de no máximo 6 meses atrás	descriptive<br>Field Annotation: @NOMISSING
392	antes_grau_reuma11<br>Show the field ONLY if: [doen_reuma]='11' or [predomina14]='11'	Qual o grau de atividade da doença?<br>*Indique um valor de 0 a 10 0 = ausência de atividade e 10= atividade intensa.*	text (integer, Min: 0, Max: 10)
393	antes_mmt8_11<br>Show the field ONLY if: [doen_reuma]='11' or [predomina14]='11'	MMT-8 disponível?<br>*MMT: Manual Muscle Testing*	radio<br>1 Sim<br>0 Não<br>Custom alignment: LV<br>Field Annotation: @NOMISSING
394	antes_mmt8_s11<br>Show the field ONLY if: [antes_mmt8_11]='1'	MMT-8<br>*Apenas números. MMT: Manual Muscle Testing*	text (integer)
395	antes_haq11<br>Show the field ONLY if: [doen_reuma]='11' or [predomina14]='11'	HAQ disponível?<br>*HAQ: Health Assessment Questionnaire*	radio<br>1 Sim<br>0 Não<br>Custom alignment: LV<br>Field Annotation: @NOMISSING
396	antes_haq_s11<br>Show the field ONLY if: [antes_haq11]='1'	HAQ<br>*Apenas números, com duas casas decimais separados por PONTO. Exemplo: 2.34*	text (number)
397	antes_atividade11<br>Show the field ONLY if: [doen_reuma]='11' or [predomina14]='11'	Indique as manifestações clínicas que estavam em atividade	checkbox<br>1 antes_atividade11___1 Calcinose<br>2 antes_atividade11___2 Elevação de enzimas musculares<br>3 antes_atividade11___3 Gottron<br>4 antes_atividade11___4 Heliotropo<br>5 antes_atividade11___5 Musculo<br>6 antes_atividade11___6 Pele<br>7 antes_atividade11___7 Pulmão<br>8 antes_atividade11___8 Raynaud<br>Custom alignment: LV<br>Field Annotation: @NOMISSING
398	info_depois_caso11<br>Show the field ONLY if: [caso_contr]='1' and ([doen_reuma]='11' or [predomina14]='11')	Após os sintomas da covid-19 Avaliação do momento da inclusão	descriptive<br>Field Annotation: @NOMISSING
399	info_depois_contr11<br>Show the field ONLY if: [caso_contr]='2' and ([doen_reuma]='11' or [predomina14]='11')	Avaliação atual Avaliação do momento da inclusão	descriptive<br>Field Annotation: @NOMISSING

400	depois_piora_cov11<br><br>Show the field ONLY if:<br>[caso_contr]='1' and ([doen_reuma]='11' or [predomina14]='11')	Houve piora da atividade da doença depois dos sintomas de COVID-19?	radio<br>1 Sim<br>0 Não<br>888 Desconhecido<br><br>Custom alignment: LV<br>Field Annotation: @NOMISSING
401	depois_grau_reuma11<br><br>Show the field ONLY if:<br>[doen_reuma]='11' or [predomina14]='11'	Qual o grau de atividade da doença?<br>*Indique um valor de 0 a 10 0 = ausência de atividade e 10= atividade intensa.*	text (integer, Min: 0, Max: 10)
402	depois_info_ativ11<br><br>Show the field ONLY if:<br>[caso_contr]='1' and ([doen_reuma]='11' or [predomina14]='11')	Indique o grau de atividade da doença após a COVID-19	descriptive<br>Field Annotation: @NOMISSING
403	depois_ativ_calc11<br><br>Show the field ONLY if:<br>[caso_contr]='1' and ([doen_reuma]='11' or [predomina14]='11')	Calcinose	radio (Matrix)<br>0 Inalterado<br>2 Piorou<br>1 Melhorou<br><br>Field Annotation: @NOMISSING
404	depois_ativ_enz11<br><br>Show the field ONLY if:<br>[caso_contr]='1' and ([doen_reuma]='11' or [predomina14]='11')	Elevação de enzimas musculares	radio (Matrix)<br>0 Inalterado<br>2 Piorou<br>1 Melhorou<br><br>Field Annotation: @NOMISSING
405	depois_ativ_got11<br><br>Show the field ONLY if:<br>[caso_contr]='1' and ([doen_reuma]='11' or [predomina14]='11')	Gottron	radio (Matrix)<br>0 Inalterado<br>2 Piorou<br>1 Melhorou<br><br>Field Annotation: @NOMISSING
406	depois_ativ_heli11<br><br>Show the field ONLY if:<br>[caso_contr]='1' and ([doen_reuma]='11' or [predomina14]='11')	Heliotropo	radio (Matrix)<br>0 Inalterado<br>2 Piorou<br>1 Melhorou<br><br>Field Annotation: @NOMISSING
407	depois_ativ_musc11<br><br>Show the field ONLY if:<br>[caso_contr]='1' and ([doen_reuma]='11' or [predomina14]='11')	Musculo	radio (Matrix)<br>0 Inalterado<br>2 Piorou<br>1 Melhorou<br><br>Field Annotation: @NOMISSING
408	depois_ativ_pele11<br><br>Show the field ONLY if:<br>[caso_contr]='1' and ([doen_reuma]='11' or [predomina14]='11')	Pele	radio (Matrix)<br>0 Inalterado<br>2 Piorou<br>1 Melhorou<br><br>Field Annotation: @NOMISSING

409	depois_ativ_pulm11<br>Show the field ONLY if: [caso_contr]='1' and ([doen_reuma]='11' or [predomina14]='11')	Pulmão	radio (Matrix)<br>0 Inalterado<br>2 Piorou<br>1 Melhorou<br>Field Annotation: @NOMISSING
410	depois_ativ_rayn11<br>Show the field ONLY if: [caso_contr]='1' and ([doen_reuma]='11' or [predomina14]='11')	Raynaud	radio (Matrix)<br>0 Inalterado<br>2 Piorou<br>1 Melhorou<br>Field Annotation: @NOMISSING
411	depois_piora_drim11<br>Show the field ONLY if: [caso_contr]='1' and ([doen_reuma]='11' or [predomina14]='11')	Houve aparecimento ou piora de outra manifestação de DRIM?	radio<br>1 Sim<br>0 Não<br>Custom alignment: LV<br>Field Annotation: @NOMISSING
412	depois_piora_drim_s11<br>Show the field ONLY if: [depois_piora_drim11]='1'	Qual?<br>*Aparecimento de outras manifestações imunomediadas não listadas acima ou não diretamente relacionada com a doença de base.*	text<br>Field Annotation: @NOMISSING
413	info_antes_caso12<br>Show the field ONLY if: [caso_contr]='1' and [doen_reuma]='12'	SARCOIDOSE - Antes dos sintomas da covid-19 Avaliação mais recente e disponível em prontuário, de no máximo 6 meses atrás	descriptive<br>Field Annotation: @NOMISSING
414	info_antes_contr12<br>Show the field ONLY if: [caso_contr]='2' and [doen_reuma]='12'	SARCOIDOSE - Avaliação prévia Avaliação mais recente e disponível em prontuário, de no máximo 6 meses atrás	descriptive<br>Field Annotation: @NOMISSING
415	antes_grau_reuma12<br>Show the field ONLY if: [doen_reuma]='12'	Qual o grau de atividade da doença?<br>*Indique um valor de 0 a 10 0 = ausência de atividade e 10= atividade intensa.*	text (integer, Min: 0, Max: 10)
416	antes_atividade12<br>Show the field ONLY if: [doen_reuma]='12'	Assinale as manifestações clínicas que estavam em atividade	checkbox<br>1 antes_atividade12___1 Cardíaco<br>2 antes_atividade12___2 Intestinal<br>3 antes_atividade12___3 Linfadenopatia<br>4 antes_atividade12___4 Neurológico<br>5 antes_atividade12___5 Ocular<br>6 antes_atividade12___6 Osteoarticular<br>7 antes_atividade12___7 Pele/subcutâneo<br>8 antes_atividade12___8 Pulmonar<br>0 antes_atividade12___0 Nenhuma<br>777 antes_atividade12___777 Outros<br>Custom alignment: LV<br>Field Annotation: @NOMISSING @NONEOFTHEABOVE ='0'
417	antes_atividade_out12<br>Show the field ONLY if: [antes_atividade12(777)]='1'	Qual outro envolvimento da doença?	text<br>Field Annotation: @NOMISSING
418	info_depois_caso12<br>Show the field ONLY if: [caso_contr]='1' and [doen_reuma]='12'	Após os sintomas da covid-19 Avaliação do momento da inclusão	descriptive<br>Field Annotation: @NOMISSING

419	info_depois_contr12<br>Show the field ONLY if:<br>[caso_contr]='2' and [doen_reuma]='12'	Avaliação atual Avaliação do momento da inclusão	descriptive<br>Field Annotation: @NOMISSING
420	depois_grau_reuma12<br>Show the field ONLY if:<br>[doen_reuma]='12'	Qual o grau de atividade da doença?<br>*Indique um valor de 0 a 10 0 = ausência de atividade e 10= atividade intensa.*	text (integer, Min: 0, Max: 10)
421	depois_piora_cov12<br>Show the field ONLY if:<br>[caso_contr]='1' and [doen_reuma]='12'	Houve piora da atividade da doença depois dos sintomas de COVID-19?	radio<br>1 Sim<br>0 Não<br>888 Desconhecido<br>Custom alignment: LV<br>Field Annotation: @NOMISSING
422	depois_atividade12<br>Show the field ONLY if:<br>[depois_piora_cov12]='1'	Assinale qual(is) manifestação(ões) da atividade(s) da doença(s)	checkbox<br>1 depois_atividade12___1 Cardíaco<br>2 depois_atividade12___2 Intestinal<br>3 depois_atividade12___3 Linfadenopatia<br>4 depois_atividade12___4 Neurológico<br>5 depois_atividade12___5 Ocular<br>6 depois_atividade12___6 Osteoarticular<br>7 depois_atividade12___7 Pele/subcutâneo<br>8 depois_atividade12___8 Pulmonar<br>0 depois_atividade12___0 Nenhuma<br>777 depois_atividade12___777 Outros<br>Custom alignment: LV<br>Field Annotation: @NOMISSING @NONEOFTHEABOVE ='0'
423	depois_atividade_out12<br>Show the field ONLY if:<br>[depois_atividade12(777)]='1'	Qual outro envolvimento da doença?	text<br>Field Annotation: @NOMISSING
424	depois_trombo12<br>Show the field ONLY if:<br>[doen_reuma]='12'	Houve evento tromboembólico?	radio<br>1 Sim<br>0 Não<br>Custom alignment: LV<br>Field Annotation: @NOMISSING
425	depois_trombo_s12<br>Show the field ONLY if:<br>[depois_trombo12]='1'	Em qual sítio?	checkbox<br>1 depois_trombo_s12___1 AVC<br>2 depois_trombo_s12___2 Oclusão Arterial Aguda<br>3 depois_trombo_s12___3 Retina<br>4 depois_trombo_s12___4 Tromboembolismo Pulmonar<br>5 depois_trombo_s12___5 Tromboflebite superficial<br>6 depois_trombo_s12___6 Trombose Venosa Profunda<br>777 depois_trombo_s12___777 Outros<br>Custom alignment: LV<br>Field Annotation: @NOMISSING
426	depois_trombo_s_out12<br>Show the field ONLY if:<br>[depois_trombo_s12(777)]='1'	Qual outro sítio?	text<br>Field Annotation: @NOMISSING

427	depois_piora_drim12<br>Show the field ONLY if: [caso_contr]='1' and [doen_reuma]='12'	Houve aparecimento ou piora de outra manifestação de DRIM?	radio<br>1 Sim<br>0 Não<br>Custom alignment: LV<br>Field Annotation: @NOMISSING
428	depois_piora_drim_s12<br>Show the field ONLY if: [depois_piora_drim12]='1'	Qual?<br>*Aparecimento de outras manifestações imunomediadas não listadas acima ou não diretamente relacionada com a doença de base.*	text<br>Field Annotation: @NOMISSING
429	info_inicio_caso13<br>Show the field ONLY if: ([caso_contr]='1' or [caso_contr]='2') and [doen_reuma]='13'	SÍNDROME DE SJÖGREN PRIMÁRIA	descriptive<br>Field Annotation: @NOMISSING
430	info_carac_caso13<br>Show the field ONLY if: [caso_contr]='1' and ([doen_reuma]='13' or [predomina14]='13')	Características da doença	descriptive<br>Field Annotation: @NOMISSING
431	info_carac_contr13<br>Show the field ONLY if: [caso_contr]='2' and ([doen_reuma]='13' or [predomina14]='13')	Características da doença	descriptive<br>Field Annotation: @NOMISSING
432	carac_lab_fan13<br>Show the field ONLY if: [doen_reuma]='13' or [predomina14]='13'	FAN (qualquer data)	radio<br>1 Positivo<br>0 Negativo<br>999 Não disponível<br>Custom alignment: LV<br>Field Annotation: @NOMISSING
433	carac_lab_fan_t13<br>Show the field ONLY if: [carac_lab_fan13]='1'	Título FAN	text<br>Field Annotation: @NOMISSING
434	carac_lab_padrao13<br>Show the field ONLY if: [carac_lab_fan13]='1'	Padrão	text
435	carac_anti_ro13<br>Show the field ONLY if: [doen_reuma]='13' or [predomina14]='13'	Anti- Ro (qualquer data)	radio<br>1 Positivo<br>0 Negativo<br>999 Não disponível<br>Custom alignment: LV<br>Field Annotation: @NOMISSING
436	carac_info_lab13<br>Show the field ONLY if: [doen_reuma]='13' or [predomina14]='13'	Laboratório	descriptive<br>Field Annotation: @NOMISSING
437	carac_lab_fator13<br>Show the field ONLY if: [doen_reuma]='13' or [predomina14]='13'	Fator reumatoide (qualquer data)<br>*Baixo título: até três vezes o valor de referência Altos títulos: mais de três vezes o valor de referência*	radio<br>1 Positivo<br>0 Negativo<br>999 Não disponível<br>Custom alignment: LV<br>Field Annotation: @NOMISSING
438	info_antes_caso13<br>Show the field ONLY if: [caso_contr]='1' and [doen_reuma]='13'	Antes dos sintomas da covid-19 Avaliação mais recente e disponível em prontuário, de no máximo 6 meses atrás	descriptive<br>Field Annotation: @NOMISSING

439	info_antes_contr13<br><br>Show the field ONLY if:<br>[caso_contr]='2' and [doen_reuma]='13'	Avaliação prévia Avaliação mais recente e disponível em prontuário, de no máximo 6 meses atrás	descriptive<br>Field Annotation: @NOMISSING
440	antes_grau_reuma123<br><br>Show the field ONLY if:<br>[doen_reuma]='13' or [predomina14]='13'	Qual o grau de atividade da doença?<br>*Indique um valor de 0 a 10 0 = ausência de atividade e 10= atividade intensa.*	text (integer, Min: 0, Max: 10)<br>Field Annotation: @NOMISSING
441	antes_atividade13<br><br>Show the field ONLY if:<br>[doen_reuma]='13' or [predomina14]='13'	Assinale o tipo de comprometimento sistemico da doença	checkbox<br>1 | antes_atividade13___1 | Articular<br>2 | antes_atividade13___2 | Boca seca<br>3 | antes_atividade13___3 | Glandular<br>4 | antes_atividade13___4 | Hematológico<br>5 | antes_atividade13___5 | Neuropatia periférica<br>6 | antes_atividade13___6 | Olho seco<br>7 | antes_atividade13___7 | Pele<br>8 | antes_atividade13___8 | Pulmonar<br>9 | antes_atividade13___9 | Renal<br>10 | antes_atividade13___10 | Sistema nervoso central<br>11 | antes_atividade13___11 | Vascular<br>0 | antes_atividade13___0 | Nenhuma<br>777 | antes_atividade13___777 | Outros<br><br>Custom alignment: LV<br>Field Annotation: @NOMISSING @NONEOFTHEABOVE ='0'
442	antes_atividade_out13<br><br>Show the field ONLY if:<br>[antes_atividade13(777)]='1'	Qual outro comprometimento sistemico da doença?	text<br>Field Annotation: @NOMISSING
443	antes_essdai13<br><br>Show the field ONLY if:<br>[doen_reuma]='13' or [predomina14]='13'	ESSDAI disponível?<br>*ESSDAI: EULAR Sjogren's Syndrome Disease Activity Index*	radio<br>1 | Sim<br>0 | Não<br><br>Custom alignment: LV<br>Field Annotation: @NOMISSING
444	antes_essdai_s13<br><br>Show the field ONLY if:<br>[antes_essdai13]='1'	ESSDAI<br>*Apenas números. ESSDAI: EULAR Sjogren's Syndrome Disease Activity Index*	text (integer)
445	antes_lab_c3_13<br><br>Show the field ONLY if:<br>[doen_reuma]='13' or [predomina14]='13'	C3	radio<br>1 | Baixo<br>0 | Normal<br>999 | Não disponível<br><br>Custom alignment: LV<br>Field Annotation: @NOMISSING
446	antes_lab_c3_s13<br><br>Show the field ONLY if:<br>[antes_lab_c3_13]='1'	C3 valor	text (integer)
447	antes_lab_c4_13<br><br>Show the field ONLY if:<br>[doen_reuma]='13' or [predomina14]='13'	C4	radio<br>1 | Baixo<br>0 | Normal<br>999 | Não disponível<br><br>Custom alignment: LV<br>Field Annotation: @NOMISSING

448	antes_lab_c4_s13<br><br>Show the field ONLY if:<br>[antes_lab_c4_13]='1'	C4 valor	text (integer)
449	antes_lab_ch50_13<br><br>Show the field ONLY if:<br>[doen_reuma]='13' or [predomina14]='13'	CH50	text (integer)
450	antes_eletro13<br><br>Show the field ONLY if:<br>[doen_reuma]='13' or [predomina14]='13'	Eletroforese de proteínas (gamaglobulina)<br>*g/L*	text (integer)
451	antes_crioglo13<br><br>Show the field ONLY if:<br>[doen_reuma]='13' or [predomina14]='13'	Crioglobulinas	radio<br>1 Positivo<br>0 Negativo<br>999 Não disponível<br><br>Custom alignment: LV<br>Field Annotation: @NOMISSING
452	antes_crioglo_s13<br><br>Show the field ONLY if:<br>[antes_crioglo13]='1'	Crioglobulinas valor	text
453	info_depois_caso13<br><br>Show the field ONLY if:<br>[caso_contr]='1' and ([doen_reuma]='13' or [predomina14]='13')	Após os sintomas da covid-19 Avaliação do momento da inclusão	descriptive<br>Field Annotation: @NOMISSING
454	info_depois_contr13<br><br>Show the field ONLY if:<br>[caso_contr]='2' and ([doen_reuma]='13' or [predomina14]='13')	Avaliação atual Avaliação do momento da inclusão	descriptive<br>Field Annotation: @NOMISSING
455	depois_piora_cov13<br><br>Show the field ONLY if:<br>[caso_contr]='1' and ([doen_reuma]='13' or [predomina14]='13')	Houve piora da atividade da doença depois dos sintomas de COVID-19?	radio<br>1 Sim<br>0 Não<br>888 Desconhecido<br><br>Custom alignment: LV<br>Field Annotation: @NOMISSING
456	depois_grau_reuma13<br><br>Show the field ONLY if:<br>[doen_reuma]='13' or [predomina14]='13'	Qual o grau de atividade da doença?<br>*Indique um valor de 0 a 10 0 = ausência de atividade e 10= atividade intensa.*	text (integer, Min: 0, Max: 10)
457	depois_info_ativ13<br><br>Show the field ONLY if:<br>[doen_reuma]='13' or [predomina14]='13'	Em relação às manifestações clínicas iniciais, indique o grau de atividade neste momento	descriptive<br>Field Annotation: @NOMISSING
458	depois_ativ_artrit13<br><br>Show the field ONLY if:<br>[doen_reuma]='13' or [predomina14]='13'	Artrite	radio (Matrix)<br>0 Inalterado<br>2 Piorou<br>1 Melhorou<br><br>Field Annotation: @NOMISSING
459	depois_ativ_boca13<br><br>Show the field ONLY if:<br>[doen_reuma]='13' or [predomina14]='13'	Boca seca	radio (Matrix)<br>0 Inalterado<br>2 Piorou<br>1 Melhorou<br><br>Field Annotation: @NOMISSING

460	depois_ativ_gland13<br>Show the field ONLY if:<br>[doen_reuma]='13' or [predomina14]='13'	Glândular	radio (Matrix)<br>0 Inalterado<br>2 Piorou<br>1 Melhorou<br>Field Annotation: @NOMISSING
461	depois_ativ_hemat13<br>Show the field ONLY if:<br>[doen_reuma]='13' or [predomina14]='13'	Hematológico	radio (Matrix)<br>0 Inalterado<br>2 Piorou<br>1 Melhorou<br>Field Annotation: @NOMISSING
462	depois_ativ_neuro13<br>Show the field ONLY if:<br>[doen_reuma]='13' or [predomina14]='13'	Neuropatia periférica	radio (Matrix)<br>0 Inalterado<br>2 Piorou<br>1 Melhorou<br>Field Annotation: @NOMISSING
463	depois_ativ_olho13<br>Show the field ONLY if:<br>[doen_reuma]='13' or [predomina14]='13'	Olho seco	radio (Matrix)<br>0 Inalterado<br>2 Piorou<br>1 Melhorou<br>Field Annotation: @NOMISSING
464	depois_ativ_pele13<br>Show the field ONLY if:<br>[doen_reuma]='13' or [predomina14]='13'	Pele	radio (Matrix)<br>0 Inalterado<br>2 Piorou<br>1 Melhorou<br>Field Annotation: @NOMISSING
465	depois_ativ_pulm13<br>Show the field ONLY if:<br>[doen_reuma]='13' or [predomina14]='13'	Pulmonar	radio (Matrix)<br>0 Inalterado<br>2 Piorou<br>1 Melhorou<br>Field Annotation: @NOMISSING
466	depois_ativ_renal13<br>Show the field ONLY if:<br>[doen_reuma]='13' or [predomina14]='13'	Renal	radio (Matrix)<br>0 Inalterado<br>2 Piorou<br>1 Melhorou<br>Field Annotation: @NOMISSING
467	depois_ativ_nerv13<br>Show the field ONLY if:<br>[doen_reuma]='13' or [predomina14]='13'	Sistema nervoso central	radio (Matrix)<br>0 Inalterado<br>2 Piorou<br>1 Melhorou<br>Field Annotation: @NOMISSING
468	depois_ativ_vasc13<br>Show the field ONLY if:<br>[doen_reuma]='13' or [predomina14]='13'	Vascular	radio (Matrix)<br>0 Inalterado<br>2 Piorou<br>1 Melhorou<br>Field Annotation: @NOMISSING

469	depois_essdai_s13<br>Show the field ONLY if:<br>[doen_reuma]='13' or [predomina14]='13'	ESSDAI atual<br>*Apenas números ESSDAI: EULAR Sjogren's Syndrome Disease Activity Index*	text (integer)
470	depois_piora_drim13<br>Show the field ONLY if:<br>[caso_contr]='1' and ([doen_reuma]='13' or [predomina14]='13')	Houve aparecimento ou piora de outra manifestação de DRIM?	radio<br>1 Sim<br>0 Não<br>Custom alignment: LV<br>Field Annotation: @NOMISSING
471	depois_piora_drim_s13<br>Show the field ONLY if:<br>[depois_piora_drim13]='1'	Qual?<br>*Aparecimento de outras manifestações imunomediadas não listadas acima ou não diretamente relacionada com a doença de base.*	text<br>Field Annotation: @NOMISSING
472	info_antes_caso14<br>Show the field ONLY if:<br>([caso_contr]='1' or [caso_contr]='2') and [doen_reuma]='14'	SÍNDROME DE SOBREPOSIÇÃO Características da doença	descriptive<br>Field Annotation: @NOMISSING
473	predomina14<br>Show the field ONLY if:<br>([caso_contr]='1' or [caso_contr]='2') and [doen_reuma]='14'	Assinale qual a doença predominante	radio<br>4 Artrite reumatoide<br>8 Esclerose sistêmica<br>10 Lupus eritematoso sistêmico<br>11 Miopatia inflamatória<br>13 Síndrome de Sjögren<br>Custom alignment: LV<br>Field Annotation: @NOMISSING
474	info_antes_caso15<br>Show the field ONLY if:<br>[caso_contr]='1' and [doen_reuma]='15'	SÍNDROME DO ANTICORPO ANTIFOSFOLÍPIDE PRIMÁRIA Características da doença	descriptive<br>Field Annotation: @NOMISSING
475	info_antes_contr15<br>Show the field ONLY if:<br>[caso_contr]='2' and [doen_reuma]='15'	SÍNDROME DO ANTICORPO ANTIFOSFOLÍPIDE PRIMÁRIA Características da doença	descriptive<br>Field Annotation: @NOMISSING
476	antes_trombo15<br>Show the field ONLY if:<br>[doen_reuma]='15'	Assinale o envolvimento trombótico da doença (marque todos que se aplicam):	checkbox<br>1 antes_trombo15___1 Ambos<br>2 antes_trombo15___2 Arterial<br>3 antes_trombo15___3 Obstétrico<br>4 antes_trombo15___4 Venoso<br>0 antes_trombo15___0 Sem evento<br>Custom alignment: LV<br>Field Annotation: @NOMISSING @NONEOFTHEABOVE='0'
477	antes_topog_tromb15<br>Show the field ONLY if:<br>[doen_reuma]='15'	Topografia do evento trombótico (marque todos que se aplicam):	checkbox<br>1 antes_topog_tromb15___1 AVC<br>2 antes_topog_tromb15___2 Oclusão Arterial Aguda<br>3 antes_topog_tromb15___3 Retina<br>4 antes_topog_tromb15___4 Tromboembolismo Pulmonar<br>5 antes_topog_tromb15___5 Tromboflebite superficial<br>6 antes_topog_tromb15___6 Trombose Venosa Profunda<br>777 antes_topog_tromb15___777 Outros<br>Custom alignment: LV<br>Field Annotation: @NOMISSING

478	antes_topog_tromb_out15<br><br>Show the field ONLY if:<br>[antes_topog_tromb15(777)]<br>='1'	Qual outra topografia do evento trombótico ?	text<br>Field Annotation: @NOMISSING
479	antes_anticoag15<br><br>Show the field ONLY if:<br>[doen_reuma]='15'	Faz uso de anticoagulante oral ou HBPM cronicamente?	radio<br>1 Sim<br>0 Não<br><br>Custom alignment: LV<br>Field Annotation: @NOMISSING
480	antes_anticoag_s15<br><br>Show the field ONLY if:<br>[doen_reuma]='15'	Estava no alvo (últimos 3 meses)?	radio<br>1 Sim<br>0 Não<br><br>Custom alignment: LV<br>Field Annotation: @NOMISSING
481	antes_infobio_lab15<br><br>Show the field ONLY if:<br>[doen_reuma]='15'	LABORATÓRIO (último registro de prontuário)	descriptive<br>Field Annotation: @NOMISSING
482	antes_lab_fan15<br><br>Show the field ONLY if:<br>[doen_reuma]='15'	FAN	radio<br>0 Negativo<br>1 Positivo<br>999 Não disponível<br><br>Custom alignment: LV<br>Field Annotation: @NOMISSING
483	antes_lab_fan_t15<br><br>Show the field ONLY if:<br>[antes_lab_fan15]='1'	Título	text
484	antes_lab_cardio15<br><br>Show the field ONLY if:<br>[doen_reuma]='15'	Anti cardiolipina disponível?	radio<br>1 Sim<br>0 Não<br><br>Custom alignment: LV<br>Field Annotation: @NOMISSING
485	antes_lab_cardio_igg15<br><br>Show the field ONLY if:<br>[antes_lab_cardio15]='1'	IgG	text (integer)
486	antes_lab_cardio_igm15<br><br>Show the field ONLY if:<br>[antes_lab_cardio15]='1'	IgM	text (integer)
487	antes_lab_coag15<br><br>Show the field ONLY if:<br>[doen_reuma]='15'	Anti coagulante lúpico?	radio<br>0 Negativo<br>1 Positivo<br>999 Não disponível<br><br>Custom alignment: LV<br>Field Annotation: @NOMISSING
488	antes_lab_glico15<br><br>Show the field ONLY if:<br>[doen_reuma]='15'	Anti-B2 glicoproteina disponível?	radio<br>1 Sim<br>0 Não<br><br>Custom alignment: LV<br>Field Annotation: @NOMISSING
489	antes_lab_glico_igg15<br><br>Show the field ONLY if:<br>[antes_lab_glico15]='1'	IgG	text (integer)

490	antes_lab_glico_igm15<br>Show the field ONLY if:<br>[antes_lab_glico15]='1'	IgM	text (integer)
491	info_depois_caso15<br>Show the field ONLY if:<br>[caso_contr]='1' and [doen_reuma]='15'	Após os sintomas da covid-19 Avaliação do momento da inclusão	descriptive<br>Field Annotation: @NOMISSING
492	info_depois_contr15<br>Show the field ONLY if:<br>[caso_contr]='2' and [doen_reuma]='15'	Avaliação atual Avaliação do momento da inclusão	descriptive<br>Field Annotation: @NOMISSING
493	depois_piora_cov15<br>Show the field ONLY if:<br>[caso_contr]='1' and [doen_reuma]='15'	Houve piora da atividade da doença depois dos sintomas de COVID-19?	radio<br>1 Sim<br>0 Não<br>888 Desconhecido<br>Custom alignment: LV<br>Field Annotation: @NOMISSING
494	depois_trombo15<br>Show the field ONLY if:<br>[doen_reuma]='15'	Apresentou novo evento tromboembólico?	radio<br>1 Sim<br>0 Não<br>Custom alignment: LV<br>Field Annotation: @NOMISSING
495	depois_trombo_sitio15<br>Show the field ONLY if:<br>[depois_trombo15]='1'	Em qual sítio?	checkbox<br>1 depois_trombo_sitio15___1 AVC<br>2 depois_trombo_sitio15___2 Oclusão Arterial Aguda<br>3 depois_trombo_sitio15___3 Retina<br>4 depois_trombo_sitio15___4 Tromboembolismo Pulmonar<br>5 depois_trombo_sitio15___5 Tromboflebite superficial<br>6 depois_trombo_sitio15___6 Trombose Venosa Profunda<br>777 depois_trombo_sitio15___777 Outros<br>Custom alignment: LV<br>Field Annotation: @NOMISSING
496	depois_trombo_sitio_out15<br>Show the field ONLY if:<br>[depois_trombo_sitio15(777)]='1'	Qual outro sítio?	text<br>Field Annotation: @NOMISSING
497	depois_lab_mudanca15<br>Show the field ONLY if:<br>[doen_reuma]='15'	Houve alguma mudança dos parâmetros laboratoriais específicos na visita atual?	radio<br>1 Sim<br>0 Não<br>999 Não tenho exames atuais<br>Custom alignment: LV<br>Field Annotation: @NOMISSING
498	depois_lab_anticardio15<br>Show the field ONLY if:<br>[depois_lab_mudanca15]='1'	Anticardiolipina	radio<br>0 Inalterado<br>2 Piorou<br>1 Melhorou<br>999 Não disponível<br>Custom alignment: LV<br>Field Annotation: @NOMISSING

499	depois_lab_antib2_15<br>Show the field ONLY if:<br>[depois_lab_mudanca15]='1'	Anti-B2 glicoproteína	radio<br>0 Inalterado<br>2 Piorou<br>1 Melhorou<br>999 Não disponível<br><br>Custom alignment: LV<br>Field Annotation: @NOMISSING
500	depois_lab_anticoag15<br>Show the field ONLY if:<br>[depois_lab_mudanca15]='1'	Anticoagulante lúpico	radio<br>0 Inalterado<br>2 Piorou<br>1 Melhorou<br>999 Não disponível<br><br>Custom alignment: LV<br>Field Annotation: @NOMISSING
501	depois_piora_drim15<br>Show the field ONLY if:<br>[caso_contr]='1' and [doen_reuma]='15'	Houve aparecimento ou piora de outra manifestação de DRIM?	radio<br>1 Sim<br>0 Não<br><br>Custom alignment: LV<br>Field Annotation: @NOMISSING
502	depois_piora_drim_s15<br>Show the field ONLY if:<br>[depois_piora_drim15]='1'	Qual?<br>*Aparecimento de outras manifestações imunomediadas não listadas acima ou não diretamente relacionada com a doença de base.*	text<br>Field Annotation: @NOMISSING
503	info_carac16<br>Show the field ONLY if:<br>([caso_contr]='1' or [caso_contr]='2') and [doen_reuma]='16'	16. VASCULITES Características da doença	descriptive<br>Field Annotation: @NOMISSING
504	carac_acometim16<br>Show the field ONLY if:<br>([caso_contr]='1' or [caso_contr]='2') and [doen_reuma]='16'	Selecione o acometimento	radio<br>1 Pequenos e Médios Vasos<br>2 Acometimento de Grandes Vasos<br><br>Custom alignment: LV<br>Field Annotation: @NOMISSING
505	info_antes_caso16<br>Show the field ONLY if:<br>[caso_contr]='1' and [doen_reuma]='16'	VASCULITES - Antes dos sintomas da covid-19 Avaliação mais recente e disponível em prontuário, de no máximo 6 meses atrás	descriptive<br>Field Annotation: @NOMISSING
506	info_antes_contr16<br>Show the field ONLY if:<br>[caso_contr]='2' and [doen_reuma]='16'	VASCULITES Características da doença	descriptive<br>Field Annotation: @NOMISSING
507	antes_peq_titulo16<br>Show the field ONLY if:<br>[carac_acometim16]='1'	Assinale qual o diagnóstico	radio<br>1 Doença por anti-GBM<br>2 GEPA (Granulomatose Eosinofílica com Poliangeíte)<br>3 GPA (Granulomatose com Poliangeíte)<br>4 PAM (Poliangeíte Microscópica)<br>5 PAN (Poliarterite Nodosa)<br>6 PAN cutânea<br>7 Vasculite por IgA<br><br>Custom alignment: LV<br>Field Annotation: @NOMISSING
508	antes_peq_grau_reuma16<br>Show the field ONLY if:<br>[carac_acometim16]='1'	Qual o grau de atividade da doença?<br>*Indique um valor de 0 a 10 0 = ausência de atividade e 10= atividade intensa.*	text (integer, Min: 0, Max: 10)

509	antes_peq_bvas16<br><br>Show the field ONLY if:<br>[carac_acometim16]='1'	BVAS (versão 3)<br>*Apenas números. BVAS: Birmingham Vasculitis Activity Score*	text (integer)
510	antes_peq_pan16<br><br>Show the field ONLY if:<br>[carac_acometim16]='1'	Five-Factor Score (PAN, GEPA e GPA):<br>*PAN: Poliarterite Nodosa GEPA: Granulomatose Eosinofílica com Poliangeíte GPA: Granulomatose com Poliangeíte*	text (integer)
511	antes_peq_ativid16<br><br>Show the field ONLY if:<br>[carac_acometim16]='1'	Assinale as manifestações clínicas em atividade:	checkbox<br>1 antes_peq_ativid16___1 Articular<br>2 antes_peq_ativid16___2 Cardiovascular<br>3 antes_peq_ativid16___3 Cutânea<br>4 antes_peq_ativid16___4 Gastrointestinal<br>5 antes_peq_ativid16___5 Ocular<br>6 antes_peq_ativid16___6 Pulmonar<br>7 antes_peq_ativid16___7 Renal<br>8 antes_peq_ativid16___8 SN periférico<br>9 antes_peq_ativid16___9 SNC<br>10 antes_peq_ativid16___10 Vias aéreas superiores<br>0 antes_peq_ativid16___0 Nenhuma<br><br>Custom alignment: LV<br>Field Annotation: @NOMISSING<br>@NONEOFTHEABOVE='0'
512	antes_anca_c16<br><br>Show the field ONLY if:<br>[carac_acometim16]='1'	ANCA-C (IMF) Qualquer data	radio<br>1 Positivo<br>0 Negativo<br>999 Não disponível<br><br>Custom alignment: LV<br>Field Annotation: @NOMISSING
513	antes_anca_c_valor16<br><br>Show the field ONLY if:<br>[carac_acometim16]='1'	ANCA-C (IMF) Qualquer data valor	text (integer)
514	antes_anca_mes_c16<br><br>Show the field ONLY if:<br>[carac_acometim16]='1'	ANCA-C (IMF) Últimos 6 meses	radio<br>1 Positivo<br>0 Negativo<br>999 Não disponível<br><br>Custom alignment: LV<br>Field Annotation: @NOMISSING
515	antes_anca_mes_c_valor16<br><br>Show the field ONLY if:<br>[carac_acometim16]='1'	ANCA-C (IMF) Últimos 6 meses valor	text (integer)
516	antes_anca_p16<br><br>Show the field ONLY if:<br>[carac_acometim16]='1'	ANCA-P (IMF) Qualquer data	radio<br>1 Positivo<br>0 Negativo<br>999 Não disponível<br><br>Custom alignment: LV<br>Field Annotation: @NOMISSING
517	antes_anca_p_valor16<br><br>Show the field ONLY if:<br>[carac_acometim16]='1'	ANCA-P (IMF) Qualquer data valor	text (integer)

518	antes_anca_mes_p16<br><br>Show the field ONLY if:<br>[carac_acometim16]='1'	ANCA-P (IMF) Últimos 6 meses	radio<br>1 Positivo<br>0 Negativo<br>999 Não disponível<br><br>Custom alignment: LV<br>Field Annotation: @NOMISSING
519	antes_anca_mes_p_valor16<br><br>Show the field ONLY if:<br>[carac_acometim16]='1'	ANCA-P (IMF) Últimos 6 meses valor	text (integer)
520	antes_anca_pr16<br><br>Show the field ONLY if:<br>[carac_acometim16]='1'	ANCA-PR3 Qualquer data	radio<br>1 Positivo<br>0 Negativo<br>999 Não disponível<br><br>Custom alignment: LV<br>Field Annotation: @NOMISSING
521	antes_anca_pr_valor16<br><br>Show the field ONLY if:<br>[carac_acometim16]='1'	ANCA-PR3 Qualquer data valor	text (integer)
522	antes_anca_mes_pr16<br><br>Show the field ONLY if:<br>[carac_acometim16]='1'	ANCA-PR3 Últimos 6 meses	radio<br>1 Positivo<br>0 Negativo<br>999 Não disponível<br><br>Custom alignment: LV<br>Field Annotation: @NOMISSING
523	antes_anca_mes_pr_valor16<br><br>Show the field ONLY if:<br>[carac_acometim16]='1'	ANCA-PR3 Últimos 6 meses valor	text (integer)
524	antes_anca_mp16<br><br>Show the field ONLY if:<br>[carac_acometim16]='1'	ANCA-MPO Qualquer data	radio<br>1 Positivo<br>0 Negativo<br>999 Não disponível<br><br>Custom alignment: LV<br>Field Annotation: @NOMISSING
525	antes_anca_mp_valor16<br><br>Show the field ONLY if:<br>[carac_acometim16]='1'	ANCA-MPO Qualquer data valor	text (integer)
526	antes_anca_mes_mp16<br><br>Show the field ONLY if:<br>[carac_acometim16]='1'	ANCA-MPO Últimos 6 meses	radio<br>1 Positivo<br>0 Negativo<br>999 Não disponível<br><br>Custom alignment: LV<br>Field Annotation: @NOMISSING
527	antes_anca_mes_mp_valor16<br><br>Show the field ONLY if:<br>[carac_acometim16]='1'	ANCA-MPO Últimos 6 meses valor	text (integer)
528	antes_gde_titulo16<br><br>Show the field ONLY if:<br>[carac_acometim16]='2'	Assinale qual o diagnóstico	radio<br>1 Arterite de Takayasu<br>2 Arterite de Células Gigantes<br><br>Custom alignment: LV<br>Field Annotation: @NOMISSING

529	antes_gde_grau16<br><br>Show the field ONLY if:<br>[carac_acometim16]='2'	Grau de acometimento	radio<br>1 Localizado<br>2 Extenso<br><br>Custom alignment: LV<br>Field Annotation: @NOMISSING
530	antes_info16<br><br>Show the field ONLY if:<br>[carac_acometim16]='2'	Atividade de doença	descriptive
531	antes_gde_ativ16<br><br>Show the field ONLY if:<br>[carac_acometim16]='2'	Clínico	radio<br>1 Sim<br>0 Não<br>999 Não disponível<br><br>Custom alignment: LV<br>Field Annotation: @NOMISSING
532	antes_gde_lab16<br><br>Show the field ONLY if:<br>[carac_acometim16]='2'	Laboratorial	radio<br>1 Sim<br>0 Não<br>999 Não disponível<br><br>Custom alignment: LV<br>Field Annotation: @NOMISSING
533	antes_lab_vhs16<br><br>Show the field ONLY if:<br>[carac_acometim16]='2'	VHS<br>*Apenas números.*	text (integer)
534	antes_lab_pcr16<br><br>Show the field ONLY if:<br>[carac_acometim16]='2'	PCR<br>*Apenas números.*	text (integer)
535	antes_img16<br><br>Show the field ONLY if:<br>[carac_acometim16]='2'	Imagem?	radio<br>1 Sim<br>0 Não<br><br>Custom alignment: LV<br>Field Annotation: @NOMISSING
536	antes_img_s16<br><br>Show the field ONLY if:<br>[antes_img16]='1'	Qual?	radio<br>1 Angio-CT<br>2 Angio-RM<br>3 US<br><br>Custom alignment: LV<br>Field Annotation: @NOMISSING
537	info_depois_caso16<br><br>Show the field ONLY if:<br>[caso_contr]='1' and [doen_reuma]='16'	Após os sintomas da covid-19 Avaliação do momento da inclusão	descriptive<br>Field Annotation: @NOMISSING
538	info_depois_contr16<br><br>Show the field ONLY if:<br>[caso_contr]='2' and [doen_reuma]='16'	Avaliação atual Avaliação do momento da inclusão	descriptive<br>Field Annotation: @NOMISSING
539	depois_piora_peq_cov16<br><br>Show the field ONLY if:<br>[carac_acometim16]='1'	Houve piora da atividade da doença depois dos sintomas de COVID-19?	radio<br>1 Sim<br>0 Não<br>888 Desconhecido<br><br>Custom alignment: LV<br>Field Annotation: @NOMISSING

540	depois_peq_grau_reuma16<br>Show the field ONLY if:<br>[carac_acometim16]='1'	Qual o grau de atividade da doença?<br>*Indique um valor de 0 a 10 0 = ausência de atividade e 10= atividade intensa.*	text (integer, Min: 0, Max: 10)
541	depois_peq_bvas16<br>Show the field ONLY if:<br>[carac_acometim16]='1'	BVAS (versão 3)<br>*Apenas números. BVAS: Birmingham Vasculitis Activity Score*	text (integer)
542	depois_peq_pan16<br>Show the field ONLY if:<br>[carac_acometim16]='1'	Five-Factor Score (PAN, GEPA e GPA):<br>*PAN: Poliarterite Nodosa GEPA: Granulomatose Eosinofílica com Poliangeíte GPA: Granulomatose com Poliangeíte*	text (integer)
543	depois_peq_ativid16<br>Show the field ONLY if:<br>[carac_acometim16]='1'	Assinale as manifestações clínicas em atividade:	checkbox<br>1 depois_peq_ativid16___1 Articular<br>2 depois_peq_ativid16___2 Cardiovascular<br>3 depois_peq_ativid16___3 Cutânea<br>4 depois_peq_ativid16___4 Gastrointestinal<br>5 depois_peq_ativid16___5 Ocular<br>6 depois_peq_ativid16___6 Pulmonar<br>7 depois_peq_ativid16___7 Renal<br>8 depois_peq_ativid16___8 SN periférico<br>9 depois_peq_ativid16___9 SNC<br>10 depois_peq_ativid16___10 Vias aéreas superiores<br>0 depois_peq_ativid16___0 Nenhuma<br>Custom alignment: LV<br>Field Annotation: @NOMISSING @NONEOFTHEABOVE='0'
544	depois_info_ativ16<br>Show the field ONLY if:<br>[carac_acometim16]='2'	Atividade de doença	descriptive<br>Field Annotation: @NOMISSING
545	depois_gde_ativ16<br>Show the field ONLY if:<br>[carac_acometim16]='2'	Clínico	radio (Matrix)<br>0 Inalterado<br>2 Piorou<br>1 Melhorou<br>Custom alignment: LV<br>Field Annotation: @NOMISSING
546	depois_gde_lab16<br>Show the field ONLY if:<br>[carac_acometim16]='2'	Laboratorial	radio (Matrix)<br>0 Inalterado<br>2 Piorou<br>1 Melhorou<br>Custom alignment: LV<br>Field Annotation: @NOMISSING
547	depois_img16<br>Show the field ONLY if:<br>[carac_acometim16]='2'	Imagem?	radio (Matrix)<br>0 Inalterado<br>2 Piorou<br>1 Melhorou<br>Custom alignment: LV<br>Field Annotation: @NOMISSING
548	depois_img_s16<br>Show the field ONLY if:<br>[depois_img16]='1'	Qual?	radio<br>1 Angio-RM<br>2 Angio-RM<br>3 US<br>Custom alignment: LV<br>Field Annotation: @NOMISSING

549	depois_piora_vasc16<br>Show the field ONLY if:<br>[carac_acometim16]='2'	Houve aparecimento ou piora de outra manifestação de vasculite?	radio<br>1 Sim<br>0 Não<br>Custom alignment: LV<br>Field Annotation: @NOMISSING
550	depois_anca_c16<br>Show the field ONLY if:<br>[carac_acometim16]='2'	ANCA-C (IMF)	radio<br>1 Positivo<br>0 Negativo<br>999 Não disponível<br>Custom alignment: LV<br>Field Annotation: @NOMISSING
551	depois_anca_c_valor16<br>Show the field ONLY if:<br>[depois_anca_c16]='1'	ANCA-C (IMF) valor	text (integer)
552	depois_anca_p16<br>Show the field ONLY if:<br>[carac_acometim16]='2'	ANCA-P (IMF)	radio<br>1 Positivo<br>0 Negativo<br>999 Não disponível<br>Custom alignment: LV<br>Field Annotation: @NOMISSING
553	depois_anca_p_valor16<br>Show the field ONLY if:<br>[depois_anca_p16]='1'	ANCA-P (IMF) valor	text (integer)
554	depois_anca_pr16<br>Show the field ONLY if:<br>[carac_acometim16]='2'	ANCA-PR3	radio<br>1 Positivo<br>0 Negativo<br>999 Não disponível<br>Custom alignment: LV<br>Field Annotation: @NOMISSING
555	depois_anca_pr_valor16<br>Show the field ONLY if:<br>[depois_anca_pr16]='1'	ANCA-PR3 valor	text (integer)
556	depois_anca_mp16<br>Show the field ONLY if:<br>[carac_acometim16]='2'	ANCA-MPO	radio<br>1 Positivo<br>0 Negativo<br>999 Não disponível<br>Custom alignment: LV<br>Field Annotation: @NOMISSING
557	depois_anca_mp_valor16<br>Show the field ONLY if:<br>[depois_anca_mp16]='1'	ANCA-MPO valor	text (integer)
558	depois_lab_vhs16<br>Show the field ONLY if:<br>[carac_acometim16]='2'	VHS	text (integer)
559	depois_lab_pcr16<br>Show the field ONLY if:<br>[carac_acometim16]='2'	PCR	text (integer)
560	depois_piora_drim16<br>Show the field ONLY if:<br>[caso_contr]='1' and ([carac_acometim16]='1' or [carac_acometim16]='2')	Houve aparecimento ou piora de outra manifestação de DRIM?	radio<br>1 Sim<br>0 Não<br>Custom alignment: LV<br>Field Annotation: @NOMISSING

561	depois_piora_drim_s16<br>Show the field ONLY if:<br>[depois_piora_drim16]='1'	Qual?<br>*Aparecimento de outras manifestações imunomediadas não listadas acima ou não diretamente relacionada com a doença de base.*	text<br>Field Annotation: @NOMISSING
562	info_lab_covid<br>Show the field ONLY if:<br>[caso_contr]='1' and [eleg_timo]='0' and [doen_reuma]<>''	EXAMES LABORATORIAIS Realizados nos últimos 30 dias	descriptive<br>Field Annotation: @NOMISSING
563	lab_covid<br>Show the field ONLY if:<br>[caso_contr]='1' and [eleg_timo]='0' and [doen_reuma]<>''	Exames laboratorias estão disponíveis?	radio<br>1 Sim<br>0 Não<br>Custom alignment: LV<br>Field Annotation: @NOMISSING
564	lab_covid_dt<br>Show the field ONLY if:<br>[lab_covid]='1'	Data da coleta do exame laboratorial	text (date_dmy)
565	lab_covid_hb<br>Show the field ONLY if:<br>[lab_covid]='1'	Hb:<br>*Apenas números, separar casas decimais com ponto (.) g/dl*	text (number)
566	lab_covid_hb_dt<br>Show the field ONLY if:<br>[lab_covid]='1'	Data Hb:	text (date_dmy)<br>Field Annotation: @HIDDEN
567	lab_covid_leuc<br>Show the field ONLY if:<br>[lab_covid]='1'	Leucócitos totais:<br>*Apenas números, separar casas decimais com ponto (.) /mm3*	text (number)
568	lab_covid_neut<br>Show the field ONLY if:<br>[lab_covid]='1'	Neutrófilos:<br>*Valor absolutoApenas números, separar casas decimais com ponto (.) /mm3*	text (number)
569	lab_covid_linfoc<br>Show the field ONLY if:<br>[lab_covid]='1'	Linfócitos:<br>*Valor absolutoApenas números, separar casas decimais com ponto (.) /mm3*	text (number)
570	lab_covid_plaq<br>Show the field ONLY if:<br>[lab_covid]='1'	Plaquetas:<br>*Apenas números, separar casas decimais com ponto (.) /mm3*	text (number)
571	lab_covid_protei_valor<br>Show the field ONLY if:<br>[lab_covid]='1'	Proteína C reativa:	radio<br>1 mg/L<br>2 mg/dL
572	lab_covid_protei<br>Show the field ONLY if:<br>[lab_covid_protei_valor]='1' or [lab_covid_protei_valor]='2'	*Apenas números, separar casas decimais com ponto (.)*	text (number)
573	lab_covid_vhs<br>Show the field ONLY if:<br>[lab_covid]='1'	VSH 1ª. Hora<br>*Apenas números, separar casas decimais com ponto (.)*	text (number)
574	lab_covid_alb<br>Show the field ONLY if:<br>[lab_covid]='1'	Albumina:<br>*Apenas números, separar casas decimais com ponto (.) g/dl*	text (number)
575	lab_covid_aldo<br>Show the field ONLY if:<br>[lab_covid]='1'	Aldolase:<br>*Apenas números, separar casas decimais com ponto (.)*	text (number)
576	lab_covid_creat<br>Show the field ONLY if:<br>[lab_covid]='1'	Creatinina:<br>*Apenas números, separar casas decimais com ponto (.) mg/dl*	text (number)
577	lab_covid_dime<br>Show the field ONLY if:<br>[lab_covid]='1'	D-Dímero:<br>*Apenas números, separar casas decimais com ponto (.)*	text (number)

578	lab_covid_desid<br>Show the field ONLY if: [lab_covid]='1'	Desidrogenase lática:<br>*Apenas números, separar casas decimais com ponto (.) mg/dl*	text (number)
579	lab_covid_ferr<br>Show the field ONLY if: [lab_covid]='1'	Ferritina:<br>*Apenas números, separar casas decimais com ponto (.) mg/dl*	text (number)
580	lab_covid_fibri<br>Show the field ONLY if: [lab_covid]='1'	Fibrinogênio:<br>*Apenas números, separar casas decimais com ponto (.)*	text (number)
581	lab_covid_procal<br>Show the field ONLY if: [lab_covid]='1'	Procalcitonina:	text (integer)
582	lab_covid_tgo<br>Show the field ONLY if: [lab_covid]='1'	TGO:<br>*Apenas números, separar casas decimais com ponto (.) mg/dl*	text (number)
583	lab_covid_tgp<br>Show the field ONLY if: [lab_covid]='1'	TGP:<br>*Apenas números, separar casas decimais com ponto (.) mg/dl*	text (number)
584	antes_brbdcaf_s5<br>Show the field ONLY if: [antes_brbdcaf5]='1'	Valor de BR-BDCAFs ou BRBDCAF<br>*Apenas números, separar casas decimais com ponto (.) BR-BDCAFs: Brazilian Behçet's Disease Current Activity Form simplificado BRBDCAF: Brazilian Behçet's Disease Current Activity Form*	text (number)<br>Field Annotation: @NOMISSING
585	lab_covid_trig<br>Show the field ONLY if: [lab_covid]='1'	Triglicerídeos:<br>*Apenas números, separar casas decimais com ponto (.) mg/dl*	text (number)
586	info_visita_seg2<br>Show the field ONLY if: [eleg_timo]='0' and [doen_reuma]<>''	Sobre a visita de seguimento	descriptive
587	seg_v2<br>Show the field ONLY if: [eleg_timo]='0' and [doen_reuma]<>''	Participante de pesquisa irá realizar Visita 2?<br>*Visita será realizada 90 dias após inclusão*	radio<br>1 Sim<br>0 Não<br>Custom alignment: LV
588	seg_v2_nao<br>Show the field ONLY if: [seg_v2]='0'	Porque o participante não irá realizar a visita de seguimento?	radio<br>1 Opção do participante de pesquisa<br>2 Óbito<br>777 Outro motivo<br>Custom alignment: LV
589	seg_v2_nao_obito<br>Show the field ONLY if: [seg_v2_nao]='2' and [atend_hosp_evol]!='2'	Óbito relacionado a COVID-19?	radio<br>1 Sim<br>0 Não<br>Custom alignment: LV
590	seg_v2_nao_obito_dt<br>Show the field ONLY if: [seg_v2_nao_obito]='1'	Data do óbito relacionado a COVID-19?	text (date_dmy)
591	seg_v2_nao_outro<br>Show the field ONLY if: [seg_v2_nao]='777'	Qual outro motivo o participante de pesquisa não irá participar?	text
592	aval_reumatica_complete	Section Header: *Form Status*<br>Complete?	dropdown<br>0 Incomplete<br>1 Unverified<br>2 Complete

Instrument: **Laboratorio** (laboratorio)

593	lab_coleta_sn<br>Show the field ONLY if: [eleg_timo]='0' and [doen_reuma]<>''	Section Header: *AVALIAÇÃO LABORATORIAL CENTRAL DO ESTUDORealização da sorologia para o SARS-CoV-2, para confirmação da suspeita clínica de COVID-19Será coletado 20 ml de sangue em veia periférica, para posterior avaliação. O sangue será centrifugado a 3000 rpm, sendo retirado o soro, que será armazenado em freezer a -80°C nos centros participantes, até que sejam enviados ao Laboratório central*<br>Coleta foi realizada?	radio, Required<br>1 Sim<br>0 Não<br>Custom alignment: LV
594	lab_coleta_dt<br>Show the field ONLY if: [lab_coleta_sn]='1'	Data da coleta	text (date_dmy)
595	lab_cpf<br>Show the field ONLY if: [lab_coleta_sn]='1'	CPF	text
596	lab_nome_mae<br>Show the field ONLY if: [lab_coleta_sn]='1'	Nome da mãe	text
597	lab_endere_o<br>Show the field ONLY if: [lab_coleta_sn]='1'	Endereço<br>Município: [municipio]<br>Estado: [uf]<br>*Rua/Avenida, Nro, Completo, Bairro.*	notes
598	lab_coleta_n<br>Show the field ONLY if: [lab_coleta_sn]='0'	Por qual motivo não realizou a coleta?	notes
599	laboratorio_complete	Section Header: *Form Status*<br>Complete?	dropdown<br>0 Incomplete<br>1 Unverified<br>2 Complete
Instrument: **Seguimento** (seguimento)			
600	seg_info_geral<br>Show the field ONLY if: [inclusao_arm_1][eleg_timo] ='0'	INFORMAÇÕES GERAIS	descriptive<br>Field Annotation: @NOMISSING
601	seg_dt<br>Show the field ONLY if: [inclusao_arm_1][eleg_timo] ='0'	Data da Visita	text (date_dmy)
602	seg_presenca<br>Show the field ONLY if: [inclusao_arm_1][eleg_timo] ='0'	Participante de pesquisa compareceu no período planejado?	radio<br>1 Sim<br>0 Não<br>Custom alignment: LV
603	seg_presenca_n<br>Show the field ONLY if: [seg_presenca]='0'	Motivo do não comparecimento	radio<br>2 Óbito<br>1 Retirou TCLE<br>888 Desconhecido<br>Custom alignment: LV
604	seg_presenca_n_obito<br>Show the field ONLY if: [seg_presenca_n]='1'	Óbito relacionado a COVID-19?	radio<br>1 Sim<br>0 Não<br>2 Em investigação<br>Custom alignment: LV
605	seg_presenca_n_obito_dt<br>Show the field ONLY if: [seg_presenca_n_obito]='1'	Data Óbito relacionado a COVID-19	text (date_dmy)

606	seg_pessoas_isolamento_extra<br>Show the field ONLY if: [seg_presenca]='1'	Todas as pessoas que moram na sua casa, incluindo você, permaneceram em distanciamento social (em casa) durante a epidemia?<br>*Distanciamento social: afastamento de suas atividades profissionais e obrigações familiares e de lazer para reduzir a transmissão, por vontade própria, orientação da empresa onde trabalha ou decreto governamental para serviços não essenciais), saindo apenas para realizar coisas estritamente necessárias, como comprar comida ou ir a farmácia.*	radio<br>1 Sim<br>0 Não<br>Custom alignment: LV<br>Field Annotation: @NOMISSING
607	seg_distancia<br>Show the field ONLY if: [seg_presenca]='1'	No seu município, ainda está sendo recomendado distanciamento social?	radio<br>1 Sim<br>0 Não<br>Custom alignment: LV
608	seg_distancia_dt<br>Show the field ONLY if: [seg_distancia]='0'	Data do término do isolamento social:	text (date_dmy)<br>Field Annotation: @NOMISSING
609	seg_distancia_masc<br>Show the field ONLY if: [seg_presenca]='1'	Durante a epidemia usou máscara ao sair de casa?<br>*Considerar o período que foi solicitado pelas autoridades públicas da sua região*	radio<br>1 Sim<br>0 Não<br>Custom alignment: LV<br>Field Annotation: @NOMISSING
610	seg_vacina<br>Show the field ONLY if: [seg_presenca]='1'	Você tomou vacina de gripe nos últimos 3 meses?	radio<br>1 Sim<br>0 Não<br>Custom alignment: LV<br>Field Annotation: @NOMISSING
611	seg_info_familia<br>Show the field ONLY if: [seg_presenca]='1'	Sobre seu núcleo familiar	descriptive<br>Field Annotation: @NOMISSING
612	seg_pessoas_casa_sim_extra<br>Show the field ONLY if: [seg_presenca]='1'	Alguma outra pessoa do seu núcleo familiar apresentou sintomas de COVID-19?	radio<br>1 Sim<br>0 Não<br>Custom alignment: LV<br>Field Annotation: @NOMISSING
613	seg_familia_covid1<br>Show the field ONLY if: [seg_pessoas_casa_sim_extra] ='1'	No total, quantas pessoas na sua casa (excluindo você) tiveram COVID?<br>*Apenas números.*	text (integer)
614	seg_familia_covid3<br>Show the field ONLY if: [seg_pessoas_casa_sim_extra] ='1'	Quantos foram diagnosticados como suspeito com exame positivo (PCR, teste rápido ou sorologia)<br>*Apenas números.*	text (integer)
615	seg_familia_covid4<br>Show the field ONLY if: [seg_pessoas_casa_sim_extra] ='1'	Quantos foram diagnosticados por Contato com caso de COVID-19 confirmado e sintomas sugestivos nos 14 dias após o contato, sem exame confirmatório.<br>*Apenas números.*	text (integer)
616	seg_info_evol<br>Show the field ONLY if: [seg_presenca]='1' and [inclusao_arm_1][caso_contr]='2'	Informações sobre os sintomas de COVID-19	descriptive<br>Field Annotation: @NOMISSING
617	seg_sintoma<br>Show the field ONLY if: [seg_presenca]='1' and [inclusao_arm_1][caso_contr]='2'	Nos últimos 3 meses, você apresentou sintomas sugestivos de COVID-19?	radio<br>1 Sim<br>0 Não<br>Custom alignment: LV<br>Field Annotation: @NOMISSING

618	seg_sintoma_dt<br><br>Show the field ONLY if:<br>[seg_sintoma]='1'	Data de início dos sintomas:<br>*Padrão Internacional para incerteza de data:"Meio do meio" Exemplo: Insira 15 se existe dúvida quanto ao dia do mês; Insira Junho (15/6/2019) se existe dúvida quanto ao mês do ano.*	text (date_dmy), Required<br>Field Annotation: @NOMISSING
619	seg_sintoma_s<br><br>Show the field ONLY if:<br>[seg_sintoma]='1'	Sintomas apresentados	checkbox, Required<br>15 | seg_sintoma_s___15 | Alterações de pele<br>1 | seg_sintoma_s___1 | Artralgias<br>2 | seg_sintoma_s___2 | Astenia<br>3 | seg_sintoma_s___3 | Cefaleia<br>4 | seg_sintoma_s___4 | Coriza<br>5 | seg_sintoma_s___5 | Diarreia<br>6 | seg_sintoma_s___6 | Dispneia<br>7 | seg_sintoma_s___7 | Febre<br>8 | seg_sintoma_s___8 | Mialgias<br>9 | seg_sintoma_s___9 | Náuseas<br>10 | seg_sintoma_s___10 | Perda de Olfato<br>11 | seg_sintoma_s___11 | Perda de paladar<br>12 | seg_sintoma_s___12 | Tonturas<br>13 | seg_sintoma_s___13 | Tosse<br>14 | seg_sintoma_s___14 | Vômitos<br>777 | seg_sintoma_s___777 | Outros<br><br>Custom alignment: LV<br>Field Annotation: @NOMISSING
620	seg_sintoma_s_pele<br><br>Show the field ONLY if:<br>[seg_sintoma_s(15)]='1'	Qual alteração de pele? Descreva:	notes<br>Field Annotation: @NOMISSING
621	seg_sintoma_s_out<br><br>Show the field ONLY if:<br>[seg_sintoma_s(777)]='1'	Qual outro sintoma não listado? Descreva:	notes<br>Field Annotation: @NOMISSING
622	seg_sintoma_ainda<br><br>Show the field ONLY if:<br>[inclusao_arm_1][caso_contr]<br>='1' and [seg_sintoma]='1'	Ainda apresentando sintomas de COVI-19 ?	radio<br>1 | Sim<br>0 | Não<br><br>Custom alignment: LV<br>Field Annotation: @NOMISSING
623	seg_sintoma_durac<br><br>Show the field ONLY if:<br>[seg_sintoma_ainda]='0'	Tempo de duração dos sintomas<br>*Em dias. Apenas números.*	text (integer)
624	seg_casa<br><br>Show the field ONLY if:<br>[seg_sintoma]='1'	Tratamento em casa?	radio<br>1 | Sim<br>0 | Não<br><br>Custom alignment: LV<br>Field Annotation: @NOMISSING
625	seg_telemedicina<br><br>Show the field ONLY if:<br>[seg_sintoma]='1'	O participante de pesquisa recebeu atendimento por telemedicina?<br>*Atendimento por telemedicina: orientação por profissional de saúde - via contato telefônico ou internet - quanto aos sinais e sintomas e a necessidade, ou não, de procurar o serviço de saúde.*	radio<br>1 | Sim<br>0 | Não<br><br>Custom alignment: LV<br>Field Annotation: @NOMISSING
626	seg_ambulatorio<br><br>Show the field ONLY if:<br>[seg_sintoma]='1'	O participante de pesquisa recebeu atendimento no ambulatório/consultorio?	radio<br>1 | Sim<br>0 | Não<br><br>Custom alignment: LV<br>Field Annotation: @NOMISSING

627	seg_hosp<br>Show the field ONLY if:<br>[seg_sintoma]='1'	O participante de pesquisa necessitou de atendimento hospitalar?	radio<br>1 Sim<br>0 Não<br>Custom alignment: LV<br>Field Annotation: @NOMISSING
628	seg_hosp_tipo<br>Show the field ONLY if:<br>[seg_hosp]='1'	Especifique o local de internação	radio<br>1 Atendido na emergência e liberado para casa<br>2 Internamento enfermaria<br>3 Internamento UTI<br>4 Hospital de campanha<br>5 Observação de até 24h/e/ou gripário (até 24h de observação)<br>Custom alignment: LV<br>Field Annotation: @NOMISSING
629	seg_hosp_dt_ent<br>Show the field ONLY if:<br>[seg_hosp_tipo]='2' or [seg_hosp_tipo]='3' or [seg_hosp_tipo]='4' or [seg_hosp_tipo]='5'	Data da internação	text (date_dmy)<br>Field Annotation: @NOMISSING
630	seg_hosp_ventila<br>Show the field ONLY if:<br>[seg_hosp_tipo]='2' or [seg_hosp_tipo]='3' or [seg_hosp_tipo]='4' or [seg_hosp_tipo]='5'	Precisou de ventilação mecânica(respirador)?	radio<br>1 Sim<br>0 Não<br>Custom alignment: LV<br>Field Annotation: @NOMISSING
631	seg_hosp_evol<br>Show the field ONLY if:<br>[seg_hosp_tipo]='2' or [seg_hosp_tipo]='3' or [seg_hosp_tipo]='4' or [seg_hosp_tipo]='5'	Evolução do internamento?	radio<br>0 Ainda permanece internado<br>1 Alta vivo<br>2 Óbito<br>Custom alignment: LV<br>Field Annotation: @NOMISSING
632	seg_hosp_evol_dt<br>Show the field ONLY if:<br>[seg_hosp_evol]='1' or [seg_hosp_evol]='2'	Indique a data [atend_hosp_evol]	text (date_dmy)<br>Field Annotation: @NOMISSING
633	seg_tomo<br>Show the field ONLY if:<br>[seg_sintoma]='1'	Fez tomografia de tórax? (ao diagnóstico/basal)	radio<br>1 Sim<br>0 Não<br>Custom alignment: LV<br>Field Annotation: @NOMISSING
634	seg_tomo_s<br>Show the field ONLY if:<br>[seg_tomo]='1'	Laudo da tomografia de tórax	radio<br>0 Normal<br>1 Alterada<br>Custom alignment: LV<br>Field Annotation: @NOMISSING
635	seg_tomo_titulo<br>Show the field ONLY if:<br>[seg_tomo_s]='1'	Selecione a alteração da tomografia de tórax	radio<br>1 Vidro fosco < 50%<br>2 Vidro fosco >= 50%<br>3 Condensação<br>4 Piora do padrão habitual<br>Custom alignment: LV<br>Field Annotation: @NOMISSING

636	seg_tomo_dt<br>Show the field ONLY if:<br>[seg_tomo]='1'	Data da tomografia de tórax	text (date_dmy)<br>Field Annotation: @NOMISSING
637	seg_imagem<br>Show the field ONLY if:<br>[seg_tomo]='1'	Fez tomografia de tórax de controle ?	radio<br>1 Sim<br>0 Não<br>Custom alignment: LV<br>Field Annotation: @NOMISSING
638	seg_imagem_titulo<br>Show the field ONLY if:<br>[seg_imagem]='1'	Em relação a tomografia de tórax controle, assinale:	radio<br>0 Inalterada em relação ao basal<br>1 Melhora<br>2 Piora<br>Custom alignment: LV<br>Field Annotation: @NOMISSING
639	seg_imagem_dt<br>Show the field ONLY if:<br>[seg_imagem]='1'	Data da tomografia de tórax de controle	text (date_dmy)<br>Field Annotation: @NOMISSING
640	seg_sintoma_evolui<br>Show the field ONLY if:<br>[seg_sintoma]='1'	Evolui com sequela relacionada a COVID-19?	radio<br>1 Sim<br>0 Não<br>999 Não disponível<br>Custom alignment: LV<br>Field Annotation: @NOMISSING
641	seg_sintoma_evolui_s<br>Show the field ONLY if:<br>[seg_sintoma_evolui]='1'	Qual sequela ?	checkbox<br>1 seg_sintoma_evolui_s___1 Cardiovascular<br>2 seg_sintoma_evolui_s___2 Neurológica<br>3 seg_sintoma_evolui_s___3 Pulmonar<br>4 seg_sintoma_evolui_s___4 Renal<br>777 seg_sintoma_evolui_s___777 Outros<br>Custom alignment: LV<br>Field Annotation: @NOMISSING
642	seg_sintoma_evolui_out<br>Show the field ONLY if:<br>[seg_sintoma_evolui_s(777)]='1'	Qual outra sequela?	text<br>Field Annotation: @NOMISSING
643	seg_info_med<br>Show the field ONLY if:<br>[seg_sintoma]='1'	Nos últimos 3 meses, você fez uso de algum dos medicamentos abaixo? (tratamento da COVID-19)	descriptive<br>Field Annotation: @NOMISSING

644	seg_medicam_covid<br>Show the field ONLY if:<br>[seg_sintoma]='1'	Medicações utilizadas para tratamento da COVID-19	checkbox<br>27 seg_medicam_covid___27 Analgésicos<br>21 seg_medicam_covid___21 Azitromicina<br>9 seg_medicam_covid___9 Corticoide oral<br>32 seg_medicam_covid___32 Heparina<br>28 seg_medicam_covid___28 Hidroxicloroquina/Difosf[illegible] de Cloroquina<br>23 seg_medicam_covid___23 Imunoglobulina EV<br>24 seg_medicam_covid___24 Inibidor de JAK<br>29 seg_medicam_covid___29 Oxigenação de membra[illegible] extra-corpórea (ECMO)<br>31 seg_medicam_covid___31 Plasmaférese<br>25 seg_medicam_covid___25 Pulso com dexametason[illegible]<br>26 seg_medicam_covid___26 Pulso com metilprednisolona<br>30 seg_medicam_covid___30 Soro/Plasma de convalescente<br>18 seg_medicam_covid___18 Tocilizumabe<br>999 seg_medicam_covid___999 Não sabe ou não respondeu<br>0 seg_medicam_covid___0 Nenhuma medicação<br>777 seg_medicam_covid___777 Outros (detalhar abaixo)<br>Custom alignment: LV<br>Field Annotation: @NOMISSING
645	seg_medicam_covid_out<br>Show the field ONLY if:<br>[seg_medicam_covid(777)]='1'	Medicações utilizadas para tratamento da COVID-19	notes<br>Custom alignment: LV<br>Field Annotation: @NOMISSING
646	seg_medicam_covid_cort<br>Show the field ONLY if:<br>[seg_medicam_covid(9)]='1'	Dose corticoide oral	radio<br>1 Até 10 mg/dia<br>2 >=11 a 20 mg/dia<br>3 > 20 mg/dia<br>Custom alignment: LV<br>Field Annotation: @NOMISSING
647	seg_medicam_covid_heparina<br>Show the field ONLY if:<br>[seg_medicam_covid(32)]='1'	Qual dose de heparina?	radio<br>1 Plena<br>2 Profilática<br>Custom alignment: LV<br>Field Annotation: @NOMISSING
648	seg_trat_experim<br>Show the field ONLY if:<br>[seg_hosp]='1'	Durante a internação da infecção pelo novo coronavírus, você recebeu algum tratamento experimental ?	radio<br>1 Sim<br>0 Não<br>999 Não disponível<br>Custom alignment: LV<br>Field Annotation: @NOMISSING
649	seg_trat_experim_s<br>Show the field ONLY if:<br>[seg_trat_experim]='1'	Qual dos abaixo?	radio<br>1 Oxigenação por membrana extra-corpórea<br>2 Plasmaférese<br>3 Soro de convalescente<br>Custom alignment: LV<br>Field Annotation: @NOMISSING

650	seg_diagnostico<br>Show the field ONLY if:<br>[seg_sintoma]='1'	O diagnóstico de COVID-19 foi confirmado por exame laboratorial?<br>**confirmação com o teste do participante de pesquisa em mãos*	radio<br>1 Sim<br>0 Não<br>Custom alignment: LV<br>Field Annotation: @NOMISSING
651	seg_diagnostico_tipo<br>Show the field ONLY if:<br>[seg_diagnostico]='1'	Que tipo de exame foi feito para confirmar o diagnóstico de COVID-19?	radio<br>1 RT-PCR (swab de nariz e garganta)<br>2 Sorologia para SARS-CoV-2<br>3 Teste rápido<br>0 Não sabe ou não lembra<br>Custom alignment: LV<br>Field Annotation: @NOMISSING
652	seg_diagnostico_dt_exame<br>Show the field ONLY if:<br>[seg_diagnostico_tipo]='1' or [seg_diagnostico_tipo]='2' or [seg_diagnostico_tipo]='3'	Qual a data do exame [seg_diagnostico_tipo]	text (date_dmy)<br>Field Annotation: @NOMISSING
653	seg_info_aval<br>Show the field ONLY if:<br>[seg_presenca]='1'	AVALIAÇÃO CLÍNICA GERAL	descriptive
654	seg_peso<br>Show the field ONLY if:<br>[seg_presenca]='1' and [event-name]='v3_arm_1'	Peso<br>*Kg. Apenas números.*	text (number, Max: 1000)
655	seg_altura<br>Show the field ONLY if:<br>[seg_presenca]='1'	Altura<br>*cm. Apenas números.*	text (integer)<br>Field Annotation: @HIDDEN
656	seg_imc<br>Show the field ONLY if:<br>[seg_presenca]='1' and [event-name]='v3_arm_1'	IMC	calc<br>Calculation: [seg_peso]/(([inclusao_arm_1][altura]/100)*([inclusao_arm_1][altura]/100))
657	seg_circ_abdminal<br>Show the field ONLY if:<br>[seg_presenca]='1' and [event-name]='v3_arm_1'	Circ. Abdominal	text (number)
658	seg_pas<br>Show the field ONLY if:<br>[seg_presenca]='1'	PAS	text (integer)
659	seg_pad<br>Show the field ONLY if:<br>[seg_presenca]='1'	PAD	text (integer)
660	seg_asculta<br>Show the field ONLY if:<br>[seg_presenca]='1'	Ausculta respiratória	radio<br>1 Estertores finos<br>2 MV abolido<br>3 Normal<br>4 Roncos<br>5 Sibilos<br>Custom alignment: LV<br>Field Annotation: @NOMISSING
661	seg_outras_altera<br>Show the field ONLY if:<br>[seg_asculta]='1'	Outras alterações exame físico (descreva)<br>*Descreva as alterações consideradas relevantes no exame físico.*	notes<br>Field Annotation: @NOMISSING

662	info_seg1<br>Show the field ONLY if:<br>[inclusao_arm_1][doen_reuma]='1' and [seg_presenca]='1'	ARTRITE ENTEROPÁTICA - [event-label]	descriptive<br>Field Annotation: @NOMISSING
663	seg_piora_caso_cov1<br>Show the field ONLY if:<br>[inclusao_arm_1][caso_contr]='1' and [inclusao_arm_1][doen_reuma]='1' and [seg_presenca]='1'	Houve piora da atividade da doença depois dos sintomas de COVID-19?	radio<br>1 Sim<br>0 Não<br>Custom alignment: LV<br>Field Annotation: @NOMISSING
664	seg_piora_cont_cov1<br>Show the field ONLY if:<br>[inclusao_arm_1][caso_contr]='2' and [inclusao_arm_1][doen_reuma]='1' and [seg_presenca]='1'	Houve piora da atividade da doença nos últimos 3 meses? (caso tenha apresentado sintomas de COVID-19, considere o quadro após os sintomas)	radio<br>1 Sim<br>0 Não<br>Custom alignment: LV<br>Field Annotation: @NOMISSING
665	seg_grau_reuma1<br>Show the field ONLY if:<br>[inclusao_arm_1][doen_reuma]='1' and [seg_presenca]='1'	Qual o grau de atividade da doença no momento?<br>*Indique um valor de 0 a 10 0 = ausência de atividade e 10= atividade intensa.*	text (integer, Min: 0, Max: 10)<br>Field Annotation: @NOMISSING
666	seg_asdas_vsh1<br>Show the field ONLY if:<br>[inclusao_arm_1][doen_reuma]='1' and [seg_presenca]='1'	ASDAS VSH<br>*Apenas números, separar decimais por ponto (.) ASDAS VSH: Ankylosing Spondylitis Disease Activity index calculado com VSH.*	text (number)
667	seg_asdas_pcr1<br>Show the field ONLY if:<br>[inclusao_arm_1][doen_reuma]='1' and [seg_presenca]='1'	ASDAS PCR<br>*Apenas números, separar decimais por ponto (.) ASDAS PCR: Ankylosing Spondylitis Disease Activity index calculado com PCR.*	text (number)
668	seg_info_ativ1<br>Show the field ONLY if:<br>[inclusao_arm_1][doen_reuma]='1' and [seg_presenca]='1'	Em relação às manifestações clínicas iniciais, indique o grau de atividade neste momento:	descriptive<br>Field Annotation: @NOMISSING
669	seg_intestinal1<br>Show the field ONLY if:<br>[inclusao_arm_1][doen_reuma]='1' and [seg_presenca]='1'	Intestinal	radio (Matrix)<br>0 Inalterado<br>1 Piorou<br>2 Melhorou<br>Field Annotation: @NOMISSING
670	seg_articular1<br>Show the field ONLY if:<br>[inclusao_arm_1][doen_reuma]='1' and [seg_presenca]='1'	Articular	radio (Matrix)<br>0 Inalterado<br>1 Piorou<br>2 Melhorou<br>Field Annotation: @NOMISSING
671	seg_entesite1<br>Show the field ONLY if:<br>[inclusao_arm_1][doen_reuma]='1' and [seg_presenca]='1'	Entesite	radio (Matrix)<br>0 Inalterado<br>1 Piorou<br>2 Melhorou<br>Field Annotation: @NOMISSING
672	seg_dactilite1<br>Show the field ONLY if:<br>[inclusao_arm_1][doen_reuma]='1' and [seg_presenca]='1'	Dactilite	radio (Matrix)<br>0 Inalterado<br>1 Piorou<br>2 Melhorou<br>Field Annotation: @NOMISSING

673	seg_uveit1<br>Show the field ONLY if: [inclusao_arm_1][doen_reuma] ='1' and [seg_presenca]='1'	Uveíte	radio (Matrix)<br>0 Inalterado<br>1 Piorou<br>2 Melhorou<br>Field Annotation: @NOMISSING
674	seg_piora_drim1<br>Show the field ONLY if: [inclusao_arm_1][doen_reuma] ='1' and [seg_presenca]='1'	Houve aparecimento ou piora de outra manifestação de DRIM?	radio<br>1 Sim<br>0 Não<br>Custom alignment: LV<br>Field Annotation: @NOMISSING
675	seg_piora_drim_s1<br>Show the field ONLY if: [seg_piora_drim1]='1' and [seg_presenca]='1'	Qual ?<br>*Aparecimento de outras manifestações imunomediadas não listadas acima ou não diretamente relacionada com a doença de base.*	text<br>Field Annotation: @NOMISSING
676	info_seg2<br>Show the field ONLY if: [inclusao_arm_1][doen_reuma] ='2' and [seg_presenca]='1'	ARTRITE IDIOPÁTICA JUVENIL (INÍCIO ANTES DO 16 ANOS) NA IDADE ADULTA - [event-label]	descriptive<br>Field Annotation: @NOMISSING
677	seg_piora_caso_cov2<br>Show the field ONLY if: [inclusao_arm_1][caso_contr] ='1' and [inclusao_arm_1][doen_reuma]='2' and [seg_presenca]='1'	Houve piora da atividade da doença depois dos sintomas de COVID-19?	radio<br>1 Sim<br>0 Não<br>Custom alignment: LV<br>Field Annotation: @NOMISSING
678	seg_piora_cont_cov2<br>Show the field ONLY if: [inclusao_arm_1][caso_contr] ='2' and [inclusao_arm_1][doen_reuma]='2' and [seg_presenca]='1'	Houve piora da atividade da doença nos últimos 3 meses? (caso tenha apresentado sintomas de COVID-19, considere o quadro após os sintomas)	radio<br>1 Sim<br>0 Não<br>Custom alignment: LV<br>Field Annotation: @NOMISSING
679	seg_grau_reuma2<br>Show the field ONLY if: [inclusao_arm_1][doen_reuma] ='2' and [seg_presenca]='1'	Qual o grau de atividade da doença?<br>*Indique um valor de 0 a 10 0 = ausência de atividade e 10= atividade intensa.*	text (integer, Min: 0, Max: 10)
680	seg_info_ativ2<br>Show the field ONLY if: [inclusao_arm_1][doen_reuma] ='2' and [seg_presenca]='1'	Em relação às manifestações clínicas iniciais, indique o grau de atividade neste momento:	descriptive<br>Field Annotation: @NOMISSING
681	seg_intestinal2<br>Show the field ONLY if: [inclusao_arm_1][doen_reuma] ='2' and [seg_presenca]='1'	Intestinal	radio (Matrix)<br>0 Inalterado<br>1 Piorou<br>2 Melhorou<br>Field Annotation: @NOMISSING
682	seg_articular2<br>Show the field ONLY if: [inclusao_arm_1][doen_reuma] ='2' and [seg_presenca]='1'	Articular	radio (Matrix)<br>0 Inalterado<br>1 Piorou<br>2 Melhorou<br>Field Annotation: @NOMISSING
683	seg_entesite2<br>Show the field ONLY if: [inclusao_arm_1][doen_reuma] ='2' and [seg_presenca]='1'	Entesite	radio (Matrix)<br>0 Inalterado<br>1 Piorou<br>2 Melhorou<br>Field Annotation: @NOMISSING

684	seg_dactilite2<br>Show the field ONLY if:<br>[inclusao_arm_1][doen_reuma]='2' and [seg_presenca]='1'	Dactilite	radio (Matrix)<br>0 Inalterado<br>1 Piorou<br>2 Melhorou<br>Field Annotation: @NOMISSING
685	seg_uveit2<br>Show the field ONLY if:<br>[inclusao_arm_1][doen_reuma]='2' and [seg_presenca]='1'	Uveíte	radio (Matrix)<br>0 Inalterado<br>1 Piorou<br>2 Melhorou<br>Field Annotation: @NOMISSING
686	seg_piora_drim2<br>Show the field ONLY if:<br>[inclusao_arm_1][doen_reuma]='2' and [seg_presenca]='1'	Houve aparecimento ou piora de outra manifestação de DRIM?	radio<br>1 Sim<br>0 Não<br>Custom alignment: LV<br>Field Annotation: @NOMISSING
687	seg_piora_drim_s2<br>Show the field ONLY if:<br>[seg_piora_drim2]='1' and [seg_presenca]='1'	Qual?<br>*Aparecimento de outras manifestações imunomediadas não listadas acima ou não diretamente relacionada com a doença de base.*	text<br>Field Annotation: @NOMISSING
688	info_seg3<br>Show the field ONLY if:<br>[inclusao_arm_1][doen_reuma]='3' and [seg_presenca]='1'	ARTRITE PSORIÁSICA (APS) - [event-label]	descriptive<br>Field Annotation: @NOMISSING
689	seg_piora_caso_cov3<br>Show the field ONLY if:<br>[inclusao_arm_1][caso_contr]='1' and [inclusao_arm_1][doen_reuma]='3' and [seg_presenca]='1'	Houve piora da atividade da doença depois dos sintomas de COVID-19?	radio<br>1 Sim<br>0 Não<br>Custom alignment: LV<br>Field Annotation: @NOMISSING
690	seg_piora_cont_cov3<br>Show the field ONLY if:<br>[inclusao_arm_1][caso_contr]='2' and [inclusao_arm_1][doen_reuma]='3' and [seg_presenca]='1'	Houve piora da atividade da doença nos últimos 3 meses? (caso tenha apresentado sintomas de COVID-19, considere o quadro após os sintomas)	radio<br>1 Sim<br>0 Não<br>Custom alignment: LV<br>Field Annotation: @NOMISSING
691	seg_grau_reuma3<br>Show the field ONLY if:<br>[inclusao_arm_1][doen_reuma]='3' and [seg_presenca]='1'	Qual o grau de atividade da doença?<br>*Indique um valor de 0 a 10 0 = ausência de atividade e 10= atividade intensa.*	text (integer, Min: 0, Max: 10)<br>Field Annotation: @NOMISSING
692	seg_ativos3<br>Show the field ONLY if:<br>[inclusao_arm_1][doen_reuma]='3' and [seg_presenca]='1'	Assinale as manifestações clínicas que estavam em atividade	checkbox<br>1 seg_ativos3___1 Artrite ativa<br>2 seg_ativos3___2 Dactilite<br>3 seg_ativos3___3 Doença Axial<br>4 seg_ativos3___4 Entesite<br>5 seg_ativos3___5 Pele ou unha<br>Custom alignment: LV<br>Field Annotation: @NOMISSING
693	seg_parametros3<br>Show the field ONLY if:<br>[inclusao_arm_1][doen_reuma]='3' and [seg_presenca]='1'	Parâmetros de atividade estão disponíveis?	radio<br>1 Sim<br>0 Não<br>Custom alignment: LV<br>Field Annotation: @NOMISSING

694	seg_dapsa3<br>Show the field ONLY if:<br>[seg_parametros3]='1'	DAPSA<br>*Apenas números. DAPSA: Disease Activity in PSoriatic Arthritis.*	text (integer)
695	seg_pasi3<br>Show the field ONLY if:<br>[seg_parametros3]='1'	PASI<br>*Apenas números. PASI: Psoriaisis Area Severity Index.*	text (integer)
696	seg_mda3<br>Show the field ONLY if:<br>[inclusao_arm_1][doen_reuma]='3' and [seg_presenca]='1'	Estava MDA (Mínima Atividade de Doença)<br>*5 dos 7 itens abaixo: -Articulações dolorosas ≤ 1 -Articulações edemaciadas ≤ 1 -Pontos de entesite ≤ 1 -PASI ≤1ou superfície de área corpórea ≤ 3 -Escala visual analógica de dor do paciente ≤ 15 - avaliação global da doença pelo paciente ≤ 20 - HAQ ≤ 0,5*	radio<br>1 Sim<br>0 Não<br>Custom alignment: LV<br>Field Annotation: @NOMISSING
697	seg_piora_atv3<br>Show the field ONLY if:<br>[inclusao_arm_1][doen_reuma]='3' and [seg_presenca]='1'	Houve piora da atividade da doença?	radio<br>1 Sim<br>0 Não<br>Custom alignment: LV<br>Field Annotation: @NOMISSING
698	seg_piora_atv_s3<br>Show the field ONLY if:<br>[inclusao_arm_1][doen_reuma]='3' and [seg_presenca]='1'	Qual desfecho?	radio<br>1 Artrite ativa<br>2 Dactilite<br>3 Doença Axial<br>4 Entesite<br>5 Pele ou unha<br>Custom alignment: LV<br>Field Annotation: @NOMISSING
699	seg_piora_drim3<br>Show the field ONLY if:<br>[inclusao_arm_1][doen_reuma]='3' and [seg_presenca]='1'	Houve aparecimento ou piora de outra manifestação de DRIM?	radio<br>1 Sim<br>0 Não<br>Custom alignment: LV<br>Field Annotation: @NOMISSING
700	seg_piora_drim_s3<br>Show the field ONLY if:<br>[seg_piora_drim3]='1'	Qual?<br>*Aparecimento de outras manifestações imunomediadas não listadas acima ou não diretamente relacionada com a doença de base.*	text<br>Field Annotation: @NOMISSING
701	info_seg4<br>Show the field ONLY if:<br>[inclusao_arm_1][doen_reuma]='4' and [seg_presenca]='1'	ARTRITE REUMATOIDE - [event-label]	descriptive<br>Field Annotation: @NOMISSING
702	seg_piora_caso_cov4<br>Show the field ONLY if:<br>[inclusao_arm_1][caso_contr]='1' and [seg_presenca]='1' and ([inclusao_arm_1][doen_reuma]='4' or [seg_predomina14]='4')	Houve piora da atividade da doença depois dos sintomas de COVID-19?	radio<br>1 Sim<br>0 Não<br>Custom alignment: LV<br>Field Annotation: @NOMISSING
703	seg_piora_cont_cov4<br>Show the field ONLY if:<br>[inclusao_arm_1][caso_contr]='2' and [seg_presenca]='1' and ([inclusao_arm_1][doen_reuma]='4' or [seg_predomina14]='4')	Houve piora da atividade da doença nos últimos 3 meses? (caso tenha apresentado sintomas de COVID-19, considere o quadro após os sintomas)	radio<br>1 Sim<br>0 Não<br>Custom alignment: LV<br>Field Annotation: @NOMISSING
704	seg_grau_reuma4<br>Show the field ONLY if:<br>([inclusao_arm_1][doen_reuma]='4' or [seg_predomina14]='4') and [seg_presenca]='1'	Qual o grau de atividade da doença?<br>*Indique um valor de 0 a 10 0 = ausência de atividade e 10= atividade intensa.*	text (integer, Min: 0, Max: 10)

705	seg_cdai_4<br>Show the field ONLY if: ([inclusao_arm_1][doen_reuma]='4' or [seg_predomina14]='4') and [seg_presenca]='1'	Qual o CDAI atual?<br>*Apenas números. CDAI: Clinical Disease Activity Index Valor entre 0 e 76.*	text (integer, Min: 0, Max: 76)
706	seg_art_dolo4<br>Show the field ONLY if: ([inclusao_arm_1][doen_reuma]='4' or [seg_predomina14]='4') and [seg_presenca]='1'	Número articulações dolorosas<br>*Apenas números. Valor entre 0 e 28.*	text (integer, Min: 0, Max: 28)
707	seg_art_ede4<br>Show the field ONLY if: ([inclusao_arm_1][doen_reuma]='4' or [seg_predomina14]='4') and [seg_presenca]='1'	Número articulações edemaciadas<br>*Apenas números. Valor entre 0 e 28.*	text (integer, Min: 0, Max: 28)
708	seg_aval_doe_pac4<br>Show the field ONLY if: ([inclusao_arm_1][doen_reuma]='4' or [seg_predomina14]='4') and [seg_presenca]='1'	Avaliação de atividade de doença pelo paciente<br>*Apenas números. Valor entre 0 e 10*	text (integer, Min: 0, Max: 10)
709	seg_aval_doe_aval4<br>Show the field ONLY if: ([inclusao_arm_1][doen_reuma]='4' or [seg_predomina14]='4') and [seg_presenca]='1'	Avaliação de atividade de doença pelo avaliador<br>*Apenas números. Valor entre 0 e 10*	text (integer, Min: 0, Max: 10)
710	seg_piora_drim4<br>Show the field ONLY if: ([inclusao_arm_1][doen_reuma]='4' or [seg_predomina14]='4') and [seg_presenca]='1'	Houve aparecimento ou piora de outra manifestação de DRIM?	radio<br>1 Sim<br>0 Não<br>Custom alignment: LV<br>Field Annotation: @NOMISSING
711	seg_piora_drim_s4<br>Show the field ONLY if: [seg_piora_drim4]='1'	Qual?<br>*Aparecimento de outras manifestações imunomediadas não listadas acima ou não diretamente relacionada com a doença de base.*	text<br>Field Annotation: @NOMISSING
712	info_seg5<br>Show the field ONLY if: [inclusao_arm_1][doen_reuma]='5' and [seg_presenca]='1'	DOENÇA DE BEHÇET - [event-label]	descriptive<br>Field Annotation: @NOMISSING
713	seg_piora_caso_cov5<br>Show the field ONLY if: [inclusao_arm_1][caso_contr]='1' and [inclusao_arm_1][doen_reuma]='5' and [seg_presenca]='1'	Houve piora da atividade da doença depois dos sintomas de COVID-19?	radio<br>1 Sim<br>0 Não<br>Custom alignment: LV<br>Field Annotation: @NOMISSING
714	seg_piora_cont_cov5<br>Show the field ONLY if: [inclusao_arm_1][caso_contr]='2' and [inclusao_arm_1][doen_reuma]='5' and [seg_presenca]='1'	Houve piora da atividade da doença nos últimos 3 meses? (caso tenha apresentado sintomas de COVID-19, considere o quadro após os sintomas)	radio<br>1 Sim<br>0 Não<br>Custom alignment: LV<br>Field Annotation: @NOMISSING
715	seg_grau_reuma5<br>Show the field ONLY if: [inclusao_arm_1][doen_reuma]='5' and [seg_presenca]='1'	Qual o grau de atividade da doença?<br>*Indique um valor de 0 a 10 0 = ausência de atividade e 10= atividade intensa.*	text (integer, Min: 0, Max: 10)
716	seg_info_ativ5<br>Show the field ONLY if: [inclusao_arm_1][doen_reuma]='5' and [seg_presenca]='1'	Em relação às manifestações clínicas iniciais, indique o grau de atividade neste momento:	descriptive

717	seg_lesoes_pele5<br>Show the field ONLY if: [inclusao_arm_1][doen_reuma] ='5' and [seg_presenca]='1'	Lesões de pele (eritema nodoso/foliculite	radio (Matrix)<br>0 Inalterado<br>2 Piorou<br>1 Melhorou<br>Field Annotation: @NOMISSING
718	seg_manifest5<br>Show the field ONLY if: [inclusao_arm_1][doen_reuma] ='5' and [seg_presenca]='1'	Manifestações neurológicas	radio (Matrix)<br>0 Inalterado<br>2 Piorou<br>1 Melhorou<br>Field Annotation: @NOMISSING
719	seg_ocular5<br>Show the field ONLY if: [inclusao_arm_1][doen_reuma] ='5' and [seg_presenca]='1'	Ocular	radio (Matrix)<br>0 Inalterado<br>2 Piorou<br>1 Melhorou<br>Field Annotation: @NOMISSING
720	seg_nerv_central5<br>Show the field ONLY if: [inclusao_arm_1][doen_reuma] ='5' and [seg_presenca]='1'	Sistema nervoso central	radio (Matrix)<br>0 Inalterado<br>2 Piorou<br>1 Melhorou<br>Field Annotation: @NOMISSING
721	seg_nerv_perif5<br>Show the field ONLY if: [inclusao_arm_1][doen_reuma] ='5' and [seg_presenca]='1'	Sistema nervoso periférico	radio (Matrix)<br>0 Inalterado<br>2 Piorou<br>1 Melhorou<br>Field Annotation: @NOMISSING
722	seg_ulc_genit5<br>Show the field ONLY if: [inclusao_arm_1][doen_reuma] ='5' and [seg_presenca]='1'	Úlceras genitais	radio (Matrix)<br>0 Inalterado<br>2 Piorou<br>1 Melhorou<br>Field Annotation: @NOMISSING
723	seg_ulc_oral5<br>Show the field ONLY if: [inclusao_arm_1][doen_reuma] ='5' and [seg_presenca]='1'	Úlceras orais	radio (Matrix)<br>0 Inalterado<br>2 Piorou<br>1 Melhorou<br>Field Annotation: @NOMISSING
724	seg_novos_fenom5<br>Show the field ONLY if: [inclusao_arm_1][doen_reuma] ='5' and [seg_presenca]='1'	Novos fenômenos tromboembólicos	radio<br>1 Sim<br>0 Não<br>Custom alignment: LV<br>Field Annotation: @NOMISSING

725	seg_sitio5<br>Show the field ONLY if:<br>[seg_novos_fenom5]='1'	Qual sítio do novo fenômeno tromboembólicos?	radio<br>1 AVC<br>2 Oclusão Arterial Aguda<br>3 Retina<br>4 Tromboembolismo Pulmonar<br>5 Tromboflebite superficial<br>6 Trombose Venosa Profunda<br>777 Outros<br>Custom alignment: LV<br>Field Annotation: @NOMISSING
726	seg_sitio_out5<br>Show the field ONLY if:<br>[seg_sitio5]='777'	Qual OUTRO sítio do novo fenômeno tromboembólicos?	text<br>Field Annotation: @NOMISSING
727	seg_brbdcaf5<br>Show the field ONLY if:<br>[inclusao_arm_1][doen_reuma]='5' and [seg_presenca]='1'	BR-BDCAFs ou BRBDCAF disponível?<br>*BR-BDCAFs: Brazilian Behçet's Disease Current Activity Form simplificado BRBDCAF: Brazilian Behçet's Disease Current Activity Form*	radio<br>1 Sim<br>0 Não<br>Custom alignment: LV<br>Field Annotation: @NOMISSING
728	seg_brbdcaf_s5<br>Show the field ONLY if:<br>[seg_brbdcaf5]='1'	Valor de BR-BDCAFs ou BRBDCAF<br>*Apenas números. BR-BDCAFs: Brazilian Behçet's Disease Current Activity Form simplificado BRBDCAF: Brazilian Behçet's Disease Current Activity Form*	text (integer)
729	seg_piora_drim5<br>Show the field ONLY if:<br>[inclusao_arm_1][doen_reuma]='5' and [seg_presenca]='1'	Houve aparecimento ou piora de outra manifestação de DRIM?	radio<br>1 Sim<br>0 Não<br>Custom alignment: LV<br>Field Annotation: @NOMISSING
730	seg_piora_drim_s5<br>Show the field ONLY if:<br>[seg_piora_drim5]='1'	Qual?<br>*Aparecimento de outras manifestações imunomediadas não listadas acima ou não diretamente relacionada com a doença de base.*	text<br>Field Annotation: @NOMISSING
731	info_seg6<br>Show the field ONLY if:<br>[inclusao_arm_1][doen_reuma]='6' and [seg_presenca]='1'	DOENÇA DE STILL DO ADULTO - [event-label]	descriptive<br>Field Annotation: @NOMISSING
732	seg_piora_caso_cov6<br>Show the field ONLY if:<br>[inclusao_arm_1][caso_contr]='1' and [inclusao_arm_1][doen_reuma]='6' and [seg_presenca]='1'	Houve piora da atividade da doença depois dos sintomas de COVID-19?	radio<br>1 Sim<br>0 Não<br>Custom alignment: LV<br>Field Annotation: @NOMISSING
733	seg_piora_cont_cov6<br>Show the field ONLY if:<br>[inclusao_arm_1][caso_contr]='2' and [inclusao_arm_1][doen_reuma]='6' and [seg_presenca]='1'	Houve piora da atividade da doença nos últimos 3 meses? (caso tenha apresentado sintomas de COVID-19, considere o quadro após os sintomas)	radio<br>1 Sim<br>0 Não<br>Custom alignment: LV<br>Field Annotation: @NOMISSING
734	seg_grau_reuma6<br>Show the field ONLY if:<br>[inclusao_arm_1][doen_reuma]='6' and [seg_presenca]='1'	Qual o grau de atividade da doença?<br>*Indique um valor de 0 a 10 0 = ausência de atividade e 10= atividade intensa.*	text (integer, Min: 0, Max: 10)
735	seg_info_ativ6<br>Show the field ONLY if:<br>[inclusao_arm_1][doen_reuma]='6' and [seg_presenca]='1'	Em relação às manifestações clínicas iniciais, indique o grau de atividade neste momento:	descriptive<br>Field Annotation: @NOMISSING

736	seg_adenome6<br>Show the field ONLY if: [inclusao_arm_1][doen_reuma] ='6' and [seg_presenca]='1'	Adenomegalia	radio (Matrix)<br>0 Inalterado<br>2 Piorou<br>1 Melhorou<br>Field Annotation: @NOMISSING
737	seg_articular6<br>Show the field ONLY if: [inclusao_arm_1][doen_reuma] ='6' and [seg_presenca]='1'	Articular	radio (Matrix)<br>0 Inalterado<br>2 Piorou<br>1 Melhorou<br>Field Annotation: @NOMISSING
738	seg_espeno6<br>Show the field ONLY if: [inclusao_arm_1][doen_reuma] ='6' and [seg_presenca]='1'	Espenomegalia	radio (Matrix)<br>0 Inalterado<br>2 Piorou<br>1 Melhorou<br>Field Annotation: @NOMISSING
739	seg_febre6<br>Show the field ONLY if: [inclusao_arm_1][doen_reuma] ='6' and [seg_presenca]='1'	Febre	radio (Matrix)<br>0 Inalterado<br>2 Piorou<br>1 Melhorou<br>Field Annotation: @NOMISSING
740	seg_laborat6<br>Show the field ONLY if: [inclusao_arm_1][doen_reuma] ='6' and [seg_presenca]='1'	Laboratorial	radio (Matrix)<br>0 Inalterado<br>2 Piorou<br>1 Melhorou<br>Field Annotation: @NOMISSING
741	seg_rash6<br>Show the field ONLY if: [inclusao_arm_1][doen_reuma] ='6' and [seg_presenca]='1'	Rash	radio (Matrix)<br>0 Inalterado<br>2 Piorou<br>1 Melhorou<br>Field Annotation: @NOMISSING
742	seg_serosit6<br>Show the field ONLY if: [inclusao_arm_1][doen_reuma] ='6' and [seg_presenca]='1'	Serosites	radio (Matrix)<br>0 Inalterado<br>2 Piorou<br>1 Melhorou<br>Field Annotation: @NOMISSING
743	seg_piora_drim6<br>Show the field ONLY if: [inclusao_arm_1][doen_reuma] ='6' and [seg_presenca]='1'	Houve aparecimento ou piora de outra manifestação de DRIM?	radio<br>1 Sim<br>0 Não<br>Field Annotation: @NOMISSING
744	seg_piora_drim_s6<br>Show the field ONLY if: [seg_piora_drim6]='1'	Qual?<br>*Aparecimento de outras manifestações imunomediadas não listadas acima ou não diretamente relacionada com a doença de base.*	text<br>Field Annotation: @NOMISSING
745	info_seg7<br>Show the field ONLY if: [inclusao_arm_1][doen_reuma] ='7' and [seg_presenca]='1'	DOENÇA MISTA DO TECIDO CONJUNTIVO - [event-label]	descriptive<br>Field Annotation: @NOMISSING

746	seg_piora_caso_cov7<br>Show the field ONLY if:<br>[inclusao_arm_1][caso_contr]='1' and [inclusao_arm_1][doen_reuma]='7' and [seg_presenca]='1'	Houve piora da atividade da doença depois dos sintomas de COVID-19?	radio<br>1 Sim<br>0 Não<br>Custom alignment: LV<br>Field Annotation: @NOMISSING
747	seg_piora_cont_cov7<br>Show the field ONLY if:<br>[inclusao_arm_1][caso_contr]='2' and [inclusao_arm_1][doen_reuma]='7' and [seg_presenca]='1'	Houve piora da atividade da doença nos últimos 3 meses? (caso tenha apresentado sintomas de COVID-19, considere o quadro após os sintomas)	radio<br>1 Sim<br>0 Não<br>Custom alignment: LV<br>Field Annotation: @NOMISSING
748	seg_grau_reuma7<br>Show the field ONLY if:<br>[inclusao_arm_1][doen_reuma]='7' and [seg_presenca]='1'	Qual o grau de atividade da doença?<br>*Indique um valor de 0 a 10 0 = ausência de atividade e 10= atividade intensa.*	text (integer, Min: 0, Max: 10)
749	seg_info_ativ7<br>Show the field ONLY if:<br>[inclusao_arm_1][doen_reuma]='7' and [seg_presenca]='1'	Em relação às manifestações clínicas iniciais, indique o grau de atividade neste momento:	descriptive<br>Field Annotation: @NOMISSING
750	seg_artrite7<br>Show the field ONLY if:<br>[inclusao_arm_1][doen_reuma]='7' and [seg_presenca]='1'	Artrite	radio (Matrix)<br>0 Inalterado<br>2 Piorou<br>1 Melhorou<br>Field Annotation: @NOMISSING
751	seg_dismotilid7<br>Show the field ONLY if:<br>[inclusao_arm_1][doen_reuma]='7' and [seg_presenca]='1'	Dismotilidade esofágica	radio (Matrix)<br>0 Inalterado<br>2 Piorou<br>1 Melhorou<br>Field Annotation: @NOMISSING
752	seg_intersticial7<br>Show the field ONLY if:<br>[inclusao_arm_1][doen_reuma]='7' and [seg_presenca]='1'	Doença intersticial pulmonar	radio (Matrix)<br>0 Inalterado<br>2 Piorou<br>1 Melhorou<br>Field Annotation: @NOMISSING
753	seg_espessam7<br>Show the field ONLY if:<br>[inclusao_arm_1][doen_reuma]='7' and [seg_presenca]='1'	Espessamento de pele	radio (Matrix)<br>0 Inalterado<br>2 Piorou<br>1 Melhorou<br>Field Annotation: @NOMISSING
754	seg_raynaud7<br>Show the field ONLY if:<br>[inclusao_arm_1][doen_reuma]='7' and [seg_presenca]='1'	Fenômeno de Raynaud	radio (Matrix)<br>0 Inalterado<br>2 Piorou<br>1 Melhorou<br>Field Annotation: @NOMISSING
755	seg_glomeru7<br>Show the field ONLY if:<br>[inclusao_arm_1][doen_reuma]='7' and [seg_presenca]='1'	Glomerulonefrite	radio (Matrix)<br>0 Inalterado<br>2 Piorou<br>1 Melhorou<br>Field Annotation: @NOMISSING

756	seg_hipert7<br>Show the field ONLY if: [inclusao_arm_1][doen_reuma] ='7' and [seg_presenca]='1'	Hipertensão arterial pulmonar	radio (Matrix)<br>0 Inalterado<br>2 Piorou<br>1 Melhorou<br>Field Annotation: @NOMISSING
757	seg_pele7<br>Show the field ONLY if: [inclusao_arm_1][doen_reuma] ='7' and [seg_presenca]='1'	Lesões de pele (rash malar ou outras lesões inflamatórias)	radio (Matrix)<br>0 Inalterado<br>2 Piorou<br>1 Melhorou<br>Field Annotation: @NOMISSING
758	seg_neuropat7<br>Show the field ONLY if: [inclusao_arm_1][doen_reuma] ='7' and [seg_presenca]='1'	Neuropatia periférica	radio (Matrix)<br>0 Inalterado<br>2 Piorou<br>1 Melhorou<br>Field Annotation: @NOMISSING
759	seg_vasculite7<br>Show the field ONLY if: [inclusao_arm_1][doen_reuma] ='7' and [seg_presenca]='1'	Vasculite cutânea	radio (Matrix)<br>0 Inalterado<br>2 Piorou<br>1 Melhorou<br>Field Annotation: @NOMISSING
760	seg_piora_drim7<br>Show the field ONLY if: [inclusao_arm_1][doen_reuma] ='7' and [seg_presenca]='1'	Houve aparecimento ou piora de outra manifestação de DRIM?	radio<br>1 Sim<br>0 Não<br>Custom alignment: LV<br>Field Annotation: @NOMISSING
761	seg_piora_drim_s7<br>Show the field ONLY if: [seg_piora_drim7]='1'	Qual?<br>*Aparecimento de outras manifestações imunomediadas não listadas acima ou não diretamente relacionada com a doença de base.*	text<br>Field Annotation: @NOMISSING
762	info_seg8<br>Show the field ONLY if: [inclusao_arm_1][doen_reuma] ='8' and [seg_presenca]='1'	ESCLEROSE SISTÊMICA - [event-label]	descriptive<br>Field Annotation: @NOMISSING
763	seg_piora_caso_cov8<br>Show the field ONLY if: [inclusao_arm_1][caso_contr] ='1' and [seg_presenca]='1' and ([inclusao_arm_1][doen_reum a]='8' or [seg_predomina14] ='8')	Houve piora da atividade da doença depois dos sintomas de COVID-19?	radio<br>1 Sim<br>0 Não<br>Custom alignment: LV<br>Field Annotation: @NOMISSING
764	seg_piora_cont_cov8<br>Show the field ONLY if: [inclusao_arm_1][caso_contr] ='2' and [seg_presenca]='1' and ([inclusao_arm_1][doen_reum a]='8' or [seg_predomina14] ='8')	Houve piora da atividade da doença nos últimos 3 meses? (caso tenha apresentado sintomas de COVID-19, considere o quadro após os sintomas)	radio<br>1 Sim<br>0 Não<br>Custom alignment: LV<br>Field Annotation: @NOMISSING
765	seg_grau_reuma8<br>Show the field ONLY if: ([inclusao_arm_1][doen_reum a]='8' or [seg_predomina14] ='8') and [seg_presenca]='1'	Qual o grau de atividade da doença?<br>*Indique um valor de 0 a 10 0 = ausência de atividade e 10= atividade intensa.*	text (integer, Min: 0, Max: 10)
766	seg_info_ativ8<br>Show the field ONLY if: [inclusao_arm_1][doen_reuma] ='8' and [seg_presenca]='1'	Em relação às manifestações clínicas iniciais, indique o grau de atividade neste momento:	descriptive<br>Field Annotation: @NOMISSING

767	seg_intersticial8<br>Show the field ONLY if: ([inclusao_arm_1][doen_reuma]='8' or [seg_predomina14]='8') and [seg_presenca]='1'	Doença intersticial pulmonar	radio (Matrix)<br>0 Inalterado<br>2 Piorou<br>1 Melhorou<br>Field Annotation: @NOMISSING
768	seg_espessam8<br>Show the field ONLY if: ([inclusao_arm_1][doen_reuma]='8' or [seg_predomina14]='8') and [seg_presenca]='1'	Espessamento da pele	radio (Matrix)<br>0 Inalterado<br>2 Piorou<br>1 Melhorou<br>Field Annotation: @NOMISSING
769	seg_raynaud8<br>Show the field ONLY if: ([inclusao_arm_1][doen_reuma]='8' or [seg_predomina14]='8') and [seg_presenca]='1'	Fenômeno de Raynaud	radio (Matrix)<br>0 Inalterado<br>2 Piorou<br>1 Melhorou<br>Field Annotation: @NOMISSING
770	seg_gastro8<br>Show the field ONLY if: ([inclusao_arm_1][doen_reuma]='8' or [seg_predomina14]='8') and [seg_presenca]='1'	Gastrointestinal	radio (Matrix)<br>0 Inalterado<br>2 Piorou<br>1 Melhorou<br>Field Annotation: @NOMISSING
771	seg_hipert8<br>Show the field ONLY if: ([inclusao_arm_1][doen_reuma]='8' or [seg_predomina14]='8') and [seg_presenca]='1'	Hipertensão arterial pumonar	radio (Matrix)<br>0 Inalterado<br>2 Piorou<br>1 Melhorou<br>Field Annotation: @NOMISSING
772	seg_renal8<br>Show the field ONLY if: ([inclusao_arm_1][doen_reuma]='8' or [seg_predomina14]='8') and [seg_presenca]='1'	Renal (crise renal esclerodérmica)	radio (Matrix)<br>0 Inalterado<br>2 Piorou<br>1 Melhorou<br>Field Annotation: @NOMISSING
773	seg_ulcera8<br>Show the field ONLY if: ([inclusao_arm_1][doen_reuma]='8' or [seg_predomina14]='8') and [seg_presenca]='1'	Úlceras digitais	radio (Matrix)<br>0 Inalterado<br>2 Piorou<br>1 Melhorou<br>Field Annotation: @NOMISSING
774	seg_rodnan8<br>Show the field ONLY if: ([inclusao_arm_1][doen_reuma]='8' or [seg_predomina14]='8') and [seg_presenca]='1'	Escore de Rodnan disponível?	radio<br>1 Sim<br>0 Não<br>Custom alignment: LV<br>Field Annotation: @NOMISSING
775	seg_rodnan_s8<br>Show the field ONLY if: [seg_rodnan8]='1'	Valor Escore de Rodnan<br>*Apenas números. CVF: Capacidade Vital Forçada*	text (integer)
776	seg_distancia8<br>Show the field ONLY if: ([inclusao_arm_1][doen_reuma]='8' or [seg_predomina14]='8') and [seg_presenca]='1'	Teste de caminhada em 6 minutos mais recente disponível em prontuário (dos últimos 6 meses):Distância percorrida	text

777	seg_pulmonar8<br>Show the field ONLY if: ([inclusao_arm_1][doen_reuma]='8' or [seg_predomina14]='8') and [seg_presenca]='1'	Prova de função pulmonar (comparando em relação ao padrão habitual da doença de base):	descriptive
778	seg_cvf8<br>Show the field ONLY if: ([inclusao_arm_1][doen_reuma]='8' or [seg_predomina14]='8') and [seg_presenca]='1'	CVF<br>*Apenas números. CVF: Capacidade Vital Forçada*	text (integer)
779	seg_dlco8<br>Show the field ONLY if: ([inclusao_arm_1][doen_reuma]='8' or [seg_predomina14]='8') and [seg_presenca]='1'	DLCO<br>*Apenas números. DLCO: Capacidade de Difusão do Monóxido de Carbono*	text (integer)
780	seg_tomograf8<br>Show the field ONLY if: ([inclusao_arm_1][doen_reuma]='8' or [seg_predomina14]='8') and [seg_presenca]='1'	Tomografia computadorizada de tórax disponível? (comparando em relação ao padrão habitual da doença de base)	radio<br>1 Sim<br>0 Não<br>Custom alignment: LV<br>Field Annotation: @NOMISSING
781	seg_tomograf_s8<br>Show the field ONLY if: [seg_tomograf8]='1'	Tomografia computadorizada de tórax	radio<br>0 Inalterado<br>2 Piorou<br>1 Melhorou<br>Custom alignment: LV<br>Field Annotation: @NOMISSING
782	seg_piora_drim8<br>Show the field ONLY if: ([inclusao_arm_1][doen_reuma]='8' or [seg_predomina14]='8') and [seg_presenca]='1'	Houve aparecimento ou piora de outra manifestação de DRIM?	radio<br>1 Sim<br>0 Não<br>Field Annotation: @NOMISSING
783	seg_piora_drim_s8<br>Show the field ONLY if: [seg_piora_drim8]='1'	Qual?<br>*Aparecimento de outras manifestações imunomediadas não listadas acima ou não diretamente relacionada com a doença de base.*	text<br>Field Annotation: @NOMISSING
784	info_seg9<br>Show the field ONLY if: [inclusao_arm_1][doen_reuma]='9' and [seg_presenca]='1'	ESPONDILOARTRITE AXIAL (espondilite anquilosante e não radiográfica): EpA axial - [event-label]	descriptive<br>Field Annotation: @NOMISSING
785	seg_piora_caso_cov9<br>Show the field ONLY if: [inclusao_arm_1][caso_contr]='1' and [inclusao_arm_1][doen_reuma]='9' and [seg_presenca]='1'	Houve piora da atividade da doença depois dos sintomas de COVID-19?	radio<br>1 Sim<br>0 Não<br>Custom alignment: LV<br>Field Annotation: @NOMISSING
786	seg_piora_cont_cov9<br>Show the field ONLY if: [inclusao_arm_1][caso_contr]='2' and [inclusao_arm_1][doen_reuma]='9' and [seg_presenca]='1'	Houve piora da atividade da doença nos últimos 3 meses? (caso tenha apresentado sintomas de COVID-19, considere o quadro após os sintomas)	radio<br>1 Sim<br>0 Não<br>Custom alignment: LV<br>Field Annotation: @NOMISSING
787	seg_piora_s_cov19<br>Show the field ONLY if: [seg_piora_caso_cov9]='1' or [seg_piora_cont_cov9]='1'	Qual ?	checkbox<br>1 seg_piora_s_cov19___1 Articulares axiais<br>2 seg_piora_s_cov19___2 Extra-articulares<br>3 seg_piora_s_cov19___3 Periféricas<br>Custom alignment: LV<br>Field Annotation: @NOMISSING

788	seg_grau_reuma9<br>Show the field ONLY if: [inclusao_arm_1][doen_reuma]='9' and [seg_presenca]='1'	Qual o grau de atividade da doença?<br>*Indique um valor de 0 a 10 0 = ausência de atividade e 10= atividade intensa.*	text (integer, Min: 0, Max: 10)
789	seg_basdai9<br>Show the field ONLY if: [inclusao_arm_1][doen_reuma]='9' and [seg_presenca]='1'	BASDAI<br>*Apenas números. Separar decimais com ponto (.) BASDAI: Bath Ankyloging Spondylitis Disease Activity Score*	text (number)
790	seg_asdas_vhs9<br>Show the field ONLY if: [inclusao_arm_1][doen_reuma]='9' and [seg_presenca]='1'	ASDAS-VHS<br>*Apenas números, separar decimais por ponto (.) ASDAS VHS: Ankylosing Spondylitis Disease Activity Score calculado com VSH*	text (number)
791	seg_asdas_pcr9<br>Show the field ONLY if: [inclusao_arm_1][doen_reuma]='9' and [seg_presenca]='1'	ASDAS-PCR<br>*Apenas números, separar decimais por ponto (.) ASDAS PCR: Ankylosing Spondylitis Disease Activity Score calculado com PCR*	text (number)
792	seg_piora_drim9<br>Show the field ONLY if: [inclusao_arm_1][doen_reuma]='9' and [seg_presenca]='1'	Houve aparecimento ou piora de outra manifestação de DRIM?	radio<br>1 Sim<br>0 Não<br>Custom alignment: LV<br>Field Annotation: @NOMISSING
793	seg_piora_drim_s9<br>Show the field ONLY if: [seg_piora_drim9]='1'	Qual?<br>*Aparecimento de outras manifestações imunomediadas não listadas acima ou não diretamente relacionada com a doença de base.*	text<br>Field Annotation: @NOMISSING
794	info_seg10<br>Show the field ONLY if: [inclusao_arm_1][doen_reuma]='10' and [seg_presenca]='1'	LUPUS ERITEMATOSO SISTÊMICO (LES) - [event-label]	descriptive<br>Field Annotation: @NOMISSING
795	seg_piora_caso_cov10<br>Show the field ONLY if: [inclusao_arm_1][caso_contr]='1' and [seg_presenca]='1' and ([inclusao_arm_1][doen_reuma]='10' or [seg_predomina14]='10')	Houve piora da atividade da doença depois dos sintomas de COVID-19?	radio<br>1 Sim<br>0 Não<br>Custom alignment: LV<br>Field Annotation: @NOMISSING
796	seg_piora_cont_cov10<br>Show the field ONLY if: [inclusao_arm_1][caso_contr]='2' and ([inclusao_arm_1][doen_reuma]='10' or [seg_predomina14]='10')	Houve piora da atividade da doença nos últimos 3 meses? (caso tenha apresentado sintomas de COVID-19, considere o quadro após os sintomas)	radio<br>1 Sim<br>0 Não<br>Custom alignment: LV<br>Field Annotation: @NOMISSING
797	seg_grau_reuma10<br>Show the field ONLY if: ([inclusao_arm_1][doen_reuma]='10' or [seg_predomina14]='10') and [seg_presenca]='1'	Qual o grau de atividade da doença?<br>*Indique um valor de 0 a 10 0 = ausência de atividade e 10= atividade intensa.*	text (integer, Min: 0, Max: 10)
798	seg_sledai10<br>Show the field ONLY if: ([inclusao_arm_1][doen_reuma]='10' or [seg_predomina14]='10') and [seg_presenca]='1'	SLEDAI-2K modificado atual	text (integer)
799	seg_mud_lab10<br>Show the field ONLY if: ([inclusao_arm_1][doen_reuma]='10' or [seg_predomina14]='10') and [seg_presenca]='1'	Houve alguma mudança dos parâmetros laboratoriais específicos na visita atual?	radio<br>1 Sim<br>0 Não<br>999 Exames atuais não disponíveis<br>Custom alignment: LV<br>Field Annotation: @NOMISSING

800	seg_mud_lab_s10<br>Show the field ONLY if:<br>[seg_mud_lab10]='1' and [seg_presenca]='1'	Qual mudança?	checkbox<br>1 seg_mud_lab_s10___1 Anti-DNA<br>2 seg_mud_lab_s10___2 Anti-cardiolipina<br>3 seg_mud_lab_s10___3 Crioglobulinas<br>4 seg_mud_lab_s10___4 Complemento<br>Custom alignment: LV<br>Field Annotation: @NOMISSING
801	seg_dna10<br>Show the field ONLY if:<br>[seg_mud_lab_s10(1)]='1'	Anti-DNA	radio<br>1 Aumento título<br>2 Redução título<br>0 Inalterado<br>Custom alignment: LV<br>Field Annotation: @NOMISSING
802	seg_cardio10<br>Show the field ONLY if:<br>[seg_mud_lab_s10(2)]='1'	Anti-cardiolipina	radio<br>1 Aumento<br>2 Redução<br>0 Inalterado<br>Custom alignment: LV<br>Field Annotation: @NOMISSING
803	seg_criog10<br>Show the field ONLY if:<br>[seg_mud_lab_s10(3)]='1'	Crioglobulinas	radio<br>1 Aumento<br>2 Redução<br>0 Inalterado<br>Custom alignment: LV<br>Field Annotation: @NOMISSING
804	seg_compl10<br>Show the field ONLY if:<br>[seg_mud_lab_s10(4)]='1'	Complemento	radio<br>1 Consumo<br>0 Inalterado<br>Custom alignment: LV<br>Field Annotation: @NOMISSING
805	seg_trombo10<br>Show the field ONLY if:<br>([inclusao_arm_1][doen_reuma]='10' or [seg_predomina14]='10') and [seg_presenca]='1'	Houve alguma nova manifestação trombótica?	radio<br>1 Sim<br>0 Não<br>Custom alignment: LV<br>Field Annotation: @NOMISSING
806	seg_trombo_s10<br>Show the field ONLY if:<br>[seg_trombo10]='1'	Qual?	radio<br>1 Arterial<br>2 Obstétrica<br>3 Venosa<br>Custom alignment: LV<br>Field Annotation: @NOMISSING
807	seg_piora_drim10<br>Show the field ONLY if:<br>([inclusao_arm_1][doen_reuma]='10' or [seg_predomina14]='10') and [seg_presenca]='1'	Houve aparecimento ou piora de outra manifestação de DRIM?	radio<br>1 Sim<br>0 Não<br>Custom alignment: LV<br>Field Annotation: @NOMISSING
808	seg_piora_drim_s10<br>Show the field ONLY if:<br>[seg_piora_drim10]='1'	Qual?<br>*Aparecimento de outras manifestações imunomediadas não listadas acima ou não diretamente relacionada com a doença de base.*	text<br>Field Annotation: @NOMISSING

809	info_seg11<br>Show the field ONLY if: [inclusao_arm_1][doen_reuma]='11' and [seg_presenca]='1'	MIOPATIA INFLAMATÓRIA (DERMATOMIOSITE) - [event-label]	descriptive<br>Field Annotation: @NOMISSING
810	seg_piora_caso_cov11<br>Show the field ONLY if: [inclusao_arm_1][caso_contr]='1' and [seg_presenca]='1' and ([inclusao_arm_1][doen_reuma]='11' or [seg_predomina14]='11')	Houve piora da atividade da doença depois dos sintomas de COVID-19?	radio<br>1 Sim<br>0 Não<br>Custom alignment: LV<br>Field Annotation: @NOMISSING
811	seg_piora_cont_cov11<br>Show the field ONLY if: [inclusao_arm_1][caso_contr]='2' and ([inclusao_arm_1][doen_reuma]='11' or [seg_predomina14]='11')	Houve piora da atividade da doença nos últimos 3 meses? (caso tenha apresentado sintomas de COVID-19, considere o quadro após os sintomas)	radio<br>1 Sim<br>0 Não<br>Custom alignment: LV<br>Field Annotation: @NOMISSING
812	seg_grau_reuma11<br>Show the field ONLY if: ([inclusao_arm_1][doen_reuma]='11' or [seg_predomina14]='11') and [seg_presenca]='1'	Qual o grau de atividade da doença?<br>*Indique um valor de 0 a 10 0 = ausência de atividade e 10= atividade intensa.*	text (integer, Min: 0, Max: 10)
813	seg_info_ativ11<br>Show the field ONLY if: [inclusao_arm_1][caso_contr]='1' and [seg_presenca]='1' and ([inclusao_arm_1][doen_reuma]='11' or [seg_predomina14]='11')	Indique o grau de atividade da doença após a COVID-19	descriptive<br>Field Annotation: @NOMISSING
814	seg_ativ_calc11<br>Show the field ONLY if: [inclusao_arm_1][caso_contr]='1' and [seg_presenca]='1' and ([inclusao_arm_1][doen_reuma]='11' or [seg_predomina14]='11')	Calcinose	radio (Matrix)<br>0 Inalterado<br>2 Piorou<br>1 Melhorou<br>Field Annotation: @NOMISSING
815	seg_ativ_enz11<br>Show the field ONLY if: [inclusao_arm_1][caso_contr]='1' and [seg_presenca]='1' and ([inclusao_arm_1][doen_reuma]='11' or [seg_predomina14]='11')	Elevação de enzimas musculares	radio (Matrix)<br>0 Inalterado<br>2 Piorou<br>1 Melhorou<br>Field Annotation: @NOMISSING
816	seg_ativ_got11<br>Show the field ONLY if: [inclusao_arm_1][caso_contr]='1' and [seg_presenca]='1' and ([inclusao_arm_1][doen_reuma]='11' or [seg_predomina14]='11')	Gottron	radio (Matrix)<br>0 Inalterado<br>2 Piorou<br>1 Melhorou<br>Field Annotation: @NOMISSING
817	seg_ativ_heli11<br>Show the field ONLY if: [inclusao_arm_1][caso_contr]='1' and [seg_presenca]='1' and ([inclusao_arm_1][doen_reuma]='11' or [seg_predomina14]='11')	Heliotropo	radio (Matrix)<br>0 Inalterado<br>2 Piorou<br>1 Melhorou<br>Field Annotation: @NOMISSING
818	seg_ativ_musc11<br>Show the field ONLY if: [inclusao_arm_1][caso_contr]='1' and [seg_presenca]='1' and ([inclusao_arm_1][doen_reuma]='11' or [seg_predomina14]='11')	Musculo	radio (Matrix)<br>0 Inalterado<br>2 Piorou<br>1 Melhorou<br>Field Annotation: @NOMISSING

819	seg_ativ_pele11<br>Show the field ONLY if: [inclusao_arm_1][caso_contr]='1' and [seg_presenca]='1' and ([inclusao_arm_1][doen_reuma]='11' or [seg_predomina14]='11')	Pele	radio (Matrix)<br>0 Inalterado<br>2 Piorou<br>1 Melhorou<br>Field Annotation: @NOMISSING
820	seg_ativ_pulm11<br>Show the field ONLY if: [inclusao_arm_1][caso_contr]='1' and [seg_presenca]='1' and ([inclusao_arm_1][doen_reuma]='11' or [seg_predomina14]='11')	Pulmão	radio (Matrix)<br>0 Inalterado<br>2 Piorou<br>1 Melhorou<br>Field Annotation: @NOMISSING
821	seg_ativ_rayn11<br>Show the field ONLY if: [inclusao_arm_1][caso_contr]='1' and [seg_presenca]='1' and ([inclusao_arm_1][doen_reuma]='11' or [seg_predomina14]='11')	Raynaud	radio (Matrix)<br>0 Inalterado<br>2 Piorou<br>1 Melhorou<br>Field Annotation: @NOMISSING
822	seg_piora_drim11<br>Show the field ONLY if: ([inclusao_arm_1][doen_reuma]='11' or [seg_predomina14]='11') and [seg_presenca]='1'	Houve aparecimento ou piora de outra manifestação de DRIM?	radio<br>1 Sim<br>0 Não<br>Custom alignment: LV<br>Field Annotation: @NOMISSING
823	seg_piora_drim_s11<br>Show the field ONLY if: [seg_piora_drim11]='1'	Qual?<br>*Aparecimento de outras manifestações imunomediadas não listadas acima ou não diretamente relacionada com a doença de base.*	text<br>Field Annotation: @NOMISSING
824	info_seg12<br>Show the field ONLY if: [inclusao_arm_1][doen_reuma]='12' and [seg_presenca]='1'	SARCOIDOSE - [event-label] Avaliação mais recente e disponível em prontuário, de no máximo 6 meses atrás	descriptive<br>Field Annotation: @NOMISSING
825	seg_grau_reuma12<br>Show the field ONLY if: [inclusao_arm_1][doen_reuma]='12' and [seg_presenca]='1'	Qual o grau de atividade da doença?<br>*Indique um valor de 0 a 10 0 = ausência de atividade e 10= atividade intensa.*	text (integer, Min: 0, Max: 10)
826	seg_piora_caso_cov12<br>Show the field ONLY if: [inclusao_arm_1][caso_contr]='1' and [inclusao_arm_1][doen_reuma]='12' and [seg_presenca]='1'	Houve piora da atividade da doença depois dos sintomas de COVID-19?	radio<br>1 Sim<br>0 Não<br>Custom alignment: LV<br>Field Annotation: @NOMISSING
827	seg_piora_cont_cov12<br>Show the field ONLY if: [inclusao_arm_1][caso_contr]='1' and [inclusao_arm_1][doen_reuma]='12' and [seg_presenca]='1'	Houve piora da atividade da doença nos últimos 3 meses? (caso tenha apresentado sintomas de COVID-19, considere o quadro após os sintomas)	radio<br>1 Sim<br>0 Não<br>Custom alignment: LV<br>Field Annotation: @NOMISSING

828	seg_atividade12<br>Show the field ONLY if:<br>[seg_piora_caso_cov12]='1' or [seg_piora_cont_cov12]='1'	Assinale qual(is) manifestação(ões) da atividade(s) da doença(s)	checkbox<br>1 seg_atividade12___1 Cardíaco<br>2 seg_atividade12___2 Intestinal<br>3 seg_atividade12___3 Linfadenopatia<br>4 seg_atividade12___4 Neurológico<br>5 seg_atividade12___5 Ocular<br>6 seg_atividade12___6 Osteoarticular<br>7 seg_atividade12___7 Pele/subcutâneo<br>8 seg_atividade12___8 Pulmonar<br>777 seg_atividade12___777 Outros<br>Custom alignment: LV<br>Field Annotation: @NOMISSING
829	seg_atividade_out12<br>Show the field ONLY if:<br>[seg_atividade12(777)]='1'	Qual outro envolvimento da doença?	text<br>Field Annotation: @NOMISSING
830	seg_trombo12<br>Show the field ONLY if:<br>[inclusao_arm_1][doen_reuma] ='12' and [seg_presenca]='1'	Houve evento tromboembólico?	radio<br>1 Sim<br>0 Não<br>Custom alignment: LV<br>Field Annotation: @NOMISSING
831	seg_trombo_s12<br>Show the field ONLY if:<br>[seg_trombo12]='1'	Em qual sítio?	checkbox<br>1 seg_trombo_s12___1 AVC<br>2 seg_trombo_s12___2 Oclusão Arterial Aguda<br>3 seg_trombo_s12___3 Retina<br>4 seg_trombo_s12___4 Tromboembolismo Pulmonar<br>5 seg_trombo_s12___5 Tromboflebite superficial<br>6 seg_trombo_s12___6 Trombose Venosa Profunda<br>777 seg_trombo_s12___777 Outros<br>Custom alignment: LV<br>Field Annotation: @NOMISSING
832	seg_trombo_s_out12<br>Show the field ONLY if:<br>[seg_trombo_s12(777)]='1'	Qual outro sítio?	text<br>Field Annotation: @NOMISSING
833	seg_piora_drim12<br>Show the field ONLY if:<br>[inclusao_arm_1][doen_reuma] ='12' and [seg_presenca]='1'	Houve aparecimento ou piora de outra manifestação de DRIM?	radio<br>1 Sim<br>0 Não<br>Custom alignment: LV<br>Field Annotation: @NOMISSING
834	seg_piora_drim_s12<br>Show the field ONLY if:<br>[seg_piora_drim12]='1'	Qual?<br>*Aparecimento de outras manifestações imunomediadas não listadas acima ou não diretamente relacionada com a doença de base.*	text<br>Field Annotation: @NOMISSING
835	info_seg13<br>Show the field ONLY if:<br>[inclusao_arm_1][doen_reuma] ='13' and [seg_presenca]='1'	SÍNDROME DE SJÖGREN PRIMÁRIA - [event-label]	descriptive<br>Field Annotation: @NOMISSING

836	seg_piora_caso_cov13<br>Show the field ONLY if: [inclusao_arm_1][caso_contr]='1' and [seg_presenca]='1' and ([inclusao_arm_1][doen_reuma]='13' or [seg_predomina14]='13')	Houve piora da atividade da doença depois dos sintomas de COVID-19?	radio<br>1 Sim<br>0 Não<br>Custom alignment: LV<br>Field Annotation: @NOMISSING
837	seg_piora_cont_cov13<br>Show the field ONLY if: [inclusao_arm_1][caso_contr]='2' and [seg_presenca]='1' and ([inclusao_arm_1][doen_reuma]='13' or [seg_predomina14]='13')	Houve piora da atividade da doença nos últimos 3 meses? (caso tenha apresentado sintomas de COVID-19, considere o quadro após os sintomas)	radio<br>1 Sim<br>0 Não<br>Custom alignment: LV
838	seg_grau_reuma13<br>Show the field ONLY if: ([inclusao_arm_1][doen_reuma]='13' or [seg_predomina14]='13') and [seg_presenca]='1'	Qual o grau de atividade da doença?<br>*Indique um valor de 0 a 10 0 = ausência de atividade e 10= atividade intensa.*	text (integer, Min: 0, Max: 10)<br>Field Annotation: @NOMISSING
839	seg_info_ativ13<br>Show the field ONLY if: ([inclusao_arm_1][doen_reuma]='13' or [seg_predomina14]='13') and [seg_presenca]='1'	Em relação às manifestações clínicas iniciais, indique o grau de atividade neste momento	descriptive<br>Field Annotation: @NOMISSING
840	seg_ativ_artrit13<br>Show the field ONLY if: ([inclusao_arm_1][doen_reuma]='13' or [seg_predomina14]='13') and [seg_presenca]='1'	Artrite	radio (Matrix)<br>0 Inalterado<br>2 Piorou<br>1 Melhorou<br>Field Annotation: @NOMISSING
841	seg_ativ_boca13<br>Show the field ONLY if: ([inclusao_arm_1][doen_reuma]='13' or [seg_predomina14]='13') and [seg_presenca]='1'	Boca seca	radio (Matrix)<br>0 Inalterado<br>2 Piorou<br>1 Melhorou<br>Field Annotation: @NOMISSING
842	seg_ativ_gland13<br>Show the field ONLY if: ([inclusao_arm_1][doen_reuma]='13' or [seg_predomina14]='13') and [seg_presenca]='1'	Glândular	radio (Matrix)<br>0 Inalterado<br>2 Piorou<br>1 Melhorou<br>Field Annotation: @NOMISSING
843	seg_ativ_hemat13<br>Show the field ONLY if: ([inclusao_arm_1][doen_reuma]='13' or [seg_predomina14]='13') and [seg_presenca]='1'	Hematológico	radio (Matrix)<br>0 Inalterado<br>2 Piorou<br>1 Melhorou<br>Field Annotation: @NOMISSING
844	seg_ativ_neuro13<br>Show the field ONLY if: ([inclusao_arm_1][doen_reuma]='13' or [seg_predomina14]='13') and [seg_presenca]='1'	Neuropatia periférica	radio (Matrix)<br>0 Inalterado<br>2 Piorou<br>1 Melhorou<br>Field Annotation: @NOMISSING

845	seg_ativ_olho13<br>Show the field ONLY if: ([inclusao_arm_1][doen_reuma]='13' or [seg_predomina14]='13') and [seg_presenca]='1'	Olho seco	radio (Matrix)<br>0 Inalterado<br>2 Piorou<br>1 Melhorou<br>Field Annotation: @NOMISSING
846	seg_ativ_pele13<br>Show the field ONLY if: ([inclusao_arm_1][doen_reuma]='13' or [seg_predomina14]='13') and [seg_presenca]='1'	Pele	radio (Matrix)<br>0 Inalterado<br>2 Piorou<br>1 Melhorou<br>Field Annotation: @NOMISSING
847	seg_ativ_pulm13<br>Show the field ONLY if: ([inclusao_arm_1][doen_reuma]='13' or [seg_predomina14]='13') and [seg_presenca]='1'	Pulmonar	radio (Matrix)<br>0 Inalterado<br>2 Piorou<br>1 Melhorou<br>Field Annotation: @NOMISSING
848	seg_ativ_renal13<br>Show the field ONLY if: ([inclusao_arm_1][doen_reuma]='13' or [seg_predomina14]='13') and [seg_presenca]='1'	Renal	radio (Matrix)<br>0 Inalterado<br>2 Piorou<br>1 Melhorou<br>Field Annotation: @NOMISSING
849	seg_ativ_nerv13<br>Show the field ONLY if: ([inclusao_arm_1][doen_reuma]='13' or [seg_predomina14]='13') and [seg_presenca]='1'	Sistema nervoso central	radio (Matrix)<br>0 Inalterado<br>2 Piorou<br>1 Melhorou<br>Field Annotation: @NOMISSING
850	seg_ativ_vasc13<br>Show the field ONLY if: ([inclusao_arm_1][doen_reuma]='13' or [seg_predomina14]='13') and [seg_presenca]='1'	Vascular	radio (Matrix)<br>0 Inalterado<br>2 Piorou<br>1 Melhorou<br>Field Annotation: @NOMISSING
851	seg_essdai_s13<br>Show the field ONLY if: ([inclusao_arm_1][doen_reuma]='13' or [seg_predomina14]='13') and [seg_presenca]='1'	ESSDAI atual<br>*Apenas números ESSDAI: EULAR Sjogren's Syndrome Disease Activity Index*	text (integer)
852	seg_piora_drim13<br>Show the field ONLY if: ([inclusao_arm_1][doen_reuma]='13' or [seg_predomina14]='13') and [seg_presenca]='1'	Houve aparecimento ou piora de outra manifestação de DRIM?	radio<br>1 Sim<br>0 Não<br>Custom alignment: LV<br>Field Annotation: @NOMISSING
853	seg_piora_drim_s13<br>Show the field ONLY if: [seg_piora_drim13]='1'	Qual?<br>*Aparecimento de outras manifestações imunomediadas não listadas acima ou não diretamente relacionada com a doença de base.*	text<br>Field Annotation: @NOMISSING
854	info_seg14<br>Show the field ONLY if: [inclusao_arm_1][doen_reuma]='14' and [seg_presenca]='1'	SÍNDROME DE SOBREPOSIÇÃO - [event-label]	descriptive<br>Field Annotation: @NOMISSING

855	seg_predomina14<br>Show the field ONLY if: [inclusao_arm_1][doen_reuma]='14' and [seg_presenca]='1'	Assinale qual a doença predominante	radio<br>4 Artrite reumatoide<br>8 Esclerose sistêmica<br>10 Lupus eritematoso sistêmico<br>11 Miopatia inflamatória<br>13 Síndrome de Sjögren<br>Custom alignment: LV<br>Field Annotation: @NOMISSING
856	info_seg15<br>Show the field ONLY if: [inclusao_arm_1][doen_reuma]='15' and [seg_presenca]='1'	SÍNDROME DO ANTICORPO ANTIFOSFOLÍPIDE PRIMÁRIA - [event-label]	descriptive<br>Field Annotation: @NOMISSING
857	seg_piora_caso_cov15<br>Show the field ONLY if: [inclusao_arm_1][caso_contr]='1' and [inclusao_arm_1][doen_reuma]='15' and [seg_presenca]='1'	Houve piora da atividade da doença depois dos sintomas de COVID-19?	radio<br>1 Sim<br>0 Não<br>Custom alignment: LV<br>Field Annotation: @NOMISSING
858	seg_piora_cont_cov15<br>Show the field ONLY if: [inclusao_arm_1][caso_contr]='2' and [inclusao_arm_1][doen_reuma]='15' and [seg_presenca]='1'	Houve piora da atividade da doença nos últimos 3 meses? (caso tenha apresentado sintomas de COVID-19, considere o quadro após os sintomas)	radio<br>1 Sim<br>0 Não<br>Custom alignment: LV<br>Field Annotation: @NOMISSING
859	seg_trombo15<br>Show the field ONLY if: [inclusao_arm_1][doen_reuma]='15' and [seg_presenca]='1'	Apresentou novo evento tromboembólico?	radio<br>1 Sim<br>0 Não<br>Custom alignment: LV<br>Field Annotation: @NOMISSING
860	seg_trombo_sitio15<br>Show the field ONLY if: [seg_trombo15]='1'	Em qual sítio?	checkbox<br>1 seg_trombo_sitio15___1 AVC<br>2 seg_trombo_sitio15___2 Oclusão Arterial Aguda<br>3 seg_trombo_sitio15___3 Retina<br>4 seg_trombo_sitio15___4 Tromboembolismo Pulmonar<br>5 seg_trombo_sitio15___5 Tromboflebite superficial<br>6 seg_trombo_sitio15___6 Trombose Venosa Profunda<br>777 seg_trombo_sitio15___777 Outros<br>Custom alignment: LV<br>Field Annotation: @NOMISSING
861	seg_trombo_sitio_out15<br>Show the field ONLY if: [seg_trombo_sitio15(777)]='1'	Qual outro sítio?	text<br>Field Annotation: @NOMISSING
862	seg_lab_mudanca15<br>Show the field ONLY if: [inclusao_arm_1][doen_reuma]='15' and [seg_presenca]='1'	Houve alguma mudança dos parâmetros laboratoriais específicos na visita atual?	radio<br>1 Sim<br>0 Não<br>999 Não tenho exames atuais<br>Custom alignment: LV<br>Field Annotation: @NOMISSING

863	seg_lab_anticardio15<br>Show the field ONLY if:<br>[seg_lab_mudanca15]='1'	Anticardiolipina	radio (Matrix)<br>0 Inalterado<br>2 Piorou<br>1 Melhorou<br><br>Custom alignment: LV<br>Field Annotation: @NOMISSING
864	seg_lab_antib2_15<br>Show the field ONLY if:<br>[seg_lab_mudanca15]='1'	Anti-B2 glicoproteína	radio (Matrix)<br>0 Inalterado<br>2 Piorou<br>1 Melhorou<br><br>Custom alignment: LV<br>Field Annotation: @NOMISSING
865	seg_lab_anticoag15<br>Show the field ONLY if:<br>[seg_lab_mudanca15]='1'	Anticoagulante lúpico	radio (Matrix)<br>0 Inalterado<br>2 Piorou<br>1 Melhorou<br><br>Custom alignment: LV<br>Field Annotation: @NOMISSING
866	seg_piora_drim15<br>Show the field ONLY if:<br>[inclusao_arm_1][doen_reuma]<br>='15' and [seg_presenca]='1'	Houve aparecimento ou piora de outra manifestação de DRIM?	radio<br>1 Sim<br>0 Não<br><br>Custom alignment: LV<br>Field Annotation: @NOMISSING
867	seg_piora_drim_s15<br>Show the field ONLY if:<br>[seg_piora_drim15]='1'	Qual?<br>*Aparecimento de outras manifestações imunomediadas não listadas acima ou não diretamente relacionada com a doença de base.*	text<br>Field Annotation: @NOMISSING
868	info_seg16<br>Show the field ONLY if:<br>[inclusao_arm_1][doen_reuma]<br>='16' and [seg_presenca]='1'	VASCULITES - [event-label]	descriptive<br>Field Annotation: @NOMISSING
869	seg_acometim16<br>Show the field ONLY if:<br>[inclusao_arm_1][doen_reuma]<br>='16' and [seg_presenca]='1'	Selecione o acometimento	radio<br>1 Pequenos e Médios Vasos<br>2 Acometimento de Grandes Vasos<br><br>Custom alignment: LV<br>Field Annotation: @NOMISSING
870	info_seg16_peq<br>Show the field ONLY if:<br>[seg_acometim16]='1'	16. VASCULITES - Acometimento de Pequenos e Médios Vasos	descriptive<br>Field Annotation: @NOMISSING
871	seg_piora_peq_cov16<br>Show the field ONLY if:<br>[inclusao_arm_1][caso_contr]<br>='1' and [seg_acometim16]='1'	Houve piora da atividade da doença depois dos sintomas de COVID-19?	radio<br>1 Sim<br>0 Não<br><br>Custom alignment: LV<br>Field Annotation: @NOMISSING
872	seg_piora_cont_cov16<br>Show the field ONLY if:<br>[inclusao_arm_1][caso_contr]<br>='2' and [seg_acometim16]='1'	Houve piora da atividade da doença nos últimos 3 meses? (caso tenha apresentado sintomas de COVID-19, considere o quadro após os sintomas)	radio<br>1 Sim<br>0 Não<br><br>Custom alignment: LV<br>Field Annotation: @NOMISSING
873	seg_peq_grau_reuma16<br>Show the field ONLY if:<br>[seg_acometim16]='1'	Qual o grau de atividade da doença?<br>*Indique um valor de 0 a 10 0 = ausência de atividade e 10= atividade intensa.*	text (integer, Min: 0, Max: 10)

874	seg_peq_bvas16<br>Show the field ONLY if: [seg_acometim16]='1'	BVAS (versão 3)<br>*Apenas números. BVAS: Birmingham Vasculitis Activity Score*	text (integer)
875	seg_peq_pan16<br>Show the field ONLY if: [seg_acometim16]='1'	Five-Factor Score (PAN, GEPA e GPA):<br>*PAN: Poliarterite Nodosa GEPA: Granulomatose Eosinofílica com Poliangeíte GPA: Granulomatose com Poliangeíte*	text (integer)
876	seg_peq_ativid16<br>Show the field ONLY if: [seg_acometim16]='1'	Em relação às manifestações clínicas iniciais, indique o grau de atividade neste momento	descriptive<br>Field Annotation: @NOMISSING
877	seg_peq_articular16<br>Show the field ONLY if: [seg_acometim16]='1'	Articular	radio (Matrix)<br>0 Inalterado<br>2 Piorou<br>1 Melhorou<br>Field Annotation: @NOMISSING
878	seg_peq_cardio16<br>Show the field ONLY if: [seg_acometim16]='1'	Cardiovascular	radio (Matrix)<br>0 Inalterado<br>2 Piorou<br>1 Melhorou<br>Field Annotation: @NOMISSING
879	seg_peq_cutanea16<br>Show the field ONLY if: [seg_acometim16]='1'	Cutânea	radio (Matrix)<br>0 Inalterado<br>2 Piorou<br>1 Melhorou<br>Field Annotation: @NOMISSING
880	seg_peq_gastro16<br>Show the field ONLY if: [seg_acometim16]='1'	Gastrointestinal	radio (Matrix)<br>0 Inalterado<br>2 Piorou<br>1 Melhorou<br>Field Annotation: @NOMISSING
881	seg_peq_ocular16<br>Show the field ONLY if: [seg_acometim16]='1'	Ocular	radio (Matrix)<br>0 Inalterado<br>2 Piorou<br>1 Melhorou<br>Field Annotation: @NOMISSING
882	seg_peq_pulmonar16<br>Show the field ONLY if: [seg_acometim16]='1'	Pulmonar	radio (Matrix)<br>0 Inalterado<br>2 Piorou<br>1 Melhorou<br>Field Annotation: @NOMISSING
883	seg_peq_renal16<br>Show the field ONLY if: [seg_acometim16]='1'	Renal	radio (Matrix)<br>0 Inalterado<br>2 Piorou<br>1 Melhorou<br>Field Annotation: @NOMISSING

884	seg_peq_sn16<br>Show the field ONLY if:<br>[seg_acometim16]='1'	SN periférico	radio (Matrix)<br>0 Inalterado<br>2 Piorou<br>1 Melhorou<br>Field Annotation: @NOMISSING
885	seg_peq_snc16<br>Show the field ONLY if:<br>[seg_acometim16]='1'	SNC	radio (Matrix)<br>0 Inalterado<br>2 Piorou<br>1 Melhorou<br>Field Annotation: @NOMISSING
886	seg_peq_aerea16<br>Show the field ONLY if:<br>[seg_acometim16]='1'	Vias aéreas superiores	radio (Matrix)<br>0 Inalterado<br>2 Piorou<br>1 Melhorou<br>Field Annotation: @NOMISSING
887	info_seg16_gde<br>Show the field ONLY if:<br>[seg_acometim16]='2'	16. VASCULITES - Acometimento de Grandes Vasos	descriptive<br>Field Annotation: @NOMISSING
888	seg_piora_gde_cov16<br>Show the field ONLY if:<br>[inclusao_arm_1][caso_contr]='1' and [seg_acometim16]='2'	Houve piora da atividade da doença depois dos sintomas de COVID-19?	radio<br>1 Sim<br>0 Não<br>Custom alignment: LV<br>Field Annotation: @NOMISSING
889	seg_piora_gde_contr_cov16<br>Show the field ONLY if:<br>[inclusao_arm_1][caso_contr]='2' and [seg_acometim16]='2'	Houve piora da atividade da doença nos últimos 3 meses? (caso tenha apresentado sintomas de COVID-19, considere o quadro após os sintomas)	radio<br>1 Sim<br>0 Não<br>Custom alignment: LV<br>Field Annotation: @NOMISSING
890	seg_info_ativ16<br>Show the field ONLY if:<br>[seg_acometim16]='2'	Atividade de doença	descriptive<br>Field Annotation: @NOMISSING
891	seg_gde_ativ16<br>Show the field ONLY if:<br>[seg_acometim16]='2'	Clínico	radio (Matrix)<br>0 Inalterado<br>2 Piorou<br>1 Melhorou<br>Custom alignment: LV<br>Field Annotation: @NOMISSING
892	seg_gde_lab16<br>Show the field ONLY if:<br>[seg_acometim16]='2'	Laboratorial	radio (Matrix)<br>0 Inalterado<br>2 Piorou<br>1 Melhorou<br>Custom alignment: LV<br>Field Annotation: @NOMISSING
893	seg_img16<br>Show the field ONLY if:<br>[seg_acometim16]='2'	Imagem?	radio (Matrix)<br>0 Inalterado<br>2 Piorou<br>1 Melhorou<br>Custom alignment: LV<br>Field Annotation: @NOMISSING

894	seg_img_s16<br>Show the field ONLY if:<br>[seg_img16]='1'	Qual?	radio<br>1 Angio-RM<br>2 Angio-RM<br>3 US<br><br>Custom alignment: LV<br>Field Annotation: @NOMISSING
895	seg_piora_vasc16<br>Show the field ONLY if:<br>[seg_acometim16]='2'	Houve aparecimento ou piora de outra manifestação de vasculite?	radio<br>1 Sim<br>0 Não<br><br>Custom alignment: LV<br>Field Annotation: @NOMISSING
896	seg_piora_lab16<br>Show the field ONLY if:<br>[seg_acometim16]='2'	Houve mudança dos parâmetros laboratoriais:	radio<br>1 Sim<br>0 Não<br><br>Custom alignment: LV<br>Field Annotation: @NOMISSING
897	seg_anca_c16<br>Show the field ONLY if:<br>[seg_piora_lab16]='1'	ANCA-C (IMF)	radio<br>1 Positivo<br>0 Negativo<br>999 Não disponível<br><br>Custom alignment: LV<br>Field Annotation: @NOMISSING
898	seg_anca_c_valor16<br>Show the field ONLY if:<br>[seg_anca_c16]='1'	ANCA-C (IMF) valor	text (integer)
899	seg_anca_p16<br>Show the field ONLY if:<br>[seg_piora_lab16]='1'	ANCA-P (IMF)	radio<br>1 Positivo<br>0 Negativo<br>999 Não disponível<br><br>Custom alignment: LV<br>Field Annotation: @NOMISSING
900	seg_anca_p_valor16<br>Show the field ONLY if:<br>[seg_anca_p16]='1'	ANCA-P (IMF) valor	text (integer)
901	seg_anca_pr16<br>Show the field ONLY if:<br>[seg_piora_lab16]='1'	ANCA-PR3	radio<br>1 Positivo<br>0 Negativo<br>999 Não disponível<br><br>Custom alignment: LV<br>Field Annotation: @NOMISSING
902	seg_anca_pr_valor16<br>Show the field ONLY if:<br>[seg_anca_pr16]='1'	ANCA-PR3 valor	text (integer)
903	seg_anca_mp16<br>Show the field ONLY if:<br>[seg_piora_lab16]='1'	ANCA-MPO	radio<br>1 Positivo<br>0 Negativo<br>999 Não disponível<br><br>Custom alignment: LV<br>Field Annotation: @NOMISSING

904	seg_anca_mp_valor16<br><br>Show the field ONLY if:<br>[seg_anca_mp16]='1'	ANCA-MPO valor	text (integer)
905	seg_lab_vhs16<br><br>Show the field ONLY if:<br>[seg_piora_lab16]='1'	VHS	text (integer)
906	seg_lab_pcr16<br><br>Show the field ONLY if:<br>[seg_piora_lab16]='1'	PCR	text (integer)
907	seg_piora_drim16<br><br>Show the field ONLY if:<br>[seg_acometim16]='1' or [seg_acometim16]='2'	Houve aparecimento ou piora de outra manifestação de DRIM?	radio<br>1 Sim<br>0 Não<br><br>Custom alignment: LV<br>Field Annotation: @NOMISSING
908	seg_piora_drim_s16<br><br>Show the field ONLY if:<br>[seg_piora_drim16]='1'	Qual?<br>*Aparecimento de outras manifestações imunomediadas não listadas acima ou não diretamente relacionada com a doença de base.*	text<br>Field Annotation: @NOMISSING
909	seg_info_tratamento<br><br>Show the field ONLY if:<br>[seg_presenca]='1'	TRATAMENTO DA DOENÇA REUMÁTICA	descriptive<br>Field Annotation: @NOMISSING
910	info_med	Medicamentos prescritos hoje deverão ser incluídos na próxima visita - 6 meses<br><br>Inclua os medicamentos prescritos na visita anterior (3 meses) que o paciente efetivamente fez uso	descriptive

911	seg_medicam<br><br>Show the field ONLY if:<br>[seg_presenca]='1'	Faz uso de algum medicamento da lista abaixo?	checkbox<br>1 | seg_medicam___1 | Abatacepte<br>2 | seg_medicam___2 | Anti-Il-17 (secuquinumabe, ixezequizumabe)<br>3 | seg_medicam___3 | Anti-IL12/23 (ustequinumabe)<br>4 | seg_medicam___4 | Anti-TNF (infliximabe, etanercepte, adalimumabe, golimumabe, certolizumabe)<br>5 | seg_medicam___5 | Azatioprina<br>6 | seg_medicam___6 | Belimumabe<br>7 | seg_medicam___7 | Ciclofosfamida oral<br>8 | seg_medicam___8 | Ciclosporina<br>9 | seg_medicam___9 | Corticoide oral<br>10 | seg_medicam___10 | Hidroxicloroquina<br>11 | seg_medicam___11 | Inibidor de JAK (tofacitinibe, baricitinibe, upadacitinibe)<br>12 | seg_medicam___12 | Leflunomida<br>13 | seg_medicam___13 | Metotrexato<br>14 | seg_medicam___14 | MMF (Microfenolato de Mofetil)<br>15 | seg_medicam___15 | Pulsoterapia com ciclofosfamida<br>16 | seg_medicam___16 | Pulsoterapia com metiprednisolona<br>17 | seg_medicam___17 | Rituximabe<br>18 | seg_medicam___18 | Sulfassalazina<br>19 | seg_medicam___19 | Tocilizumabe<br>777 | seg_medicam___777 | Outros (detalhar abaixo)<br>99 | seg_medicam___99 | Não sabe ou não respondeu<br>0 | seg_medicam___0 | Nenhuma medicação<br><br>Custom alignment: LV<br>Field Annotation: @NOMISSING
912	seg_outro_med_assoc<br><br>Show the field ONLY if:<br>[seg_medicam(777)] = '1'	Outros medicamentos:<br>*Em caso de mais de um, descrever um por linha*	notes<br>Field Annotation: @NOMISSING
913	seg_dose_cort_oral<br><br>Show the field ONLY if:<br>[seg_medicam(9)] = '1'	Dose corticoide oral	radio<br>1 | até 10 mg/dia<br>2 | >= 11 a 20 mg/dia<br>3 | >= 21 mg/dia<br>99 | Não sabe a dose em uso<br><br>Custom alignment: LV<br>Field Annotation: @NONEOFTHEABOVE='99' @NOMISSING
914	seg_dose_mtx<br><br>Show the field ONLY if:<br>[seg_medicam(13)] = '1'	Dose Metotrexato	radio<br>1 | < = 20 mg/semana<br>2 | >= 21 mg/semana<br>99 | Não sabe a dose em uso<br><br>Custom alignment: LV<br>Field Annotation: @NONEOFTHEABOVE='99' @NOMISSING

915	seg_mudanca<br>Show the field ONLY if:<br>[seg_presenca]='1'	Houve mudança do tratamento em relação a última visita do estudo?<br>*Referente aos últimos 3 meses*	radio<br>1 Sim<br>0 Não<br>Custom alignment: LV<br>Field Annotation: @NOMISSING
916	seg_mudanca_s<br>Show the field ONLY if:<br>[seg_mudanca]='1'	Indique qual a mudança:	checkbox<br>1 seg_mudanca_s___1 Dose medicamento<br>2 seg_mudanca_s___2 Suspensão do medicamento<br>3 seg_mudanca_s___3 Esquema terapêutico<br>777 seg_mudanca_s___777 Outra mudança (detalhar abaixo)<br>Custom alignment: LV<br>Field Annotation: @NOMISSING
917	seg_mudanca_out<br>Show the field ONLY if:<br>[seg_mudanca_s(777)] = '1'	Outra mudança no tratamento<br>*Em caso de mais de uma, descrever uma por linha*	notes<br>Custom alignment: LV<br>Field Annotation: @NOMISSING

918	seg_mudanca_s_retirada<br>Show the field ONLY if:<br>[seg_mudanca_s(2)] = '1' or [seg_mudanca_s(3)] = '1'	Selecione os medicamentos que foram RETIRADOS do esquema terapêutico?	checkbox<br>1 seg_mudanca_s_retirada___1 Abatacepte<br>2 seg_mudanca_s_retirada___2 Anti-Il-17 (secuquinumabe, ixezequizumabe)<br>3 seg_mudanca_s_retirada___3 Anti-IL12/23 (ustequinumabe)<br>4 seg_mudanca_s_retirada___4 Anti-TNF (infliximabe, etanercepte, adalimumabe, golimumabe, certolizumabe)<br>5 seg_mudanca_s_retirada___5 Azatioprina<br>6 seg_mudanca_s_retirada___6 Belimumabe<br>7 seg_mudanca_s_retirada___7 Ciclofosfamida oral<br>8 seg_mudanca_s_retirada___8 Ciclosporina<br>9 seg_mudanca_s_retirada___9 Corticoide oral<br>10 seg_mudanca_s_retirada___10 Hidroxicloroquina<br>11 seg_mudanca_s_retirada___11 Inibidor de JAK (tofacitinibe, baricitinibe, upadacitinibe)<br>12 seg_mudanca_s_retirada___12 Leflunomida<br>13 seg_mudanca_s_retirada___13 Metotrexato<br>14 seg_mudanca_s_retirada___14 MMF<br>15 seg_mudanca_s_retirada___15 Pulsoterapia com ciclofosfamida<br>16 seg_mudanca_s_retirada___16 Pulsoterapia com metiprednisolona<br>17 seg_mudanca_s_retirada___17 Rituximabe<br>18 seg_mudanca_s_retirada___18 Sulfassalazina<br>19 seg_mudanca_s_retirada___19 Tocilizumabe<br>777 seg_mudanca_s_retirada___777 Outros (detalhar abaixo)<br>99 seg_mudanca_s_retirada___99 Não sabe ou não respondeu<br>0 seg_mudanca_s_retirada___0 Nenhuma medicação<br>Custom alignment: LV<br>Field Annotation: @NOMISSING
919	seg_mudanca_s_retirada_out<br>Show the field ONLY if:<br>[seg_mudanca_s_retirada(777)] ='1'	Qual outro medicamento RETIRADO do esquema terapêutico?	text<br>Field Annotation: @NOMISSING

920	seg_mudanca_s_incluido<br><br>Show the field ONLY if: [seg_mudanca_s(2)] = '1' or [seg_mudanca_s(3)] = '1'	Selecione os medicamentos que foram INCLUÍDOS no esquema terapêutico?	checkbox<br>1 seg_mudanca_s_incluido___1 Abatacepte<br>2 seg_mudanca_s_incluido___2 Anti-Il-17 (secuquinumabe, ixezequizumabe)<br>3 seg_mudanca_s_incluido___3 Anti-IL12/23 (ustequinumabe)<br>4 seg_mudanca_s_incluido___4 Anti-TNF (infliximabe, etanercepte, adalimumabe, golimumabe, certolizumabe)<br>5 seg_mudanca_s_incluido___5 Azatioprina<br>6 seg_mudanca_s_incluido___6 Belimumabe<br>7 seg_mudanca_s_incluido___7 Ciclofosfamida oral<br>8 seg_mudanca_s_incluido___8 Ciclosporina<br>9 seg_mudanca_s_incluido___9 Corticoide oral<br>10 seg_mudanca_s_incluido___10 Hidroxicloroquina<br>11 seg_mudanca_s_incluido___11 Inibidor de JAK (tofacitinibe, baricitinibe, upadacitinibe)<br>12 seg_mudanca_s_incluido___12 Leflunomida<br>13 seg_mudanca_s_incluido___13 Metotrexato<br>14 seg_mudanca_s_incluido___14 MMF<br>15 seg_mudanca_s_incluido___15 Pulsoterapia com ciclofosfamida<br>16 seg_mudanca_s_incluido___16 Pulsoterapia com metiprednisolona<br>17 seg_mudanca_s_incluido___17 Rituximabe<br>18 seg_mudanca_s_incluido___18 Sulfassalazina<br>19 seg_mudanca_s_incluido___19 Tocilizumabe<br>777 seg_mudanca_s_incluido___777 Outros (detalhar abaixo)<br>99 seg_mudanca_s_incluido___99 Não sabe ou não respondeu<br>0 seg_mudanca_s_incluido___0 Nenhuma medicação<br><br>Custom alignment: LV<br>Field Annotation: @NOMISSING
921	seg_mudanca_s_incluido_out<br><br>Show the field ONLY if: [seg_mudanca_s_incluido(777)] ='1'	Qual outro medicamento INCLUÍDO no esquema terapêutico?	text<br>Field Annotation: @NOMISSING
922	seg_info_lab_covid<br><br>Show the field ONLY if: [inclusao_arm_1][caso_contr] ='1' and [seg_presenca]='1'	EXAMES LABORATORIAIS Realizados durante a COVID-19	descriptive<br>Field Annotation: @NOMISSING
923	seg_info_lab_covid_2<br><br>Show the field ONLY if: [inclusao_arm_1][caso_contr] ='2' and [seg_presenca]='1'	EXAMES LABORATORIAIS Realizados para controle da doença de base	descriptive<br>Field Annotation: @NOMISSING

924	seg_lab_covid<br>Show the field ONLY if:<br>[seg_presenca]='1'	Exames laboratorias estão disponíveis?	radio<br>1 Sim<br>0 Não<br>Custom alignment: LV<br>Field Annotation: @NOMISSING
925	seg_lab_dt<br>Show the field ONLY if:<br>[seg_lab_covid]='1'	Data da coleta do exame laboratorial	text (date_dmy)<br>Field Annotation: @NOMISSING
926	seg_lab_covid_hb<br>Show the field ONLY if:<br>[seg_lab_covid]='1'	Hb:<br>*Apenas números, separar casas decimais com ponto (.) g/dl*	text (number)
927	seg_lab_covid_hb_dt<br>Show the field ONLY if:<br>[seg_lab_covid]='1'	Data Hb:	text (date_dmy)
928	seg_lab_covid_leuc<br>Show the field ONLY if:<br>[seg_lab_covid]='1'	Leucócitos totais:<br>*Apenas números, separar casas decimais com ponto (.) /mm3*	text (number)
929	seg_lab_covid_neut<br>Show the field ONLY if:<br>[seg_lab_covid]='1'	Neutrófilos:<br>*Valor absolutoApenas números, separar casas decimais com ponto (.) /mm3*	text (number)
930	seg_lab_covid_linfoc<br>Show the field ONLY if:<br>[seg_lab_covid]='1'	Linfócitos:<br>*Valor absolutoApenas números, separar casas decimais com ponto (.) /mm3*	text (number)
931	seg_lab_covid_plaq<br>Show the field ONLY if:<br>[seg_lab_covid]='1'	Plaquetas:<br>*Apenas números, separar casas decimais com ponto (.) /mm3*	text (number)
932	seg_lab_covid_protei_u<br>Show the field ONLY if:<br>[seg_lab_covid]='1'	Proteína C reativa:	radio<br>1 mg/L<br>2 mg/dL<br>Custom alignment: LV
933	seg_lab_covid_protei<br>Show the field ONLY if:<br>[seg_lab_covid_protei_u]!=''	*Apenas números, separar casas decimais com ponto (.)*	text (number)
934	seg_lab_covid_vhs<br>Show the field ONLY if:<br>[seg_lab_covid]='1'	VSH 1ª. Hora<br>*Apenas números, separar casas decimais com ponto (.)*	text (number)
935	seg_lab_covid_alb<br>Show the field ONLY if:<br>[seg_lab_covid]='1'	Albumina:<br>*Apenas números, separar casas decimais com ponto (.) g/dl*	text (number)
936	seg_lab_covid_aldo<br>Show the field ONLY if:<br>[seg_lab_covid]='1'	Aldolase:<br>*Apenas números, separar casas decimais com ponto (.)*	text (number)
937	seg_lab_covid_creat<br>Show the field ONLY if:<br>[seg_lab_covid]='1'	Creatinina:<br>*Apenas números, separar casas decimais com ponto (.) mg/dl*	text (number)
938	seg_lab_covid_dime<br>Show the field ONLY if:<br>[seg_lab_covid]='1'	D-Dímero:<br>*Apenas números, separar casas decimais com ponto (.)*	text (number)
939	seg_lab_covid_desid<br>Show the field ONLY if:<br>[seg_lab_covid]='1'	Desidrogenase lática:<br>*Apenas números, separar casas decimais com ponto (.) mg/dl*	text (number)
940	seg_lab_covid_ferr<br>Show the field ONLY if:<br>[seg_lab_covid]='1'	Ferritina:<br>*Apenas números, separar casas decimais com ponto (.) mg/dl*	text (number)

941	seg_lab_covid_fibri<br>Show the field ONLY if:<br>[seg_lab_covid]='1'	Fibrinogênio:<br>*Apenas números, separar casas decimais com ponto (.)*	text (number)
942	seg_lab_covid_procal<br>Show the field ONLY if:<br>[seg_lab_covid]='1'	Procalcitonina:	text (integer)
943	seg_lab_covid_tgo<br>Show the field ONLY if:<br>[seg_lab_covid]='1'	TGO:	text (integer)
944	seg_lab_covid_tgp<br>Show the field ONLY if:<br>[seg_lab_covid]='1'	TGP:<br>*Apenas números, separar casas decimais com ponto (.) mg/dl*	text (number)
945	seg_lab_covid_trig<br>Show the field ONLY if:<br>[seg_lab_covid]='1'	Triglicerídeos:<br>*Apenas números, separar casas decimais com ponto (.) mg/dl*	text (number)
946	info_visita_seg3<br>Show the field ONLY if:<br>[seg_presenca]='1' and [event-name]='v2_arm_1'	Sobre a visita de seguimento	descriptive
947	seg_v3<br>Show the field ONLY if:<br>[seg_presenca]='1' and [event-name]='v2_arm_1'	Participante de pesquisa irá realizar Visita 3?<br>*Visita será realizada 180 dias após inclusão*	radio<br>1 Sim<br>0 Não<br>Custom alignment: LV
948	seg_v3_nao<br>Show the field ONLY if:<br>[seg_v3]='0'	Porque o participante não irá realizar a visita de seguimento?	radio<br>1 Opção do participante de pesquisa<br>2 Óbito<br>777 Outro motivo<br>Custom alignment: LV
949	seg_v3_nao_obito<br>Show the field ONLY if:<br>[seg_v3_nao] = '2' and [seg_hosp_evol] != '2'	Óbito relacionado a COVID-19?	radio<br>1 Sim<br>0 Não<br>Custom alignment: LV
950	seg_v3_nao_obito_dt<br>Show the field ONLY if:<br>[seg_v3_nao_obito]='1'	Data do óbito relacionado a COVID-19?	text (date_dmy)
951	seg_v3_nao_outro<br>Show the field ONLY if:<br>[seg_v3_nao]='777'	Qual outro motivo o participante de pesquisa não irá participar?	text
952	seguimento_complete	Section Header: *Form Status*<br>Complete?	dropdown<br>0 Incomplete<br>1 Unverified<br>2 Complete
